# Pyridine Into Pyrrole Transformation Induced Within the Confinement of the Macrocycle

**DOI:** 10.1002/anie.202525506

**Published:** 2026-01-30

**Authors:** Paulina Krzyszowska, Agata Burska‐Jabłońska, Mateusz Oberski, Michał J. Białek, Lechosław Latos‐Grażyński, Karolina Hurej

**Affiliations:** ^1^ Department of Chemistry University of Wroclaw Wroclaw Poland

**Keywords:** gold complexes, porphyrin, pyridine, ring contraction, silver complexes

## Abstract

The pyridine contracted to form the pyrrole ring. This transformation belongs to a unique class of reactions with the fundamental characteristic of the cleavage of the aromatic structure. By investigating the unusual coordination chemistry of N‐confused pyriporphyrin with silver and gold ions, we observed this process and obtained several complexes that exhibited remarkable reactivity. This includes the reversible cleavage of C–O bonds and the selective demetallation of the outer metal ion.

## Graphical abstract

This work demonstrates the nontrivial reactivity of pyridine incorporated into a porphyrin framework. The most significant achievement is the contraction of the six‐membered ring to a pyrrole ring, which is a phenomenon of great interest to researchers in this field. In addition, under controlled and mild conditions, the reversible formation of C–O bonds and the coordination of the outer gold ion are also observed.

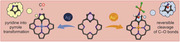

## 1 Introduction

The coordination chemistry of carbaporphyrinoids has become a popular area of research in recent years [1, 2, 3, 4, 5, 6]. These macrocycles are of great interest due to the formation of complexes in which metals acquire unusual oxidation states, and subsequently exhibit nontrivial reactivity associated with the formation of organometallic bonds. Despite numerous studies, the coordination chemistry of pyriporphyrins, i.e., macrocycles in which one of the pyrrole rings is formally replaced by a pyridine ring, is still poorly understood [6, 7, 8].

**FIGURE 1:**
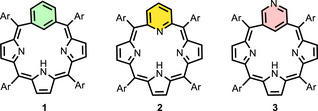
*meta*‐Benziporphyrin **1**, 22‐aza‐*meta*‐benziporphyrin **2** and 3‐aza‐*meta*‐benziporphyrin **3**.

Aza‐*meta*‐benziporphyrins are structural analogues of *meta*‐benziporphyrin (**1**) in which the pyridine motif replaces the benzene moiety, yielding three isomeric structures: 22‐aza‐*m*‐benziporphyrin (**2**) [9], 3‐aza‐*m*‐benziporphyrin (**3**) [10] and 2‐aza‐*m*‐benziporphyrin [11]. The 2‐aza and 3‐aza isomers retain the carbaporphyrinoid features, preserving the characteristic (CNNN) arrangement of inner core donor atoms, whereas the 22‐aza contains the (NNNN) porphyrin‐like donor set (Figure 1) [4, 6].

*m*‐Benziporphyrins **1** are susceptible to unique transformations when stimulated, for example, by the suitable choice of the coordinating metal cation. However, only a few examples of such reactivity have been reported in the literature [4, 12]. For instance, an attempt to incorporate silver ions into *m*‐benziporphyrin resulted in regioselective acetoxylation [12] or pyridination [13], yielding 22‐acetoxy‐*m*‐benziporphyrin and 22‐pyridiniumyl‐*m*‐benziporphyrin, depending on the choice of neutralizing agent (acetate or pyridine). Furthermore, three silver complexes have also been reported for *meta*‐benziporphyrin derivatives: the diamagnetic silver(I) complex of *meso*‐alkylidene *m*‐benziporphyrin, which is stabilized by the coordination of two pyridine molecules acting as axial ligands [14], and the silver(I) porphodimethene and its derivative, which form a dimeric structure consisting of two subunits assembled head‐to‐tail [15]. Lash et al. have also reported a silver(III) complex of the β‐alkylated 2‐oxo‐*m*‐benziporphyrin [16].

Despite the special interest in porphyrin gold complexes [17, 18], the gold chemistry of meta‐benziporphyrin remains a relatively under‐researched area [4]. The cationic complex of *meta*‐benziporphyrin **2** was obtained by inserting gold ions [19]. The use of the methylated derivative of this macrocycle enables the observation of a unique process in which the phenylene ring contracts to form cyclopentadiene derivatives [19]. In addition, the other benziporphyrin isomer, *p*‐benziporphyrin, also undergoes gold ion‐mediated contraction to form 21‐carbaporphyrin complexes [20].

Though **1** and **3** are structurally similar, such reactivities in a macrocyclic framework for pyriporphyrins have not been observed previously. To the best of our knowledge, no silver or gold chemistry with aza‐*meta*‐benziporphyrin has yet been reported.

Understanding the unusual reactivity of pyridine improves our understanding of this fragment's chemistry and facilitates manipulation [21, 22, 23, 24, 25]. The transformation of a six‐membered nitrogen‐containing ring into a five‐membered one is an exceptionally rare type of reactivity. Only a few examples have been reported in the literature, such as the photochemical conversion of azaarene *N*‐oxides into acylazoles [22] or Hantzsch esters and their derivatives, which are then converted into pyrroles via electrochemical extrusion [26].

## 2 Results and Discussion

**SCHEME 1:**
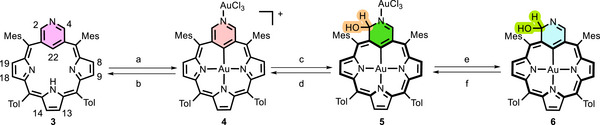
Reactivity of complex **4**. Conditions: (a) Na[AuCl_4_]·H_2_O, toluene, 1 h, reflux, N_2_; (b) Al_2_O_3_, dichloromethane; (c) CDCl_3_, 2,4,6‐collidine, H_2_O; (d) CDCl_3_, DCl; (e) K_2_CO_3_, dichloromethane, 24 h; (f) Na[AuCl_4_]·H_2_O, CDCl_3_.

**FIGURE 2:**
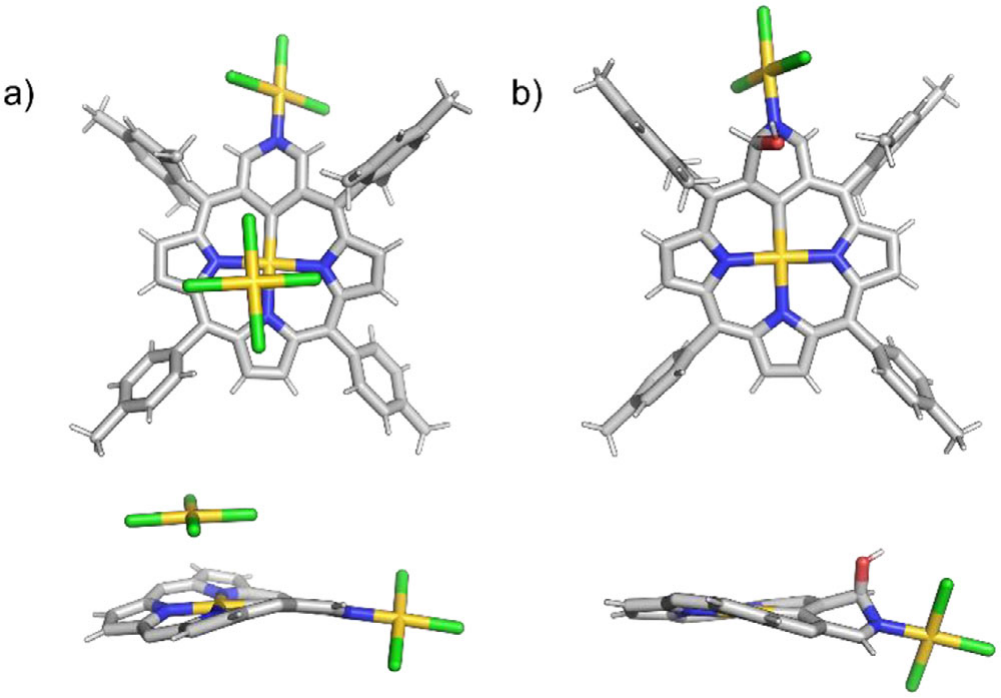
The x‐ray crystal structures of **4** [27] and **5** [28], showing (a) (top) a perspective view of **4**, (a) (bottom) the side view (aryl groups omitted for clarity), (b) (top) a perspective view of **5**, (b) (bottom) the side view (aryl groups omitted for clarity).

**FIGURE 3:**
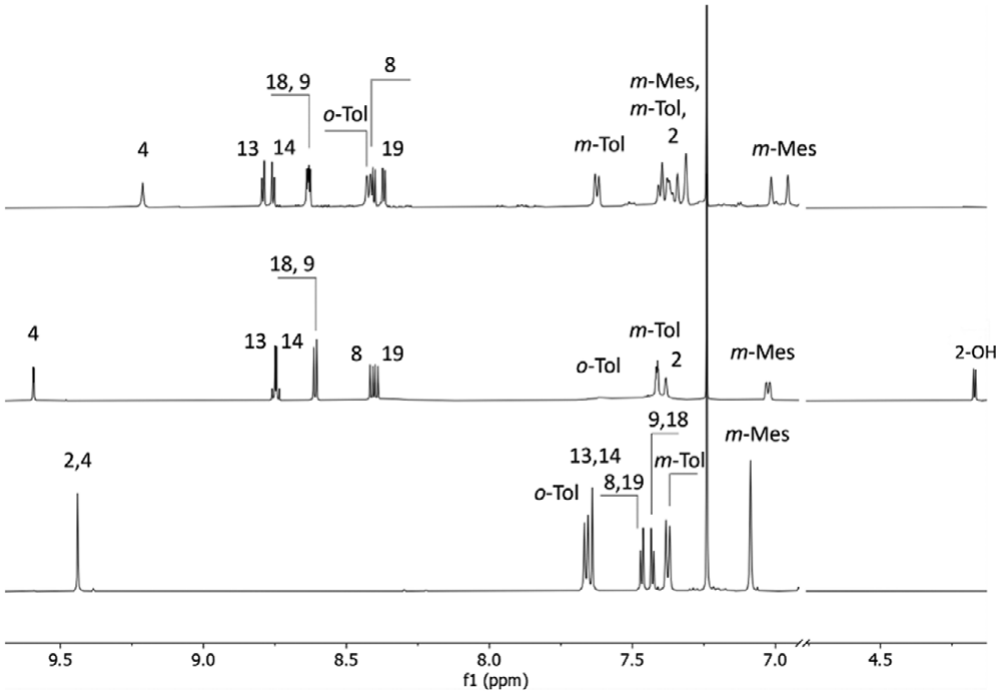
Selected region of the ^1^H NMR spectra of **4** (bottom, 600 MHz, CDCl_3_, 300 K), **5** (middle, 600 MHz, CDCl_3_, 300 K), and **6** (top, 600 MHz, CDCl_3_, 250 K).

The insertion of gold ions into ligand **3** results in the formation of a cationic complex, **4** (Scheme 1). This compound retains the *C_2v_* symmetry characteristic of the starting compound (Figure 3, bottom). The presence of an additional nitrogen atom at the periphery of the 3‐aza‐*m*‐benziporphyrin **3** enables coordination of an additional gold(III) ion. The molecular structure of **4** was confirmed by x‐ray crystallography (Figure 2a). An additional [AuCl_4_]^−^ complex anion can be found in the asymmetric unit, balancing the charge of the macrocycle.

However, compound **4** is highly reactive, and every attempt to purify it has resulted in its conversion into compound **5**. The presence of the OH group in **5**, resulting from the nucleophilic attack of a water molecule on the pyridine ring, disrupts the local aromaticity of the pyridine ring. This is consistent with the global macrocyclic aromaticity of the **5** (6 π vs. 18 π). This enables the observation of a strong aromatic‐ring‐current effect. All pyrrole resonances of **5** are shifted to a higher frequency region (compared to **4**, from δ = 7.43‒7.64 to δ = 8.72‒8.38 ppm). The most structurally informative resonances, H(2), C(2)–OH, and H(4), were identified at *δ =* 7.41 ppm, 4.17 ppm, and 9.59 ppm, respectively (Figure 3, middle). Moreover, the ^13^C chemical shift (based on ^1^H‐^13^C HMBC spectrum) of C(2) (δ = 88.2 ppm) is consistent with its tetrahedral geometry. The molecular structure of **5** (Figure 2b) also clearly shows the deformation of the pyridine moiety induced by the OH addition at position 2. The DFT‐calculated (proton and carbon) chemical shifts of compound **5** (and other derivatives **4–11**) are in agreement with the experimental data (see Figures S67–S73).

A similar switch between local and global aromaticity, induced by hydroxylation, has previously been observed in its relevant derivative 24‐thia‐3‐*aza*‐meta‐benziporphyrin. During protonation with a strong acid, such as trifluoroacetic acid (TFA), a dication forms with an additional proton on one of the nitrogen atoms of the pyrrole and pyridine rings [10].

Compound **4** can be recovered from **5** by adding acid. Neutralizing the acid slowly restores the initial derivative **5**. These mutual transformations can be observed via ^1^H NMR spectroscopy (see Figure S12). Stirring compound **5** with a base such as potassium carbonate in dichloromethane overnight causes the subtraction of the outer [AuCl_3_] unit and yields compound **6**. This process is reversible, and the addition of gold(III) ions (CDCl_3_, RT) results in the formation of complexes **4** and **5** (Figure S13). The ^1^H NMR spectrum pattern of **6** is analogous to that of **5**, with only the protons from the pyridine ring resonating at slightly different positions. The chemical shifts of H(2), C(2)–OH, and H(4) are located at 7.30 ppm, 1.91 ppm, and 9.22 ppm, respectively (Figure 3, top). The ^13^C NMR spectrum (based on ^1^H‐^13^C HMBC spectrum) also shows similar signal positions, with C(2) at 83.0 ppm. Attempts to purify compound **5** or **6** using methanol resulted in the conversion of the hydroxyl (OH) group to a methoxy (OMe) group, forming compound **6**‐*OMe* (see ESI).

**FIGURE 4:**
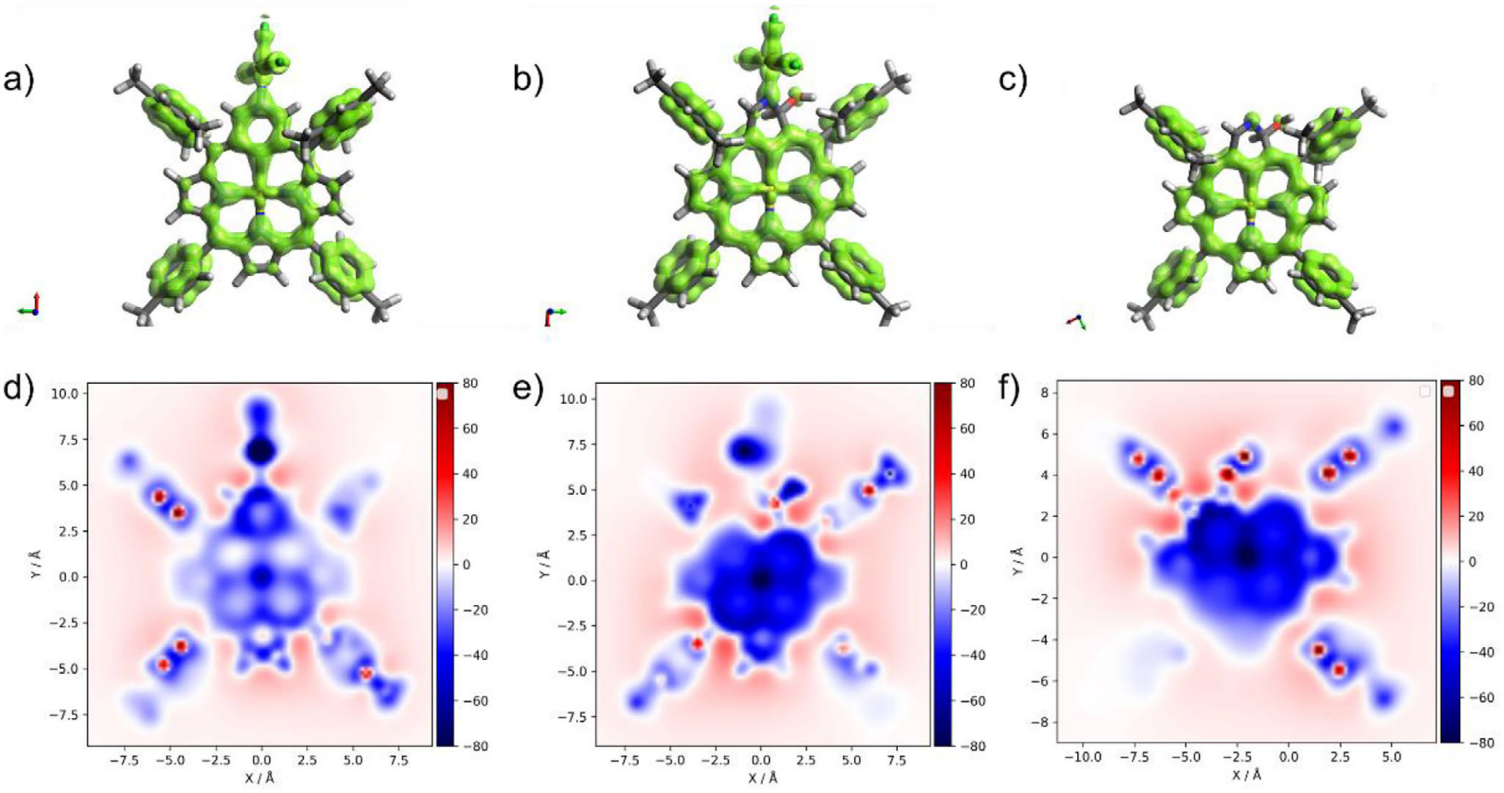
EDDB plots (a–c) and NICS(1)_zz_ 2D maps (d–f) of **4** (a,d), **5** (b,e), and **6** (c,f). In EDDB plots, the localized and delocalized cyclic π‐conjugation is shown as a green surface with an isovalue of 0.025, while NICS values are estimated 1 Å above the mean plane.

The change from local aromaticity of the pyridine ring (as in compound **4**) to the strong aromatic current effect of the macrocycle in compounds **5** and **6** is clearly visible not only in NMR spectroscopy, but also in the EDDB plot and in the NICS(1)_zz_ values (Figure 4), in which one can observe an undisrupted delocalization pathway as well as highly negative NICS values above the macrocycle.

We analyzed the effect of the presence of externally coordinated gold(III) cations on ^15^N chemical shift using ^1^H‐^15^N HMBC correlation spectra. DFT calculations indicated a significant difference in the nitrogen chemical shifts depending on the presence or absence of gold ions. The same trend was observed experimentally (see Figures S8 and S26). While the external gold ion is present, the nitrogen signal exhibits a coordination shift of approximately ‐90 ppm [216 ppm (**5**) versus 310 ppm (**6**‐*OMe*)]. The pyrrole nitrogen atoms resonate at around 135–165 ppm.

**SCHEME 2:**
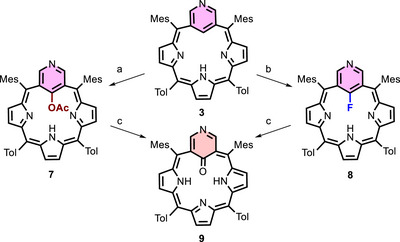
Reactivity of **3** with silver salts. Reaction Conditions: (a) AgOAc, CHCl_3_/MeCN, reflux, 1 h, N_2_, (b) AgF, pyridine, reflux, N_2_, 1 h, (c) SiO_2_, dichloromethane, 48 h.

**SCHEME 3:**
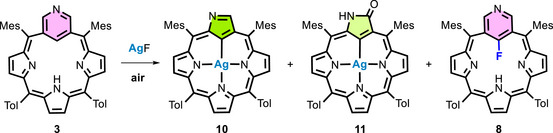
Contraction of the pyridine ring embedded in a porphyrin structure to pyrrole derivatives. Conditions: AgF, pyridine, reflux, air, 15 min.

The chemistry of 3‐aza‐*meta*‐benziporphyrins with silver, a lighter congener of gold, was also interesting. (Schemes 2 and 3). A product analogous to of the one obtained while using *meta*‐benziporphyrin (**1**) could be observed in the presence of silver acetate under similar conditions [11] (Scheme 2).

**FIGURE 5:**
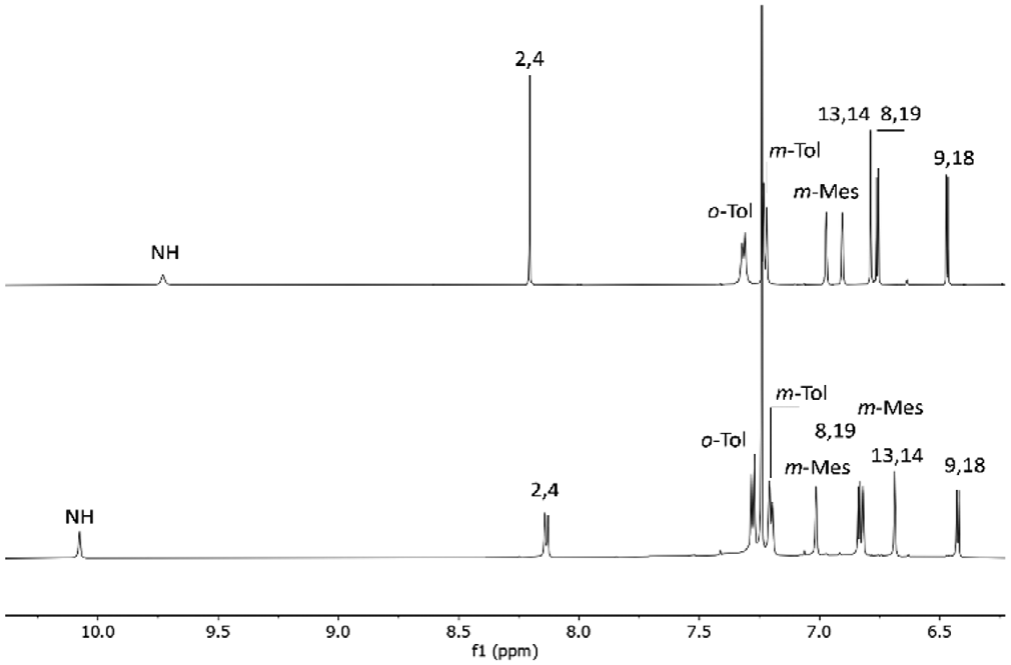
Selected region of the ^1^H NMR spectra of **7** (top) (600 MHz, CDCl_3_, 300 K) and **8** (bottom) (600 MHz, CDCl_3_, 230 K).

22‐Acetoxy‐3‐aza‐*meta*‐benziporphyrin **7** retains the *C_2v_* symmetry of the unmodified molecule. The six‐membered ring retains its local aromaticity, and its signals (2 and 4) resonate at 8.15 ppm (Figure 5, top). The signal from the methyl group of the acetoxy substituent is located at 1.4 ppm. The DFT‐optimized model (Figure S33) of the compound exhibits a geometry consistent with the spectroscopic data obtained experimentally. The composition of the compound was confirmed by mass spectrometry (see ESI).

Macrocycle **3** reacts with silver fluoride in an inert atmosphere to form 22‐fluoro‐3‐aza‐*meta*‐benziporphyrin **8**. The silver complex likely forms only as an intermediate, participating in the transfer of fluoride to the internal carbon atom of the macrocycle via a mechanism in which each step is at thermodynamic equilibrium (see Scheme S1). The presence of the fluorine atom was clearly confirmed by ^19^F NMR spectroscopy, which showed a single signal observed at −101.5 ppm, and by ^1^H‐^19^F and ^13^C‐^19^F correlation spectra (see Figure S37–S39). Another, indirect evidence is the doublet observed at 8.14 ppm [CH(2,4)] in the ^1^H NMR spectrum (Figure 5, bottom), characterized by a high coupling constant of *^4^J_FH_* = 8.3 Hz [29, 30]. In contrast, in the **7**, the signal from position H(2/4) is a singlet due to the substitution at position 22 by an acetoxy group (Figure 5, top). Under similar conditions, this reaction for **1** produces regioselective pyridination of *m*‐benziporphyrin [13].

**FIGURE 6:**
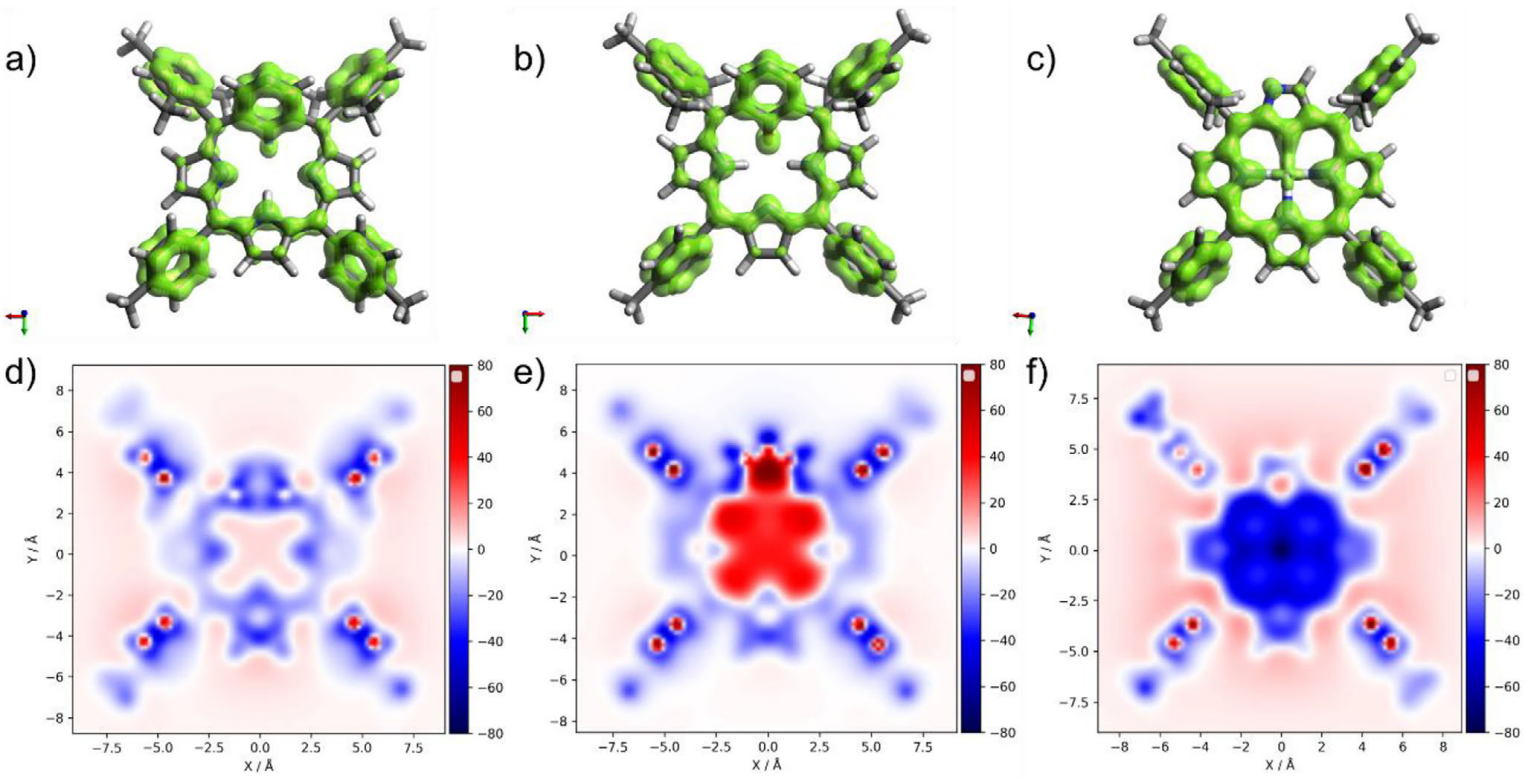
EDDB plots (a–c) and NICS(1)_zz_ 2D maps (d–f) of **8** (a,d), **9** (b,e), and **10** (c,f). In EDDB plots, the localized and delocalized cyclic π‐conjugation is shown as a green surface with an isovalue of 0.025, while NICS values are estimated 1 Å above the mean plane.

The purification of compounds **7** and **8** led to their partial conversion into the paratropic derivative **9**. The ^1^H NMR spectrum of compound **9** shows the characteristic chemical shifts for this type of macrocycle [31]. The pyrrolic protons resonate at 4.86, 5.00 and 5.28 ppm, while the internal NH proton shifts to 22.6 ppm, which is almost 13 ppm higher than the value for compound **7** (9.73 ppm). The paratropic character of compound **9** is clearly demonstrated by the NICS(1)_zz_ map [(see Figure 6b,e) and AICD plot (Figure S47)]. Compared to the starting derivative, 22‐fluoro‐3‐aza‐*meta*‐benziporphyrin **8** [see Figure 6a,d], the NICS(1)_zz_ value has changed to over +35.5 (as indicated by the red color) from around +5.9, as expected for nonaromatic macrocycles.

Carrying out the reaction of **3** with silver fluoride in pyridine in the presence of air results in a different composition of the post‐reaction mixture (Scheme 3). In addition to the expected 22‐fluoro‐3‐aza‐*meta*‐benziporphyrin **8**, two new aromatic compounds, **10** and **11**, were identified. Both obtained compounds contain silver ions in their cavities. However, the most notable aspect is the rearrangement of the macrocycle skeleton. The pyridine ring incorporated into the porphyrin has contracted to form a pyrrole ring, thereby transforming the pyriporphyrin into an N‐confused porphyrin. This change was unambiguously confirmed by spectroscopic analysis, including a direct comparison of the ^1^H NMR spectrum with that of the silver complexes of N‐confused porphyrin **10**, synthesized by rational procedures [32]. The results of HRMS measurements also confirmed the contraction (**10**: *m/z_calc_*_._ for C_52_H_44_N_4_Ag [M+H]^+^ = 831.2611, found = 831.2612; **11**: *m/z_calc._* for C_52_H_43_N_4_OAg [M+H]^+^ = 847.2561, found = 847.2548). In the EDDB plots of **10** (Figure 6c,f), one can see an undisturbed pathway of π‐electron delocalization in the case of contracted NCP, which is in line with its known macrocyclic aromaticity parallel to regular porphyrins. In contrast, for **8** and **9**, plots at the same isovalue levels are partly disturbed.

In derivative **11**, the sole signal in the ^1^H NMR spectrum attributable to an inverted pyrrole ring disappeared upon addition of deuterium oxide, which confirms that it originated from an NH group. The ^13^C NMR spectrum also showed that one of the signals resonates at 168 ppm (see ESI). This reflects the presence of a lactam functionality in silver(III) 3‐oxo‐2‐aza‐21‐carbachlorin [33].

**FIGURE 7:**
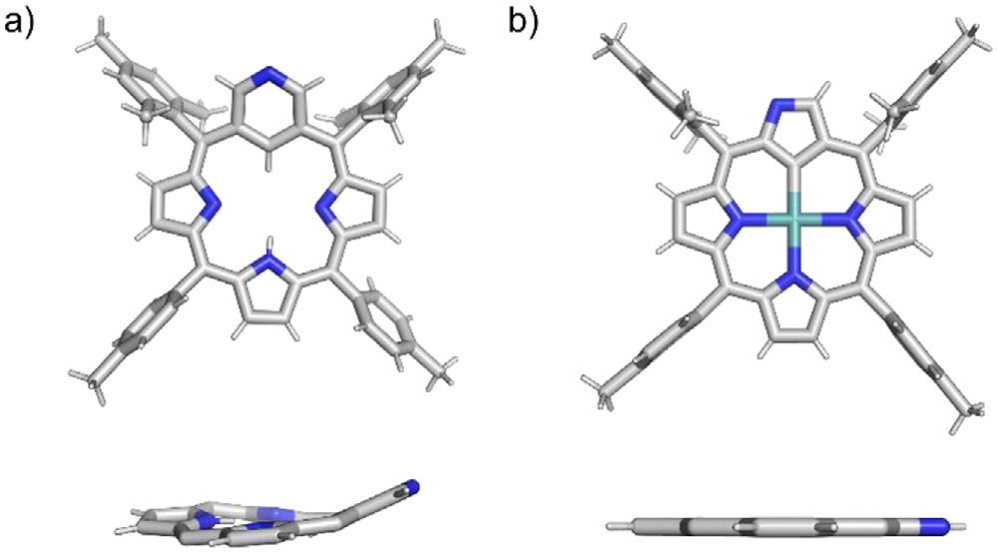
The molecular structures of **3** [36] and **10** [37], showing (a) (top) a perspective view of **3**, (a) (bottom) the side view (aryl groups omitted for clarity), (b) (top) a perspective view of **10**, (b) (bottom) the side view (aryl groups omitted for clarity).

The contraction of the pyridine moiety to a pyrrole ring was confirmed for **10** using single crystal x‐ray diffraction. While the specific 4‐fold symmetry, thus disorder in the crystal, prevents accurate analysis of bond lengths, the obtained data clearly indicate the presence of only five‐membered rings in the macrocycle skeleton. The structure is also noticeably flatter than that of starting compound **3** (Figure 7a,b) [10, 34, 35].

The contraction reaction was followed over time via ^1^H NMR spectroscopy (Py*‐d_5_*, 350 K), allowing us to propose a mechanism for the stepwise transformations of 3‐aza‐*meta*‐benziporphyrin **3**. In the presence of AgF, we firstly observed the formation of 22‐fluoro derivative **8**. However, the subsequently appearing spectroscopic picture was not clear until the contraction products were obtained.

**SCHEME 4:**
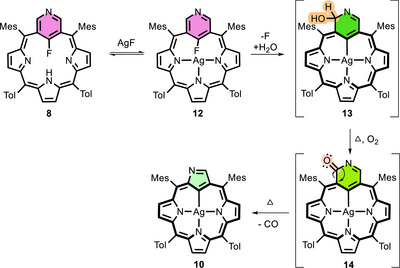
Proposed pathway of the contraction process of derivative **8** to form silver(III) complexes of N‐confused porphyrins.

For this reason, 22‐fluoro‐3‐aza‐*meta*‐benziporphyrin **8** has been used as a starting point for further investigation (Scheme 4). The reaction of **8** with AgF in pyridine at 350 K results in the insertion of the silver(I) ion, yielding **12** in which the fluorine atom is retained in position 22 (see Figure S48). This is evidenced by the characteristic coupling of signals from positions 2,4 (8.2 Hz), originating from ^4^ *J*_H‐F_. The absence of a signal from the pyrrole hydrogen atom may indicate the presence of the silver(I) ion in the porphyrin skeleton. This is the first time that a silver complex has been observed in *meta*‐benziporphyrin derivatives. Over time, the characteristic signals from both compounds disappear (first **8**, then **12**), leaving only the products of contraction processes **10** and **11**.

The above observations suggest that the formation of the postulated silver(III) 3‐aza‐*meta*‐benziporphyrin complex **13** can only occur through a nucleophilic attack of a water molecule on the pyridine ring. This enables the cavity to stabilize the silver ions more snugly, forming an organometallic bond. Subsequently, the C(2)H(OH) unit is rapidly oxidized to form a ketone (**14**). The process must be carried out in air or even with the addition of oxygen. The previously proposed mechanism of *meta*‐phenylene contraction assumed the release of a formaldehyde molecule, which was confirmed by ^1^H NMR spectra [38]. Finally, a contraction process occurs, releasing CO molecules. A similar reaction to that of gold complex **6**, which is analogous to compound **13**, did not result in the contraction process.

## 3 Conclusions

In summary, it is important to note that *p*‐benziporphyrin and *m*‐benziporphyrin, the two isomeric carbaporphyrinoids, have been explored as suitable structural matrices for creating 21‐carbaporphyrin complexes. Notably, this study successfully extends the contraction concept to N‐confused pyriporphyrin. In the presence of silver(I), this compound contracts to form a silver(III) N‐confused porphyrin by extrusion of an α‐carbon atom from the built‐in pyridine unit. Moreover, in this work, the formation of bimetallic gold complexes was confirmed, as well as the ease with which nucleophilic attacks on the pyridine ring could be controlled and reversed. Furthermore, we were able to selectively and reversibly remove the external gold ion. We also synthesized several new 22‐substituted macrocycles: 22‐fluoro‐, 22‐acetoxy‐, and 22‐oxo‐3‐aza‐*meta*‐benziporphyrin.

## Conflicts of Interest

The authors declare no conflict of interest.

## Supporting information




**Supporting File 1**: The authors have cited additional references within the Supporting Information [39–47].

**Supporting File 2**: anie71319‐sup‐0002‐Data.zip.

## Data Availability

The data that support the findings of this study are openly available in Zenodo at https://10.5281/zenodo.17639926, reference number 17639926.
