# Supplementary material for: Pyridine Into Pyrrole Transformation Induced Within the Confinement of the Macrocycle
Source: Angew Chem Int Ed Engl. 2026 Jan 30;65(11):e25506. doi: 10.1002/anie.202525506 (PMC12970504; doi:10.1002/anie.202525506)
Supplement: Supplementary file 1 — Supporting File 1: The authors have cited additional references within the Supporting Information [39–47]. [file ANIE-65-e25506-s002.pdf]

# Pyridine into Pyrrole Transformation Induced within the Confinement of the Macrocycle

Paulina Krzyszowska, Agata Burska-Jabłońska, Mateusz Oberski, Michał J. Białek, Lechosław Latos-Grażyński, Karolina Hurej*

Supporting Information

# Table of contents

Methods ........ 6

X-Ray ........ 6

Theoretical calculations ........ 6

Experimental ........ 8

Figures ........ 14

Figure S1. $^1$H NMR spectrum of 4 (500 MHz, $CDCl_3$, 300 K). ........ 14

Figure S2. Signals assignment; $^1$H NMR spectrum of 4; 500 MHz, $CDCl_3$, 300 K (selected ranges); * - impurities. ........ 15

Figure S3. $^{13}$C NMR spectrum (125 MHz, $CDCl_3$, 300 K) of 4; (top: the whole spectral range, bottom: the most informative region). ........ 16

Figure S4. Selected region of HRMS ESI (+MS) spectrum of 4. ........ 16

Figure S5. UV-Vis spectra of 4 (black line), 5 (red line) and 6 (green line). ........ 17

Figure S6. $^1$H NMR spectrum of 5 (500 MHz, $CDCl_3$, 300 K). ........ 17

Figure S7. Signals assignment; $^1$H NMR spectrum of 5; 500 MHz, $CDCl_3$, 300 K (selected ranges). ........ 18

Figure S8. $^1$H-$^{15}$N HMBC spectrum (600 MHz, $CDCl_3$, 300 K) of 5. ........ 19

Figure S9. $^1$H-$^{13}$C HSQC spectrum (600 MHz, $CDCl_3$, 300 K) of 5 (selected range). ........ 20

Figure S10. $^1$H-$^{13}$C HMBC spectrum (600 MHz, $CDCl_3$, 300 K) of 5 (selected range). ........ 21

Figure S11. Selected regions of HRMS ESI (+MS) spectrum of 5. ........ 22

Figure S12. Interconversion of compounds 5 and 4. ........ 22

Figure S13. Conversion of compound 6 into compounds 4 and 5 in the presence of gold ions. ........ 23

Figure S14. $^1$H NMR spectrum of 6 (500 MHz, $CDCl_3$, 300 K). ........ 23

Figure S15. Signals assignment; $^1$H NMR spectrum of 6; 600 MHz, $CDCl_3$, 250 K (selected ranges). ........ 24

Figure S16. $^1$H-$^1$H NOESY spectrum (600 MHz, $CDCl_3$, 250 K) of 6 (selected range). ........ 24

Figure S17. $^1$H-$^{13}$C HSQC spectrum (600 MHz, $CDCl_3$, 250 K) of 6 (selected range). ........ 25

Figure S18. $^1$H-$^{13}$C HMBC spectrum (600 MHz, $CDCl_3$, 250 K) of 6 (selected range). ........ 25

Figure S19. $^{13}$C NMR spectrum (150 MHz, $CDCl_3$, 300 K) of 6; (top: the whole spectral range, bottom: the most informative region). ........ 26

Figure S20. AICD plot for 6. ........ 26

Figure S21. $^{1}H$ NMR spectrum of 6-OMe (600 MHz, $CDCl_3$, 300 K). .................................... 27

Figure S22. Signals assignment; $^{1}H$ NMR spectrum of 6-*OCH₃*; 500 MHz, $CDCl_3$, 300 K (selected ranges). ..................................................................................... 27

Figure S23. $^{13}C$ NMR spectrum (150 MHz, $CDCl_3$, 300 K) of 6-$OCH_3$; (top: the whole spectral range, bottom: the most informative region). .................................................. 28

Figure S24. $^{1}H$-$^{13}C$ HSQC spectrum (600 MHz, $CDCl_3$, 300 K) of 6-*$OCH_3$* (selected range). .. 28

Figure S25. $^{1}H$-$^{13}C$ HSQC spectrum (600 MHz, $CDCl_3$, 300 K) of 6-*$OCH_3$* (selected range). .. 29

Figure S26. $^{1}H$-$^{15}N$ HMBC spectrum (600 MHz, $CDCl_3$, 300 K) of 6-*$OCH_3$* (selected range). . 30

Figure S27. Selected region of HRMS ESI (+MS) spectrum of 6-*$OCH_3$*. ................................ 30

Figure S28. $^{1}H$ NMR spectrum of 7 (600 MHz, $CDCl_3$, 300 K). ............................................ 31

Figure S29. Signals assignment; $^{1}H$ NMR spectrum of 7; 600 MHz, $CDCl_3$, 300 K (selected ranges). .................................................................................................. 31

Figure S30. $^{13}C$ NMR spectrum (125 MHz, $CDCl_3$, 300 K) of 7; (top: the whole spectral range, bottom: the most informative region). .................................................. 32

Figure S31. Selected region of HRMS ESI (+MS) spectrum of 7. ........................................ 32

Figure S32. UV-Vis spectra of 7 (black line) and 8 (red line). ............................................ 33

Figure S33. The DFT-optimised model of 7 and 8. ............................................................ 33

Figure S34. $^{1}H$ NMR spectrum of 8 (600 MHz, $CD_2Cl_2$, 300 K. ............................................ 34

Figure S35. Signals assignment; 1H NMR spectrum of 8; 500 MHz, CDCl3, 300 K (selected ranges). .................................................................................................. 34

Figure S36. Signals assignment; $^{1}H$ NMR spectrum of 8; 500 MHz, $CDCl_3$, 230 K (selected ranges). .................................................................................................. 35

Figure S37. Signal assignment; $^{19}F$ NMR spectrum of 8; 470 MHz, $CDCl_3$, 300 K (selected range). .................................................................................................. 35

Figure S38. $^{1}H$-$^{19}F$ COSY spectrum (500 MHz, $CDCl_3$, 300 K) of 8. ....................................... 36

Figure S39. $^{13}C$-$^{19}F$ HSQC spectrum (500 MHz, $CDCl_3$, 300 K) of 8. ..................................... 36

Figure S40. $^{13}C$ NMR spectrum (125 MHz, $CD_2Cl_2$, 300 K) of 8; (top: the whole spectral range, bottom: the most informative region). .................................................. 37

Figure S41. Selected region of HRMS ESI (+MS) spectrum of 8. ........................................ 38

Scheme S1. The postulated pathway of forming compound 8. ......................................... 38

Figure S42. $^{1}H$ NMR spectrum of 9 (500 MHz, $C_6D_6$, 300 K). ............................................ 39

Figure S43. Signals assignment; $^{1}H$ NMR spectrum of 9; 500 MHz, $CDCl_3$, 300 K (selected ranges). .................................................................................................. 39

Figure S44. $^{1}H$-$^{13}C$ HSQC spectrum (600 MHz, $CDCl_3$, 300 K) of 9 (selected range). ............ 40

Figure S45. $^{1}H$-$^{13}C$ HMBC spectrum (600 MHz, $CDCl_3$, 300 K) of 9 (selected range). ........... 41
Figure S46. Selected regions of HRMS ESI (+MS) spectrum of 9. ........................................ 42
Figure S47. AICD plot for 9. ........................................................................................ 42
Figure S48. Conversion of compound 8 into compounds 12 and 10 in the presence of silver ions. ........................................................................................................ 43
Figure S49. $^{1}H$ NMR spectrum of 10 (600 MHz, $C_6D_6$, 300 K). ........................................ 43
Figure S50. Signals assignment; $^{1}H$ NMR spectrum of 10; 500 MHz, $CDCl_3$, 300 K (selected ranges). .................................................................................................. 44
Figure S51. Signals assignment; $^{1}H$ NMR spectrum of 10; 500 MHz, $CDCl_3$, 230 K (selected ranges). .................................................................................................. 44
Figure S52. Signals assignment; $^{1}H$ NMR spectrum of 10; 600 MHz, $C_6D_6$, 310 K (selected ranges). .................................................................................................. 45
Figure S53. $^{1}H$-$^{15}N$ HMBC spectrum (600 MHz, $C_6D_6$, 310 K) of 10. ................................... 45
Figure S54. $^{13}C$ NMR spectrum (125 MHz, $CDCl_3$, 300 K) of 10; (top: the whole spectral range, bottom: the most informative region). ........................................................ 46
Figure S55. Selected regions of HRMS ESI (+MS) spectrum of 10. ...................................... 47
Figure S56. IR spectrum of 10. ...................................................................................... 47
Figure S57. $^{1}H$ NMR spectrum of 11 (600 MHz, $CD_2Cl_2$, 300 K). ........................................ 48
Figure S58. Signals assignment; $^{1}H$ NMR spectrum of 11; 500 MHz, $CDCl_3$, 300 K (selected ranges). .................................................................................................. 48
Figure S59. Comparison of selected range $^{1}H$ NMR spectra of 11 before (bottom) and after (top) adding $D_2O$ (500 MHz, $CDCl_3$, 300K). ........................................................ 49
Figure S60. Part of the $^{1}H$ NMR spectra of 10 and 11 (500 MHz, $CDCl_3$, 300 K). ................ 49
Figure S61. $^{13}C$ NMR spectrum (125 MHz, $CDCl_3$, 300 K) of 11; (top: the whole spectral range, bottom: the most informative region). ........................................................ 50
Figure S62. $^{1}H$-$^{13}C$ HMBC spectrum (600 MHz, $CDCl_3$, 300 K) of 11 (selected range). .......... 51
Figure S63. $^{1}H$-$^{15}N$ HMBC spectrum (600 MHz, $CDCl_3$, 300 K) of 11. ................................. 52
Figure S64. Selected regions of HRMS ESI (+MS) spectrum of 11. ...................................... 53
Figure S65. UV-Vis spectra of 8 (blue line), 10 (black line) and 11 (red line). ...................... 53
Figure S66. IR spectrum of 11. ...................................................................................... 54
Cartesian coordinates ................................................................................................ 55
Correlations between calculated and experimental NMR values ...................................... 60
Figure S67. The correlation between calculated and experimental NMR values for 4. ...... 60

Figure S68. The correlation between calculated and experimental NMR values for 5. ...... 60
Figure S69.The correlation between calculated and experimental NMR values for 6. ....... 61
Figure S70. The correlation between calculated and experimental NMR values for 7. ...... 61
Figure S71. The correlation between calculated and experimental NMR values for 8. ...... 62
Figure S72. The correlation between calculated and experimental NMR values for 10. .... 62
Figure S73. The correlation between calculated and experimental NMR values for 11. .... 63
Bibliography.......................................................................................................... 64

## Methods

### Nuclear Magnetic Resonance

NMR spectra were recorded on Bruker Avance III 500 MHz, and 600 MHz spectrometers, and JEOL JNM-ECZ500R 500 MHz spectrometer. Chemical shifts were reported in ppm with reference to residual protons and carbons of $CDCl_3$ (δ 7.24 ppm in $^1H$ NMR), $CD_2Cl_2$ (δ 5.32 ppm in $^1H$ NMR, δ 54.0 ppm in $^{13}C$ NMR), $C_5D_5N$ (δ 8.74 ppm in $^1H$ NMR), and $C_6D_6$ (δ 7.16 ppm in $^1H$ NMR).

### Mass spectrometry

Mass spectra were carried out on a Bruker qTOF compact spectrometer using electrospray ionization.

### UV-Vis spectroscopy

UV-Vis spectra were recorded on a Varian Carry 60 using 1 cm path length optical glass/quartz cuvettes.

### X-Ray

Monocrystals were measured on an XtaLAB Synergy R, DW system, HyPix-Arc 150 diffractometer at 100 K, using CuKα radiation; λ = 1.54184 Å). Data reduction and analysis were carried out with the CrysAlisPro program. An absorption correction was applied. Structures were solved using the SHELXT [1] program and refined using all F2 data, as implemented by the SHELXL program [2]. All non-hydrogen atoms were further refined by SHELXL with anisotropic displacement coefficients; the positions of hydrogen atoms were calculated and refined in a riding mode.

### Infrared Spectroscopy

Attenuated Total Reflectance (ATR) spectra were collected on Nicolet iS10 Spectrometer (Thermo Scientific). The spectra were collected in 4000-400 $cm^{-1}$ range with number of scans set to 64.

### Theoretical calculations

Geometry optimizations were carried out with the Gaussian 16 software package [3] within unconstrained C1 symmetry, with starting coordinates derived from molecular mechanics or X-ray analysis. Becke's three-parameter exchange functionals with the gradient corrected correlation formula of Lee, Yang and Parr (DFT-B3LYP) [4,5] were used with the 6-31G(d,p) basis set with the LANL2DZ pseudopotential applied for Ag and Au centers and the solvation

($PCM=CHCl_3$). Harmonic vibrational frequencies were calculated using analytical second derivatives as a verification of local minimum achievement with no negative frequencies observed. The structures were found to have converged to a minimum on the potential energy. Proton and carbon chemical shifts were calculated using the GIAO method and referenced to the absolute shielding of tetramethylsilane calculated at the same level of theory. The structure models reflect consistently the constrains imposed by the appropriate NOE experiments. The $^{1}H$ chemical shifts calculated for DFT optimized structures of 4–12 remain in a qualitative agreement with the appropriate experimental data. 2D NICS(1)zz map points were generated through the py.Aroma 4.0 tool [6]. EDDB plots were obtained from population analysis (at the ωB97XD/def2sVP level of theory). The resulting data were analyzed through the EDDB program [7-9], and the output was visualized using Avogadro 1.2.

The NMR, HRMS, and DFT data files are available at doi.org/10.5281/zenodo.17639926.

**Experimental**

All solvents (Dichloromethane, Ethyl Acetate, Chloroform, *n*-hexane), if not indicated differently, were used without purification.

Chloroform-$d_1$ and pyridine were prepared directly before use by passing through a basic alumina column.

The compound 3-aza-*meta*-benziporphyrin **3** and its precursors were synthesized according to a literature procedure [10].

**Synthesis of compounds 4, 5 and 6**

Compound **3** (10 mg, $13.5 \cdot 10^{-3}$ mmol) and $Na[AuCl_4] \cdot H_2O$ (20 mg, $5.0 \cdot 10^{-2}$ mmol) dissolved in toluene (10 ml). The mixture was degassed and refluxed for 1 h under nitrogen. Toluene was evaporated on a rotary evaporator. The compound **4** was obtained. During purification on the column chromatography ($SiO_2$, DCM), the compound **4** was partly transformed into **5** and **6**. Yield for 4 – 7.5 mg (45%).

Compound **5** can also be produced again by adding base (2,4,6-collidine, $CDCl_3$, 1 h, NMR tube) to the solution of complex **4**.

Stirring compound **5** with base (like potassium carbonate) in dichloromethane (5 ml) overnight yields compound **6**. Yield – 5.3 mg (70%).

**Compound 4**

**$^1$H NMR (500 MHz, $CDCl_3$, 300 K)** δ 9.44 (s, 2H, 2, 4), 7.67 (AA'XX', 4H, *o*-Tol), 7.64 (s, 2H, 13, 14), 7.47 (d, $^3J_{HH}$ = 5.4 Hz, pyrr), 7.43 (d, $^3J_{HH}$ = 5.4 Hz, pyrr), 7.38 (AA'XX', 4H, *m*-Tol), 7.09 (s, 4H, *m*-Mes), 2.51 (s, 6H, *p*-Mes), 2.56 (s, 6H, *p*-Tol), 1.98 (s, 12H, *o*-Mes).

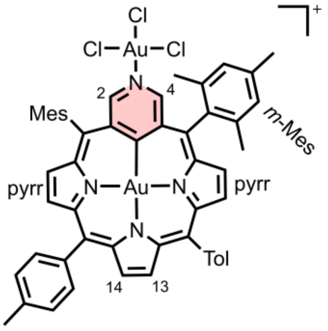


**4**

**$^{13}$C NMR (125 MHz, $CDCl_3$, 300 K)** δ 161.1, 154.8, 151.0, 141.9, 140.9, 139.5, 139.1, 138.5, 138.4, 136.4, 135.4, 134.1, 134.0, 132.6, 132.2, 129.0, 128.3, 121.2, 120.9, 21.59, 21.5, 21.3.

**HRMS (ESI)** *m/z* = 933.3242 calc. for $[C_{53}H_{44}N_4Au]^+$, $[M-AuCl_3]^+$: 933.3226.

**UV-Vis ($CH_2Cl_2$) $\lambda_{max}$ [nm] (logε)** = 326 (3.19), 451 (3.45), 801 (2.45).

**Compound 5**

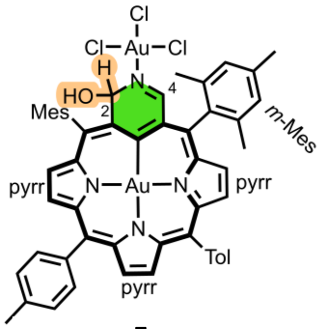


**5**

**$^1$H NMR (500 MHz, CDCl$_3$, 300 K)** δ 9.58 (d, $^3J_{HH}$ = 1.0 Hz, 1H, 2), 8.75 (d, $^3J_{HH}$ = 5.0 Hz, 1H, pyrr), 8.73 (d, $^3J_{HH}$ = 5.0 Hz, 1H, pyrr), 8.60 (d, $^3J_{HH}$ = 5.0 Hz, 1H, pyrr), 8.41 (d, $^3J_{HH}$ = 4.9 Hz, 1H, pyrr), 8.39 (d, $^3J_{HH}$ = 4.9 Hz, 1H, pyrr), 8.36 (brs, 4H, *o*-Tol), 7.61 (brs, 4H, *m*-Tol), 7.41 (s, 2H, *m*-Mes), 7.38 (s, 1H, 4), 7.03 (s, 1H, *m*-Mes), 7.02 (s, 1H, *m*-Mes), 2.64 (s, 3H, *p*-Tol), 2.642 (s, 3H, *p*-Tol), 2.62 (s, 3H, *m*-Mes), 2.57 (s, 3H, *m*-Mes), 2.56 (s, 3H, *m*-Mes), 2.54 (s, 3H, *m*-Mes), 1.13 (s, 3H, *o*-Mes), 1.08 (s, 3H, *o*-Mes).

**$^{13}$C NMR – partial data based on HSQC and HMBC** spectra δ 142.1, 139.4, 139.5, 138.4, 138.2, 137.7, 137.7, 136.4, 136.0, 135.7, 134.3, 134.0, 131.4, 129.8, 129.4, 128.9, 128.6, 128.1, 88.2, 22.6, 22.59, 21.5, 21.48, 20.7, 20.4.

**HRMS (ESI)** *m/z* = 949.3225 calc. for $[C_{53}H_{44}N_4OAu]^+$, $[M{-}AuCl_3{-}H]^+$: 949.3175; m/z = 950.3208 calc. for $[C_{53}H_{45}N_4OAu]^+$, $[M{-}AuCl_3]^+$: 950.3253, m/z = 951.3364, calc. for $[C_{53}H_{46}N_4OAu]^+$, $[M{-}AuCl_3{+}H]^+$: 951.332.

**UV-Vis ($CH_2Cl_2$) $\lambda_{max}$ [nm] (logε)** = 450 (3.74), 530 (2.61), 585 (2.58).

**Compound 6**

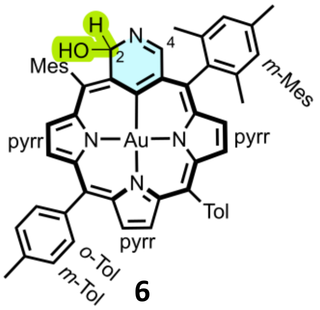


**6**

Compound **5** (10 mg, 8.0·10$^{-3}$ mmol) and $K_2CO_3$ (20 mg, 14.5·10$^{-2}$ mmol) were dissolved in DCM (10 ml). The mixture was degassed and stirred overnight under nitrogen. The solvent was evaporated on a rotary evaporator. Product **6** was subjected to chromatography ($SiO_2$, DCM).

Add $Na[AuCl_4]{\cdot}H_2O$ to solution compound **6** in $CDCl_3$ leads to a mixture of **4** and **5.**

**$^1$H NMR (500 MHz, CDCl$_3$, 250 K)** δ 9.21 (s, 1H, 4), 8.78 (d, $^3J_{HH}$ = 4.8 Hz, 1H, pyrr), 8.76 (d, $^3J_{HH}$ = 4.8 Hz, 1H, pyrr), 8.634 (d, $^3J_{HH}$ = 4.9 Hz, 1H, pyrr), 8.634 (d, $^3J_{HH}$ = 4.9 Hz, 1H, pyrr), 8.42 (AA'XX', 2H, *o*-Tol), 8.40 (d, $^3J_{HH}$ = 4.9 Hz, 1H, pyrr), 8.37 (d, $^3J_{HH}$ = 4.9 Hz, 1H, pyrr), 7.63(AA'XX', 2H, *m*-Tol), 7.34 (s, 1H, 2), 7.39 (m, 4H, *o*-Tol and *m*-Tol), 7.31 (s, 2H, *m*-Mes), 7.10 (s, 1H, *m*-Mes), 6.96 (s, 1H, *m*-Mes), 2.64 (s, 6H, *p*-Mes), 2.64 (s, 6H, *p*-Tol), 2.56 (s, 3H, *m*-Mes), 2.54 (s, 3H, *m*-Mes), 2.52 (s, 3H, *m*-Mes), 2.50 (s, 3H, *m*-Mes), 1.89 (s, 1H, 2-OH), 1.09 (s, 3H, *o*-Mes), 0.97 (s, 3H, *o*-Mes).

**$^{13}$C NMR (150 MHz, CDCl$_3$, 300 K)** δ 168.7, 140.4, 139.6, 138.2, 138.0, 137.9, 137.8, 137.7, 137.6, 137.3, 136.7, 136.2, 136.3, 136.0, 135.9, 134.9, 134.1, 133.6, 129.9, 129.4, 129.1, 128.3, 128.2, 127.9, 127.7, 127.1, 125.6, 119.0, 118.8, 118.1, 82.9, 29.7, 22.7, 21.9, 21.5, 21.4, 21.3, 20.6, 20.0.

**UV-Vis (CH$_2$Cl$_2$) $\lambda_{max}$ [nm] (logε)** 436 (4.04), 522 (3.01), 563 (2.96).

**Compound 6**-*OCH$_3$*

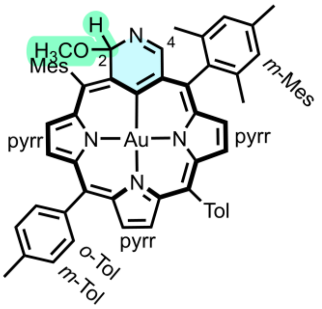

**6**-*OCH$_3$*

Compound **5** or **6** (10 mg, 8.0·10$^{-3}$ mmol) are converted into compound **6**-*OCH$_3$* on a chromatographic column packed with neutral Al$_2$O$_3$ using methanol as the eluent. Yield for **5** – 3.1 mg (40%) and for **6** mg – 7.1 mg (80%).

Compound **6**-*OCH$_3$* (5 mg, 5.2·10$^{-3}$ mmol) is converted into compound **6** on a chromatographic column packed with basic Al$_2$O$_3$ using DCM as the eluent. Yield 4.4 mg (90%).

**$^1$H NMR (500 MHz, CDCl$_3$, 300 K)** δ 9.26 (s, 1H, 2), 8.77 (d, $^3J_{HH}$ = 4.9 Hz, 1H, pyrr), 8.74 (d, $^3J_{HH}$ = 4.9 Hz, 1H, pyrr), 8.63 (d, $^3J_{HH}$ = 4.9 Hz, 1H, pyrr), 8.62 (d, $^3J_{HH}$ = 4.9 Hz, 1H, pyrr), 8.39 (d, $^3J_{HH}$ = 5.0 Hz, 1H, pyrr), 8.37 (d, $^3J_{HH}$ = 5.0 Hz, 1H, pyrr), 7.60 (brs, 4H, *o*-Tol), 7.41 (brs, 4H, *m*-Tol), 7.33 (s, 2H, *m*-Mes), 7.02 (s, 1H, *m*-Mes), 6.98(s, 1H, *m*-Mes), 6.89 (s, 1H, 2), 2.64 (s, 6H, *p*-Mes), 2.64 (s, 6H, *p*-Tol), 2.58 (s, 3H, *m*-Mes), 2.54 (s, 3H, *m*-Mes), 2.52 (s, 6H, *m*-Mes), 1.13 (s, 3H, *o*-Mes), 1.03 (s, 3H, *o*-Mes).

**$^{13}$C NMR (150 MHz, CDCl$_3$, 300 K)** δ 169.6, 140.6, 139.6, 138.2, 138.1, 137.8, 137.5, 137.4, 137.3, 136.8, 136.4, 135.9, 135.8, 134.9, 134.1, 129.8, 129.4, 129.2, 128.2, 128.1, 127.7, 127.1, 126.8, 125.5, 124.7, 122.4, 118.9, 118.5, 118.3, 113.4, 90.4, 56.8, 29.8, 22.6, 21.7, 21.3, 20.7, 20.5.

**HRMS (ESI)** *m/z* = 965.3613 calc. for [$C_{54}H_{46}N_4OAu$]$^+$, [M+H]$^+$: 965.3488.

**Synthesis of compound 7**

**C**ompound **3** (10 mg, 13.5·10$^{-3}$ mmol) and silver acetate (50 mg, 3.0·10$^{-1}$ mmol) were dissolved in a mixture of chloroform and acetonitrile (v:v 1:1). The mixture was refluxed for 1 hour under

nitrogen. Product **7** was subjected to chromatography ($SiO_2$, 10% EtOAc in DCM). Yield – 6.3 mg (50%).

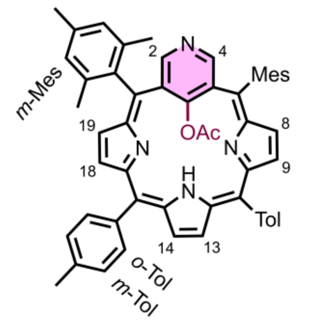


**7**

**$^1$H NMR (500 MHz, $CDCl_3$, 300 K)** δ 9.73 (brs, 1H, NH), 8.20 (s, 2H, 2, 4), 7.32 (AA'XX', 4H, *o*-Tol), 7.23 (AA'XX', 4H, *m*-Tol), 6.98 (brs, 2H, *m*-Mes), 6.91 (brs, 2H, *m*-Mes), 6.79 (s, 2H, 13, 14), 6.76 (d, $^3J_{HH}$ = 4.8 Hz, 2H, 8, 19), 6.47 (d, $^3J_{HH}$ = 4.8 Hz, 2H, 9, 18), 2.42 (s, 6H, Tol-$CH_3$), 2.35 (s, 6H, Mes-$CH_3$), 2.10 (s, 6H, Mes-$CH_3$), 1.96 (s, 6H, Mes-$CH_3$), 1.36 (s, 3H, OAc).

**$^{13}$C NMR (150 MHz, $CDCl_3$, 300 K)** δ 169.8, 166.4 (OAc), 159.0, 151.4 (2, 4), 148.5, 138.6, 138.5, 138.1, 137.3, 137.2, 136.5, 136.0, 135.5, 132.1, 131.4, 130.4, 128.6, 128.59, 127.9, 126.2, 125.6, 115.3, 21.3, 21.2, 21.1, 21.0, 20.7 (OAc).

**HRMS (ESI)** *m/z* = 797.3870 calc. for $[C_{52}H_{44}N_4OAg]^+$, $[M+H]^+$: 797.3850.

**UV-Vis ($CH_2Cl_2$) $\lambda_{max}$ [nm] (logε)** = 332 (3.10), 413 (3.35).

**Synthesis of compound 8**

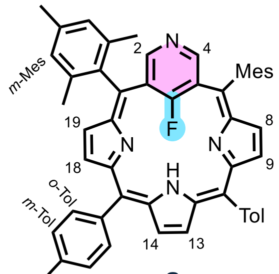


**8**

Compound **3** (5.0 mg, $6.8 \cdot 10^{-3}$ mmol) and AgF (25 mg, $2.0 \cdot 10^{-1}$ mmol) were dissolved in 3 ml pyridine. The mixture was refluxed for 1 h under argon atmosphere using a Schlenk line. Product **11** was subjected to chromatography ($SiO_2$, 10% EtOAc in DCM). Yield – 1.5 mg (30%).

**$^1$H NMR (500 MHz, $CDCl_3$, 300 K)** δ 10.20 (brs, 1H, NH), 8.13 (d, $^4J_{FH}$ = 8.3 Hz, 2H, 2, 4), 7.28 (AA'XX', 4H, *o*-Tol), 7.20 (AA'XX', 4H, *m*-Tol), 7.0 (brs, 2H, *m*-Mes), 6.85 (brs, 2H, *m*-Mes), 6.81 (d, $^3J_{HH}$ = 4.9 Hz, 2H, 8, 19), 6.64 (s, 2H, 13, 14), 6.42 (d, $^3J_{HH}$ = 4.9 Hz, 2H, 9, 18), 2.40 (brs, 6H, $CH_3$), 2.32 (brs, 6H, $CH_3$), 2.15 (brs, 18H, $CH_3$).

**$^1$H NMR (500 MHz, $CDCl_3$, 230 K)** δ 10.08 (brs, 1H, NH), 8.14 (d, $^4J_{FH}$ = 8.3 Hz, 2H, 2, 4), 7.28 (AA'XX', 4H, *o*-Tol), 7.20 (AA'XX', 4H, *m*-Tol), 7.01 (s, 2H, *m*-Mes), 6.83 (d, $^3J_{HH}$ = 4.9 Hz, 2H, 8, 19), 6.82 (s, 2H, *m*-Mes), 6.69 (s, 2H, 13, 14), 6.43 (d, $^3J_{HH}$ = 4.9 Hz, 2H, 9, 18), 2.39 (s, 6H, $CH_3$), 2.34 (s, 6H, $CH_3$), 2.30 (s, 6H, $CH_3$), 1.83 (s, 6H, $CH_3$).

**$^{13}$C NMR (125 MHz, $CDCl_3$, 300 K)** δ 171.4, 159.3, 148.9, 128.1, 127.3, 136.9, 136.4, 134.7, 132.1, 130.3, 128.7, 122.6, 114.9, 21.3, 21.1.

**HRMS (ESI)** *m/z* = 757.3732 calc. for $[C_{53}H_{46}N_4F]^+$, $[M+H]^+$: 757.3701.

**U-Vis ($CH_2Cl_2$) $\lambda_{max}$ [nm] (logε)** = 316 (3.54), 404 (3.63), 706 (3.11).

**Synthesis of compound 9**

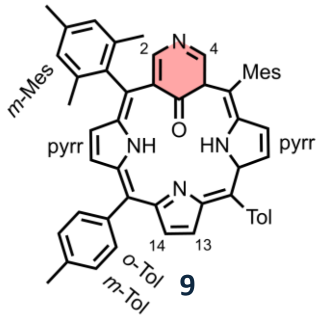

**9**

Compounds **7** (5 mg, 6.75·10$^{-3}$ mmol) or **8** (5.0 mg, 6.8·10$^{-3}$ mmol) were applied to a silicagel chromatography column (eluent: DCM/EtOAc, 95:5) and allowed to stand for 48 h. Both compounds underwent transformations to afford compound **9**. Yield for **7** – 1.1 mg, (20%) and for **8** – 4.3 mg (90%).

**$^1$H NMR (500 MHz, $CDCl_3$, 300 K)** δ 22.4 (brs, 1H, NH), 6.98 (AA'XX', 4H, *o*-Tol), 6.76 (AA'XX', 4H, *m*-Tol), 6.63 (s, 4H, *m*-Mes), 5.28 (s, 2H, 2, 4), 5.27 (s, 2H, 13, 14), 5.00 (d, $^3J_{HH}$ = 5.2 Hz, 1H, pyrr), 4.85 (d, $^3J_{HH}$ = 5.2 Hz, 1H, pyrr), 6.82 (s, 2H, *m*-Mes), 6.69 (s, 2H, 13, 14), 6.43 (d, $^3J_{HH}$ = 4.9 Hz, 2H, 9, 18), 2.28 (s, 12H, $CH_3$), 2.23 (s, 6H, $CH_3$), 2.13 (s, 6H, $CH_3$).

**$^{13}$C NMR – partial data based on HSQC and HMBC spectra** δ 172.5, 160.8, 154.5, 150.2, 148.9, 135.0, 134.5, 134.4, 133.8, 132.8, 131.4, 130.0, 137.1, 129.4, 127.8, 127.4, 117.8, 21.9, 21.2, 19.8.

**HRMS (ESI)** *m/z* = 755.3795 calc. for $[C_{53}H_{47}N_4O]^+$, $[M+H]^+$: 755.3744.

**Synthesis of compounds 8, 10 and 11**

Compound **3** (10 mg, 13.5·10$^{-3}$ mmol) and AgF (50 mg, 4.0·10$^{-1}$ mmol) were dissolved in 3 ml pyridine. The mixture was refluxed for 15 minutes. Products **8**, **10** and **11** were subjected to chromatography (TLC, $SiO_2$, 10% EtOAc (solution in $CH_2Cl_2$)). Yield – **11** (the first fraction) 2.5 mg (22%), **10** (the second fraction) 2.3 mg (20%), **8** (the third; <1%).

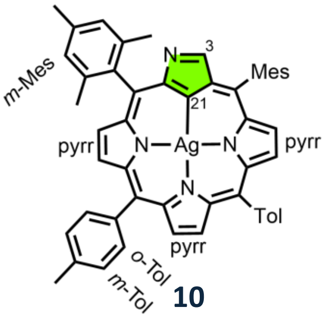

**10**

**$^1$H NMR (500 MHz, $C_6D_6$, 310 K)** δ 9.61 (s, 1H, 3), 8.82 (dd, $^3J_{HH}$ = 4.7 Hz, $^4J_{HAg}$ = 1.5 Hz, 1H, 18), 8.75 (brs, 2H, 7, 8), 8.74 (dd, $^3J_{HH}$ = 4.7 Hz, $^4J_{HAg}$ = 1.0 Hz, 1H, 17), 8.73 (d, $^3J_{HH}$ = 4.8 Hz, 1H, 12/13), 8.68 (d, $^3J_{HH}$ = 4.8 Hz, 1H, 12/13), 7.97 (AA'XX', 2H, *o*-Tol), 7.96 (AA'XX', 2H, *o*-Tol), 7.31 (d, 4H, *m*-Tol), 7.20 (s, 2H, 20-*m*-Mes),

7.08 (s, 2H, 5-*m*-Mes), 2.44 (s, 3H, 20-*p*-Mes), 2.41 (s, 3H, *p*-Tol), 2.39 (s, 3H, 5-*p*-Mes), 2.19 (s, 3H, 20-*o*-Mes-$CH_3$), 1.98 (s, 3H, 5-*o*-Mes-$CH_3$).

**$^{13}$C NMR (125 MHz, $CDCl_3$, 300 K)** δ 160.6 (3), 141.7, 140.8, 140.9, 139.8, 139.1, 138.6, 138.5, 138.4, 137.9, 137.7, 135.8, 133.8, 137.6 (1), 131.1, 130.0, 129.6, 128.0, 127.7, 121.8 (21), 29.7 ($CH_3$), 21.9 ($CH_3$), 21.6 ($CH_3$), 21.5 ($CH_3$), 21.5 ($CH_3$).

**HRMS (ESI)** *m/z* = 831.2612 calc. for $[C_{52}H_{44}N_4Ag]^+$, $[M+H]^+$: 831.2611.

**UV-Vis ($CH_2Cl_2$) $\lambda_{max}$ [nm] (logε)** = 447 (3.88), 520 (2.85), 638 (2.60).

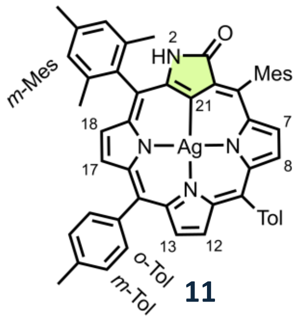


**11**

**$^1$H NMR (500 MHz, $CDCl_3$, 300 K)** δ 8.70 (dd, $^3J_{HH}$ =4.8 Hz, $^4J_{HAg}$ = 1.1 Hz, 1H, 17), 8.68 (dd, $^3J_{HH}$ =4.9 Hz, $^4J_{HAg}$ = 1.1 Hz, 1H, 8), 8.66 (d, $^3J_{HH}$ =4.7 Hz, 1H, 12/13), 8.62 (d, $^3J_{HH}$ =4.7 Hz, 1H, 12/13), 8.54 (dd, $^3J_{HH}$ =4.9 Hz, $^4J_{HAg}$ = 1.6 Hz, 1H, 7), 8.50 (dd, $^3J_{HH}$ =4.8 Hz, $^4J_{HAg}$ = 1.6 Hz, 1H, 18), 8.42 (s, 1H, 2 (NH)), 8.00 (AA’XX’, 2H, *o*-Tol), 7.99 (AA’XX’, 2H, *o*-Tol), 7.52 (d, 4H, *m*-Tol), 7.25 (s, 2H, 20-*m*-Mes), 7.22 (s, 2H, 5-*m*-Mes), 2.67 (s, 6H, *p*-Tol), 2.555 (s, 3H, *p*-Mes), 2.551 (s, 3H, *p*-Mes), 1.91 (s, 6H, 20-*o*-Mes-$CH_3$), 1.83 (s, 6H, 5-*o*-Mes-$CH_3$).

**$^{13}$C NMR (125 MHz, $CDCl_3$, 300 K)** δ 168.1 (3), 139.6, 138.9, 137.63, 137.6, 137.5, 136.3, 136.29, 133.7, 130.1, 129.7, 129.6, 128.7, 128.4, 128.2, 127.7, 127.1, 126.2 (1 or 4), 121.6 (1 or 4), 120.7, 115.6 (21), 21.7 (*p*-Tol), 21.6 (*p*-Tol), 21.5 (*p*-Mes), 21.3 (*p*-Mes), 21.2 (20-*o*-Mes-$CH_3$), 21.15 (5-*o*-Mes-$CH_3$).

**HRMS (ESI)** *m/z* = 847.2585 calc. for $[C_{52}H_{44}N_4OAg]^+$, $[M+H]^+$: 847.2561.

**UV-Vis ($CH_2Cl_2$) $\lambda_{max}$ [nm] (logε)** = 447 (4.21), 527 (3.02), 569 (2.97), 611 (2.48).

## Figures

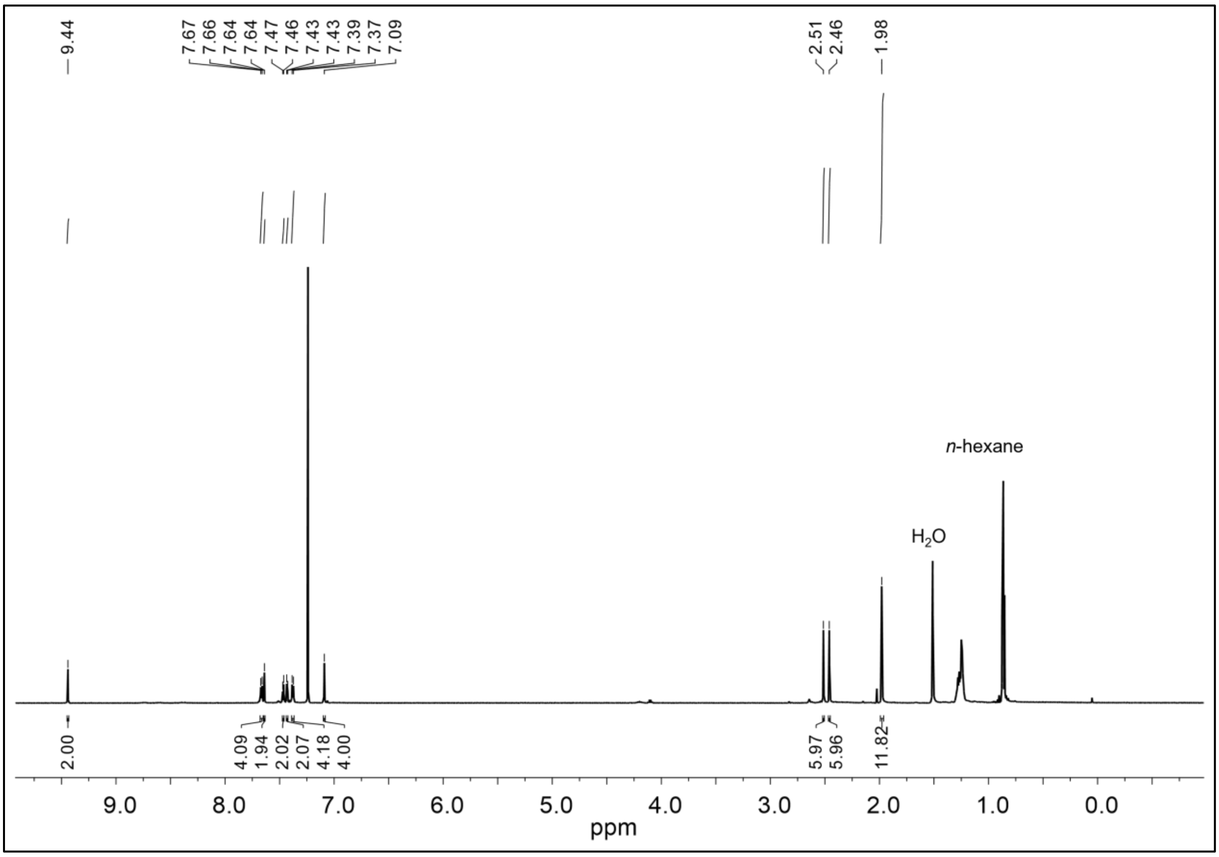


**Figure S1. $^1$H NMR spectrum of 4 (500 MHz, $CDCl_3$, 300 K).**

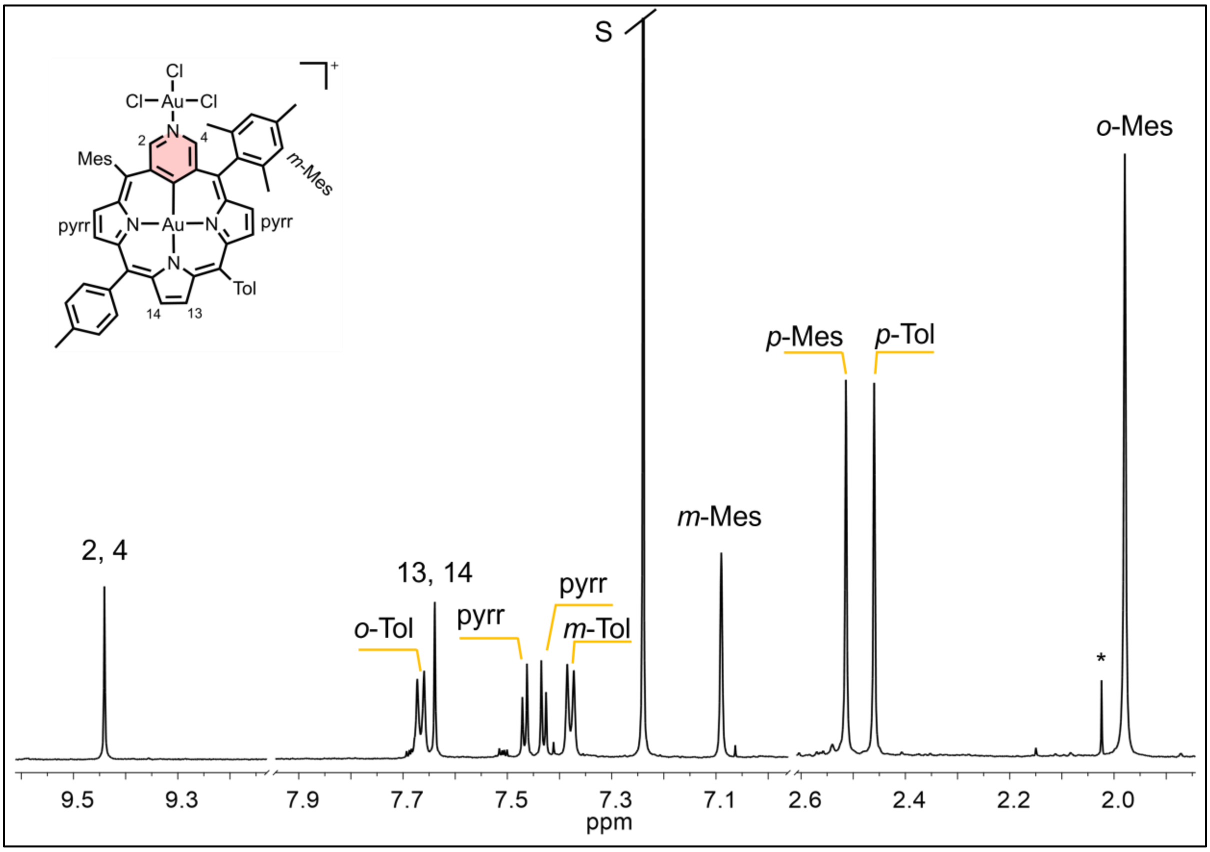


**Figure S2. Signals assignment; $^1$H NMR spectrum of 4; 500 MHz, $CDCl_3$, 300 K (selected ranges); * - impurities.**

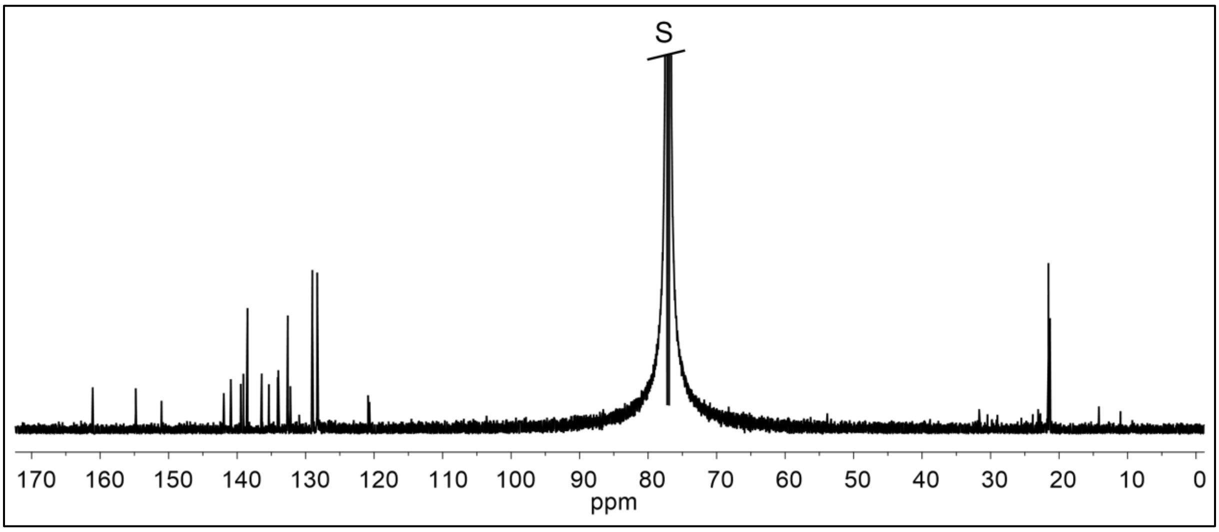

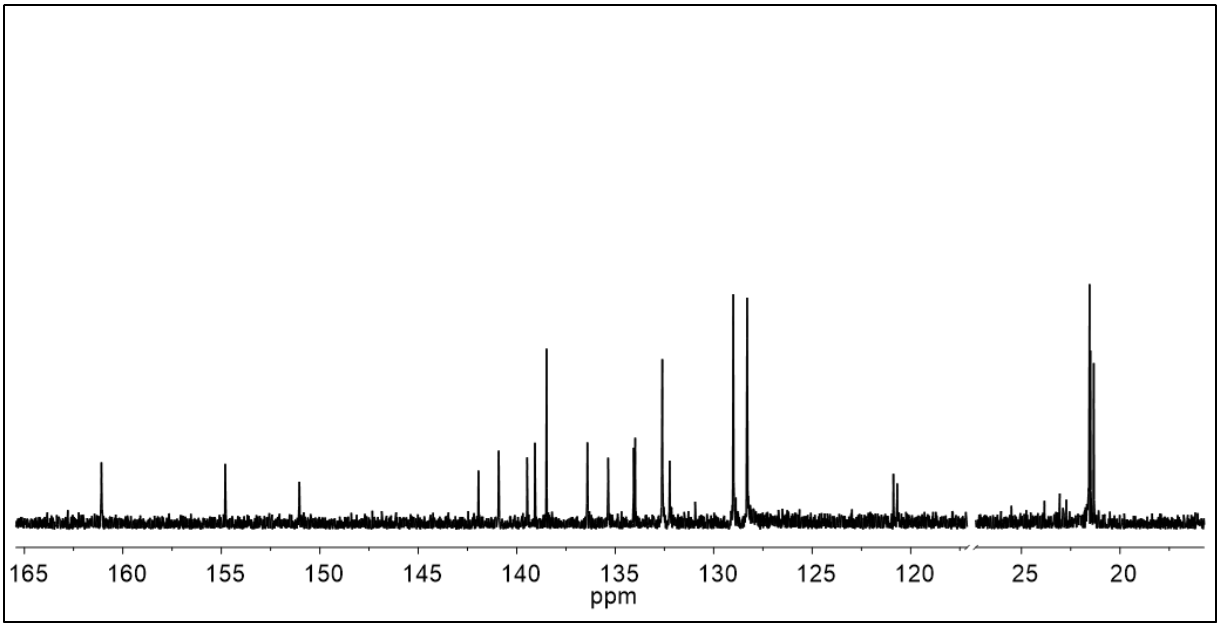


**Figure S3. $^{13}C$ NMR spectrum (125 MHz, $CDCl_3$, 300 K) of 4; (top: the whole spectral range, bottom: the most informative region).**

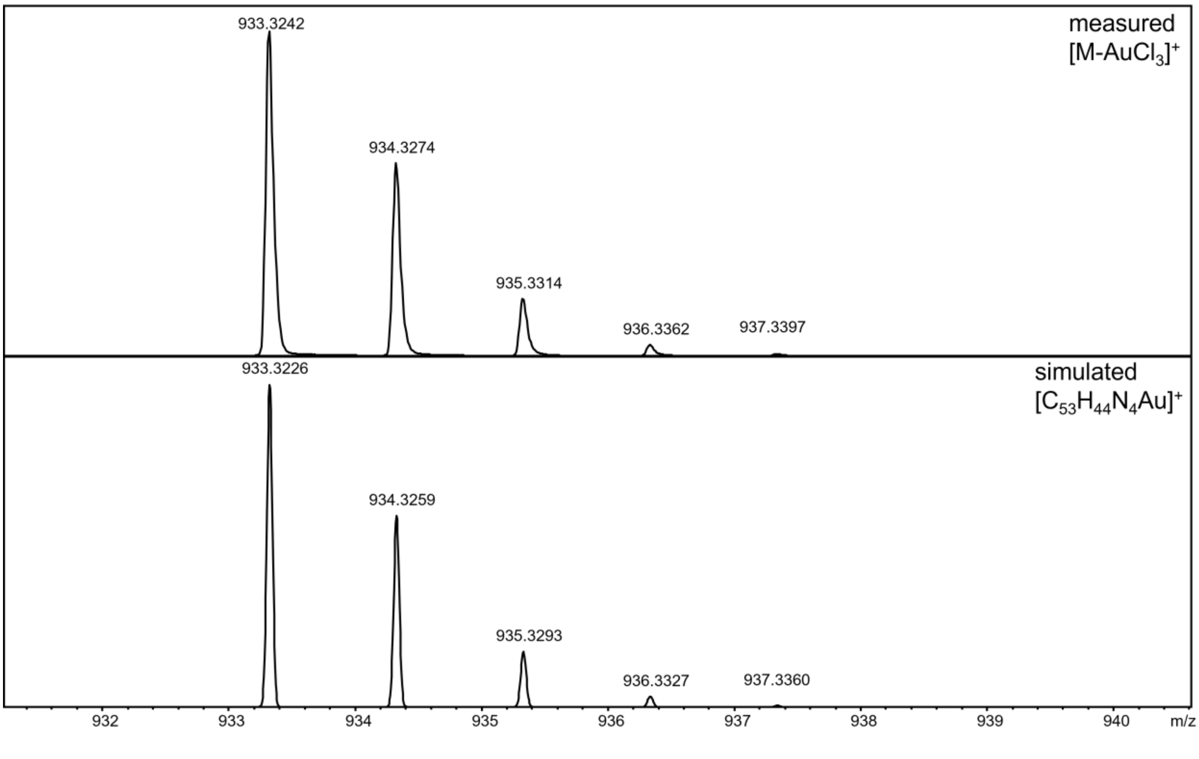


**Figure S4. Selected region of HRMS ESI (+MS) spectrum of 4.**

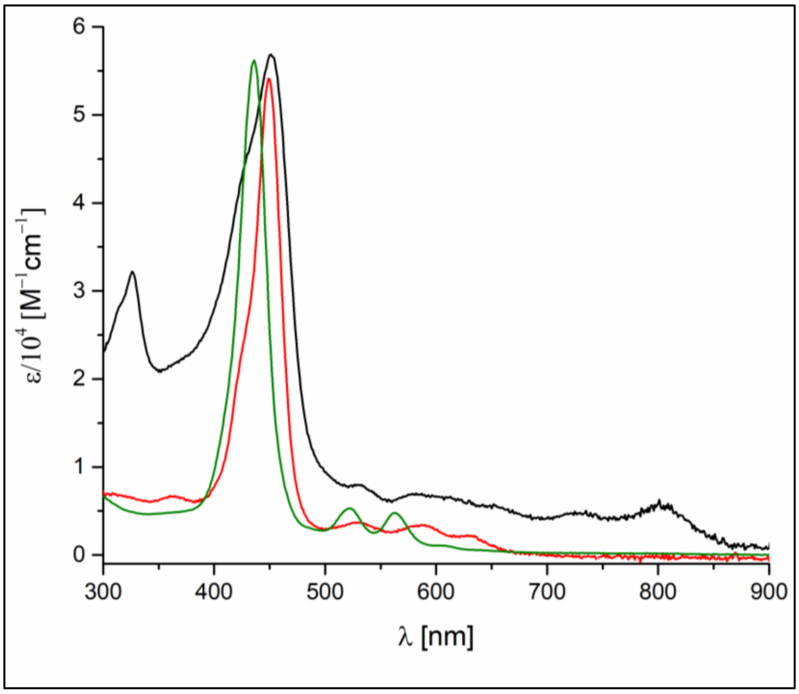


**Figure S5. UV-Vis spectra of 4 (black line), 5 (red line) and 6 (green line).**

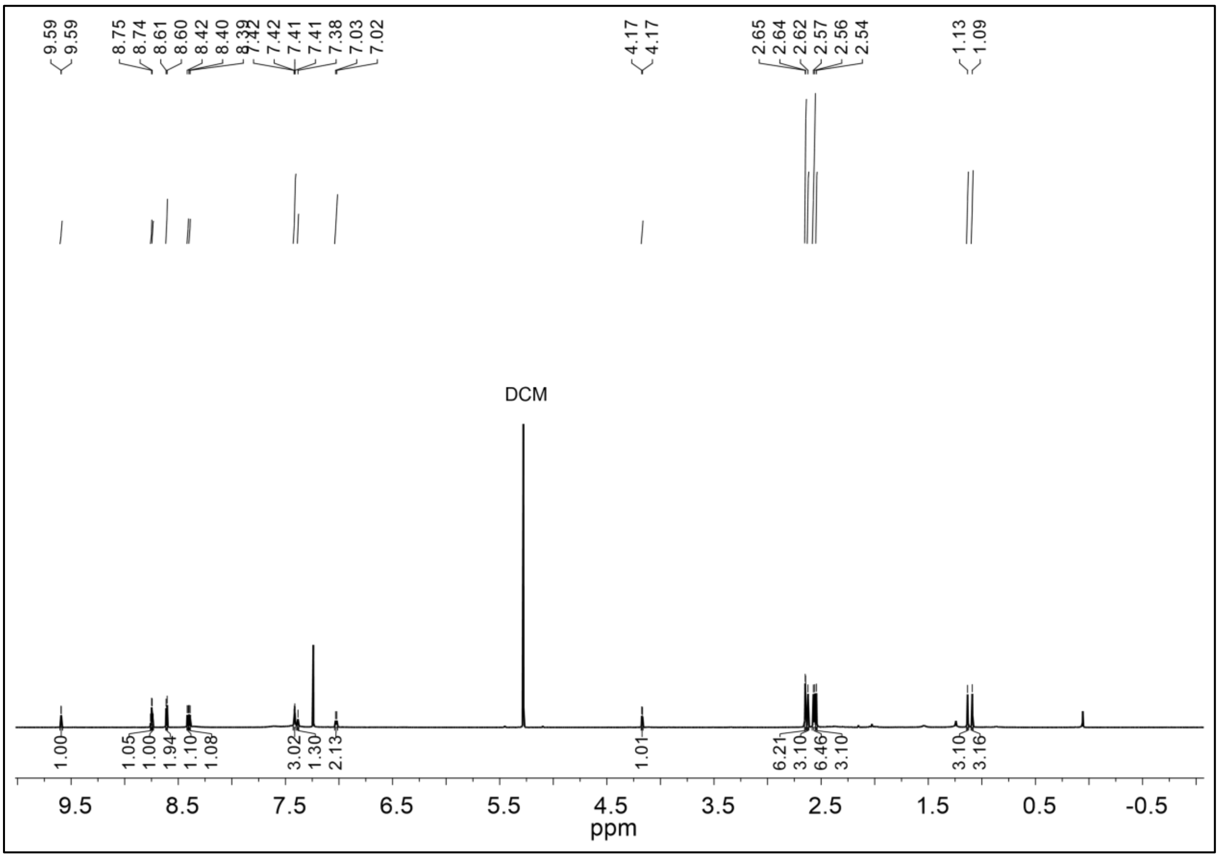


**Figure S6. $^1$H NMR spectrum of 5 (500 MHz, $CDCl_3$, 300 K).**

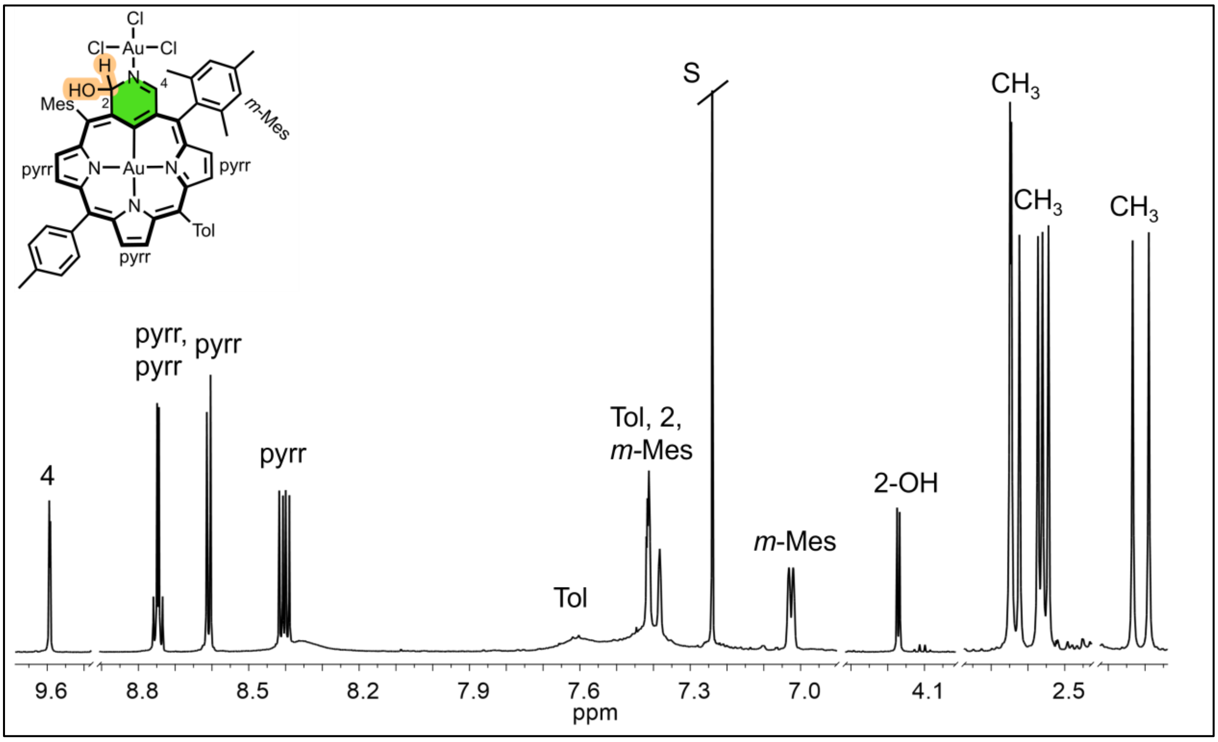


**Figure S7. Signals assignment; $^1$H NMR spectrum of 5; 500 MHz, $CDCl_3$, 300 K (selected ranges).**

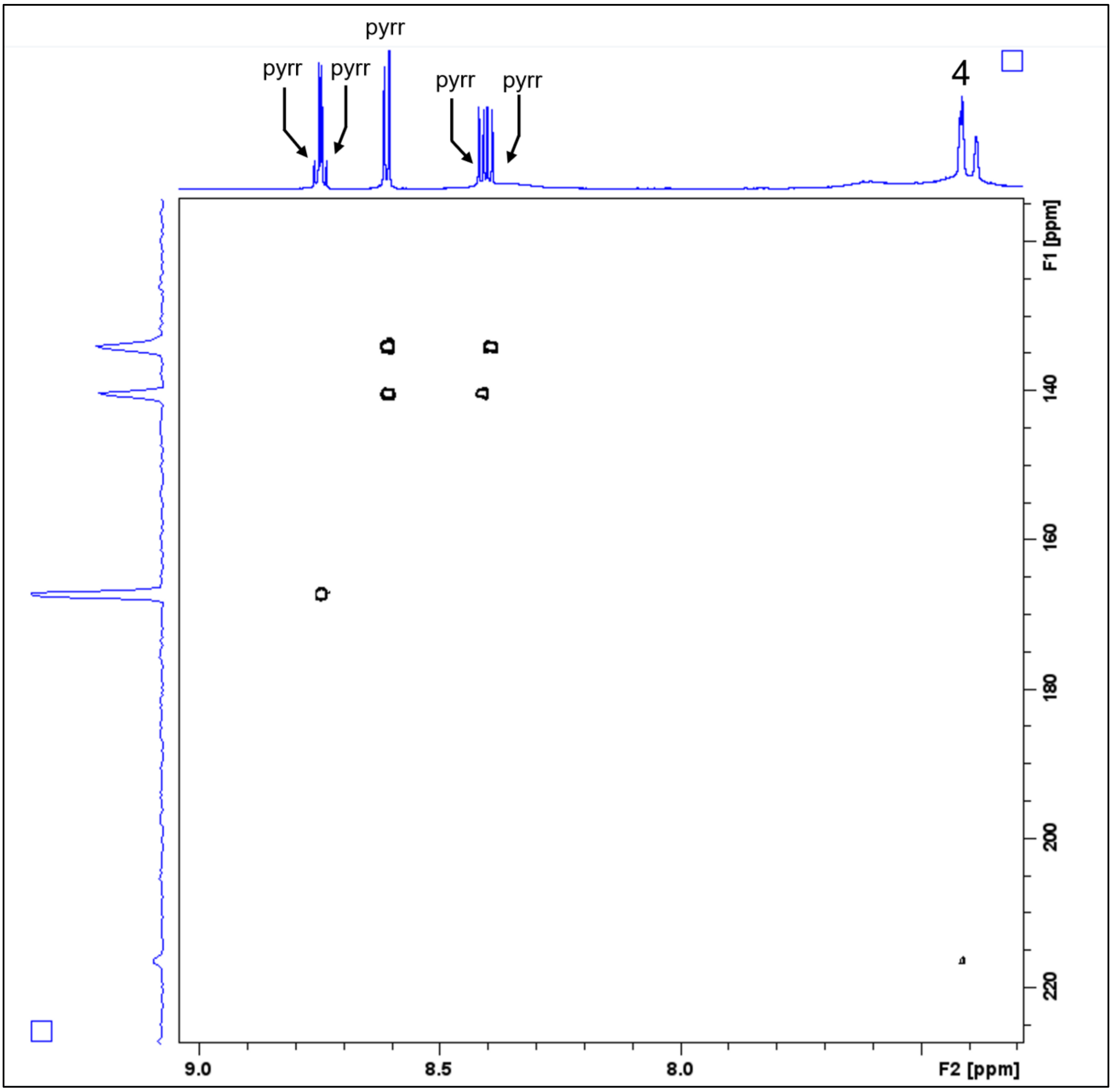


**Figure S8.** $^{1}H$-$^{15}N$ **HMBC spectrum (600 MHz, $CDCl_3$, 300 K) of 5.**

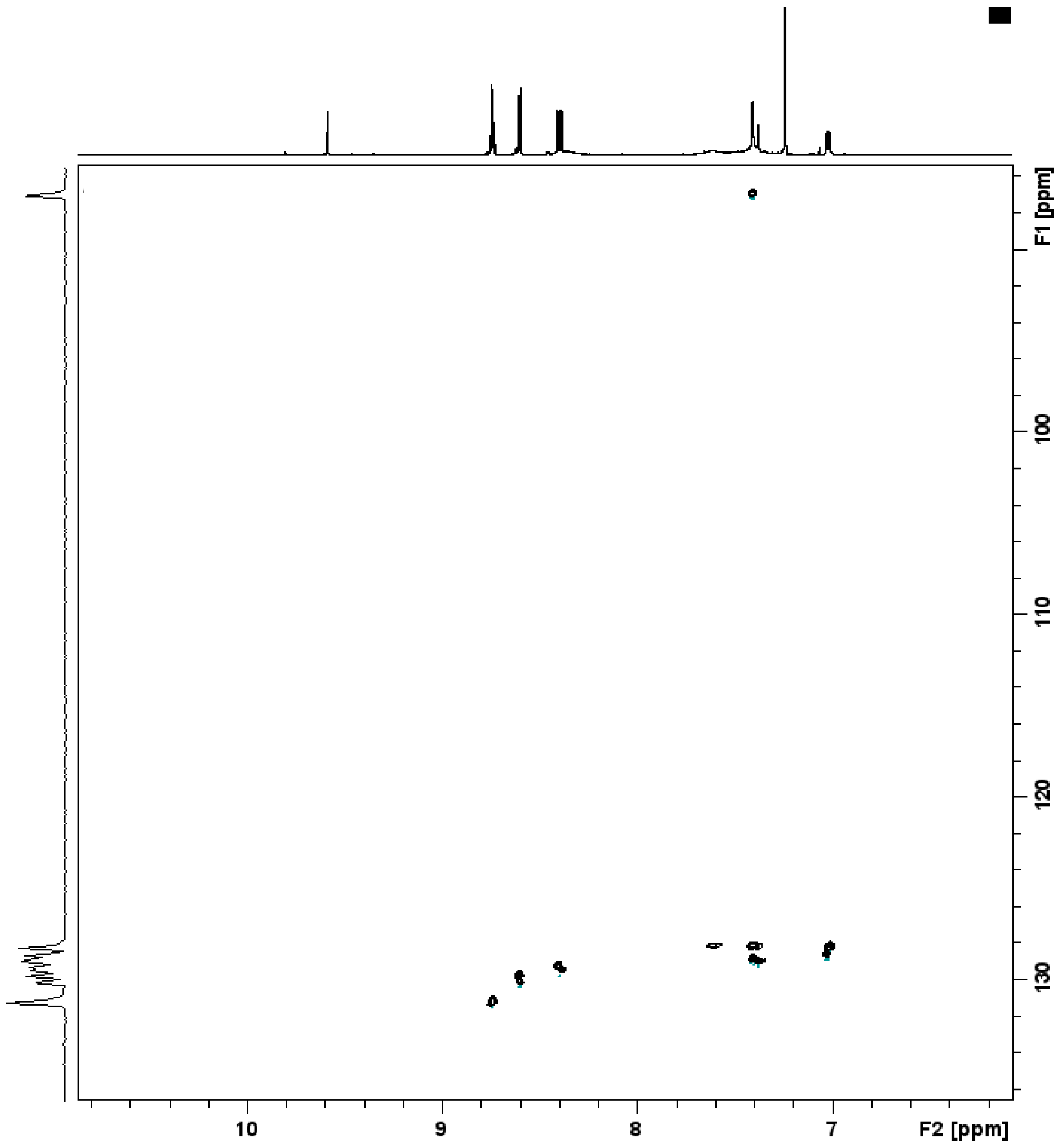


**Figure S9.** **$^{1}H$-$^{13}C$ HSQC spectrum (600 MHz, $CDCl_3$, 300 K) of 5 (selected range).**

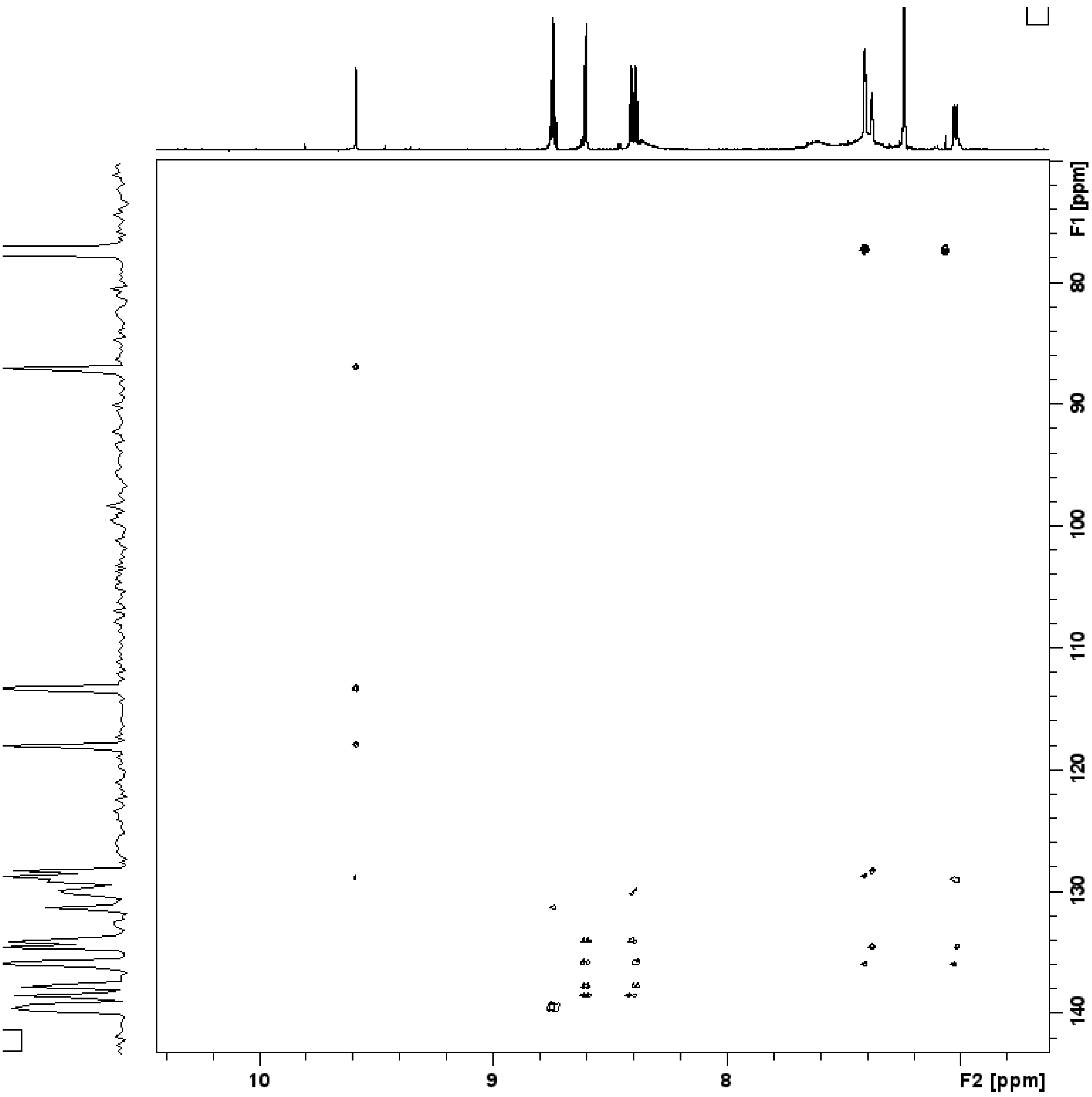


**Figure S10. $^{1}H$-$^{13}C$ HMBC spectrum (600 MHz, $CDCl_3$, 300 K) of 5 (selected range).**

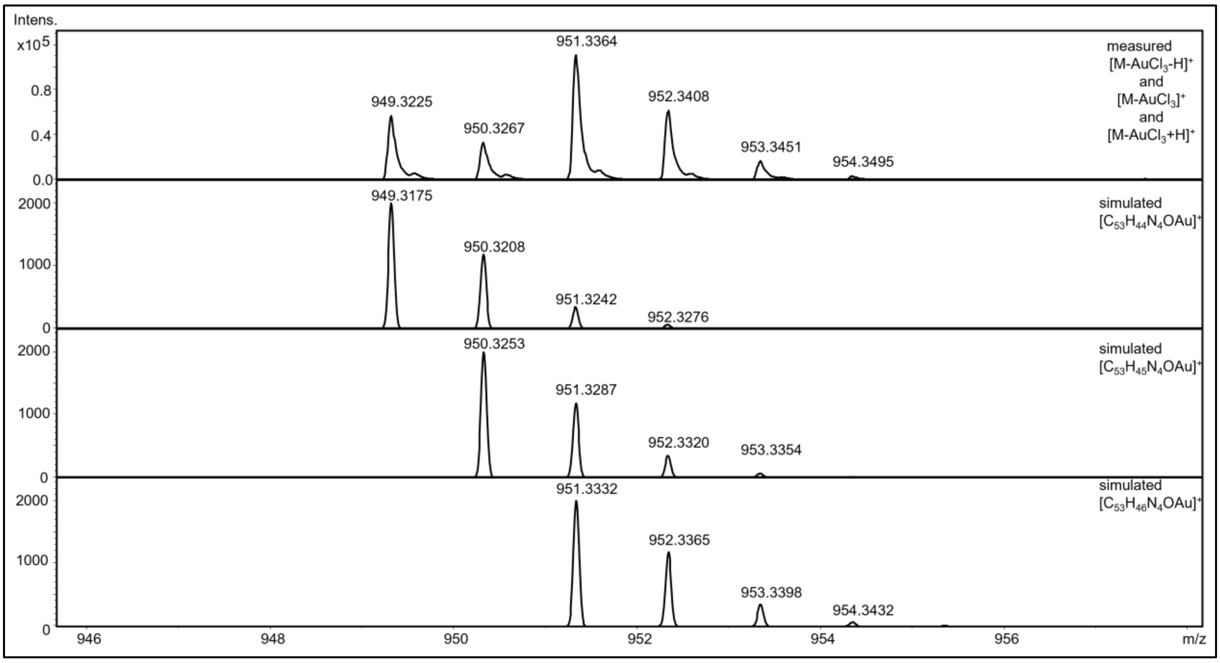


**Figure S11. Selected regions of HRMS ESI (+MS) spectrum of 5.**

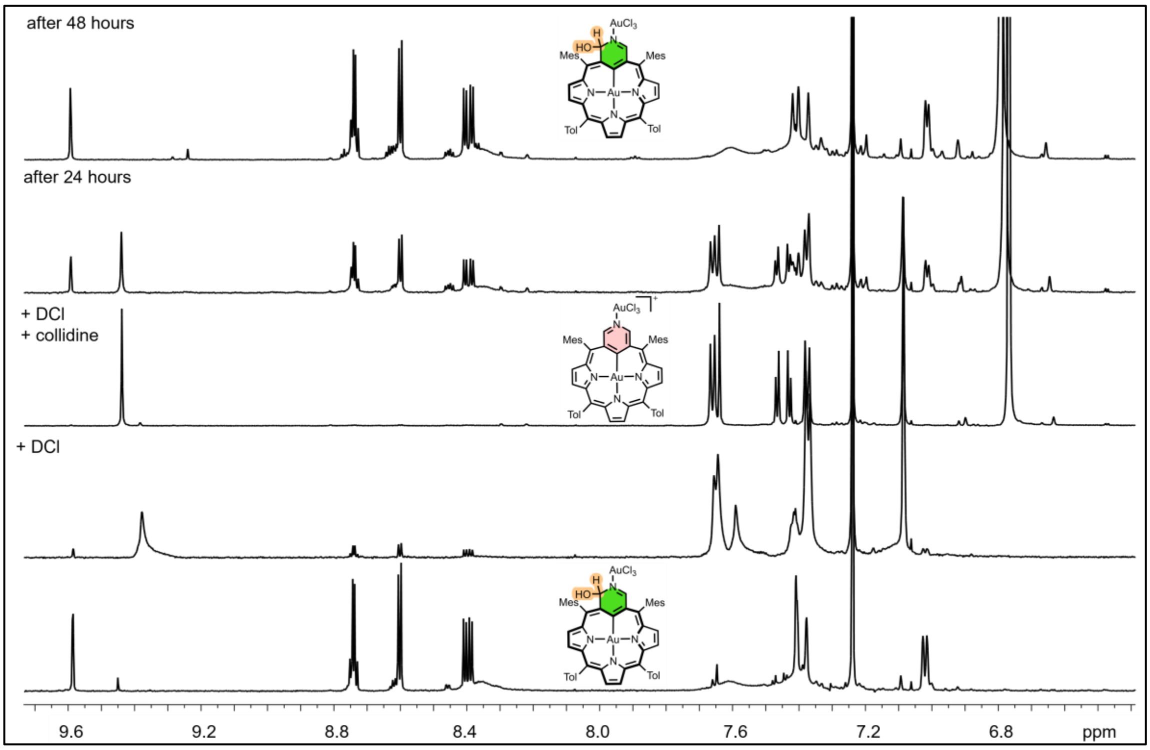


**Figure S12. Interconversion of compounds 5 and 4.**

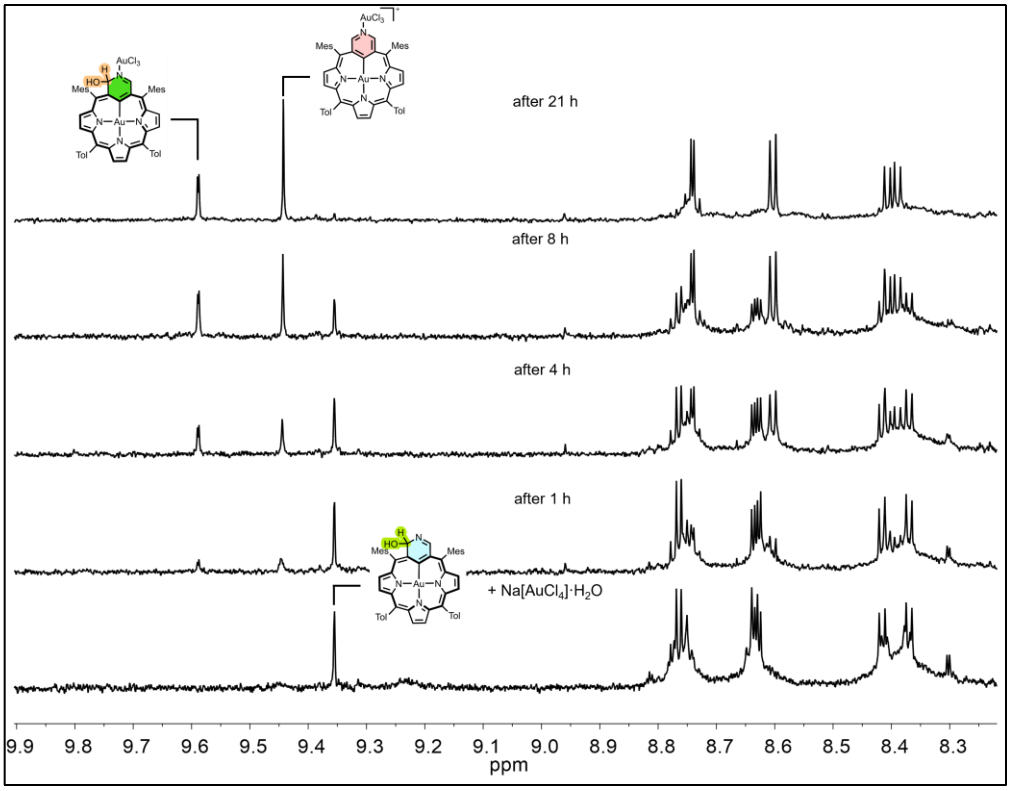


**Figure S13. Conversion of compound 6 into compounds 4 and 5 in the presence of gold ions.**

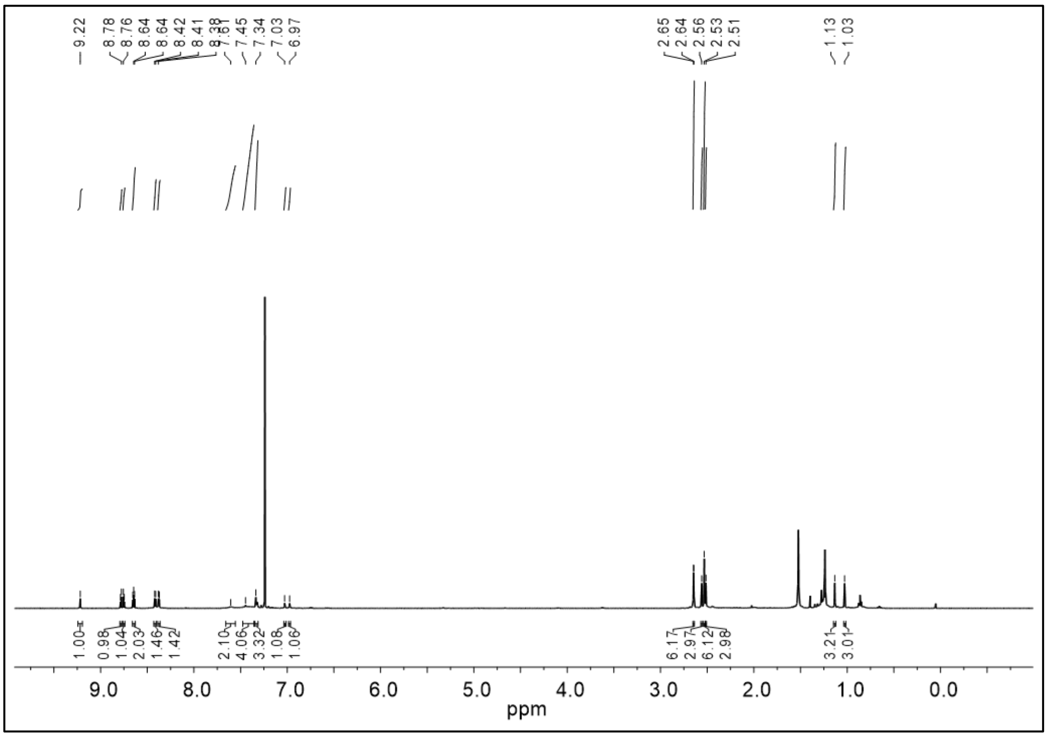


**Figure S14. $^{1}$H NMR spectrum of 6 (500 MHz, $CDCl_3$, 300 K).**

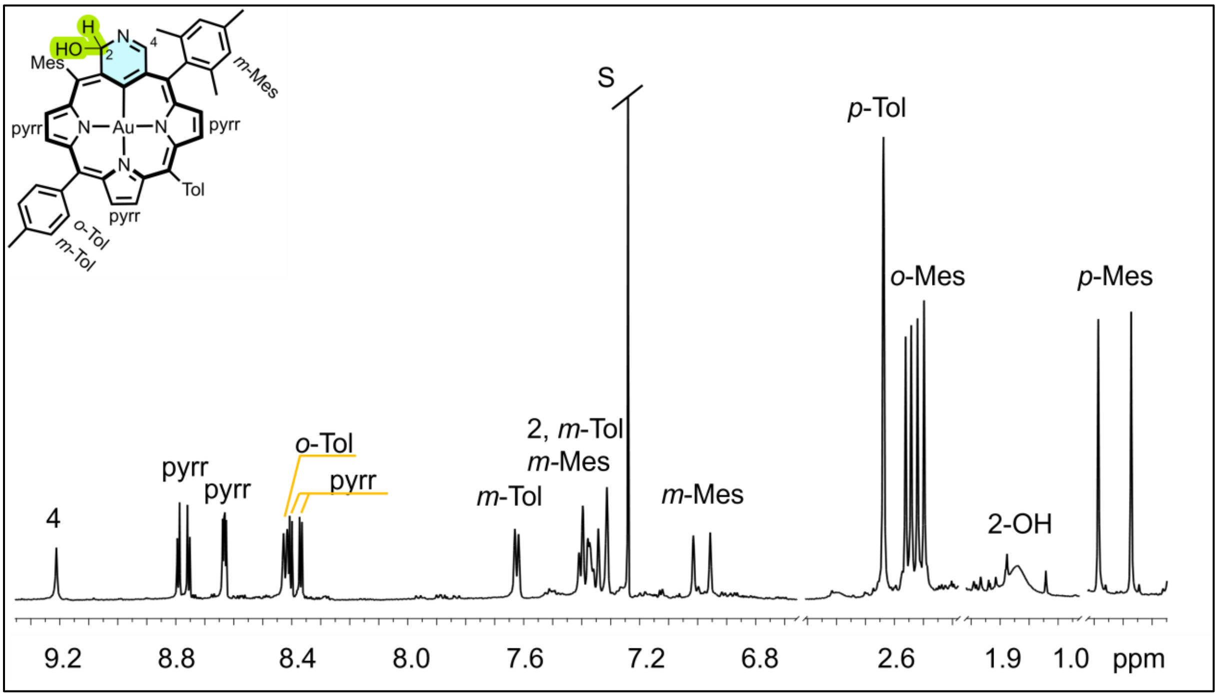


**Figure S15. Signals assignment; $^{1}H$ NMR spectrum of 6; 600 MHz, $CDCl_3$, 250 K (selected ranges).**

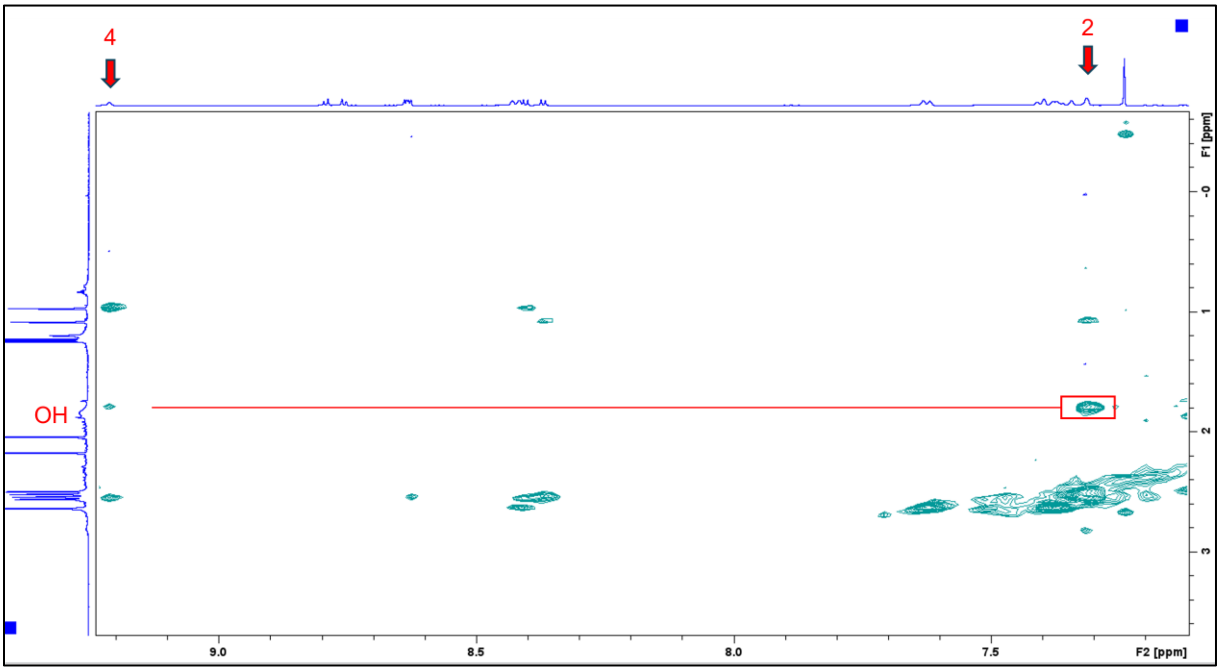


**Figure S16. $^{1}H$-$^{1}H$ NOESY spectrum (600 MHz, $CDCl_3$, 250 K) of 6 (selected range).**

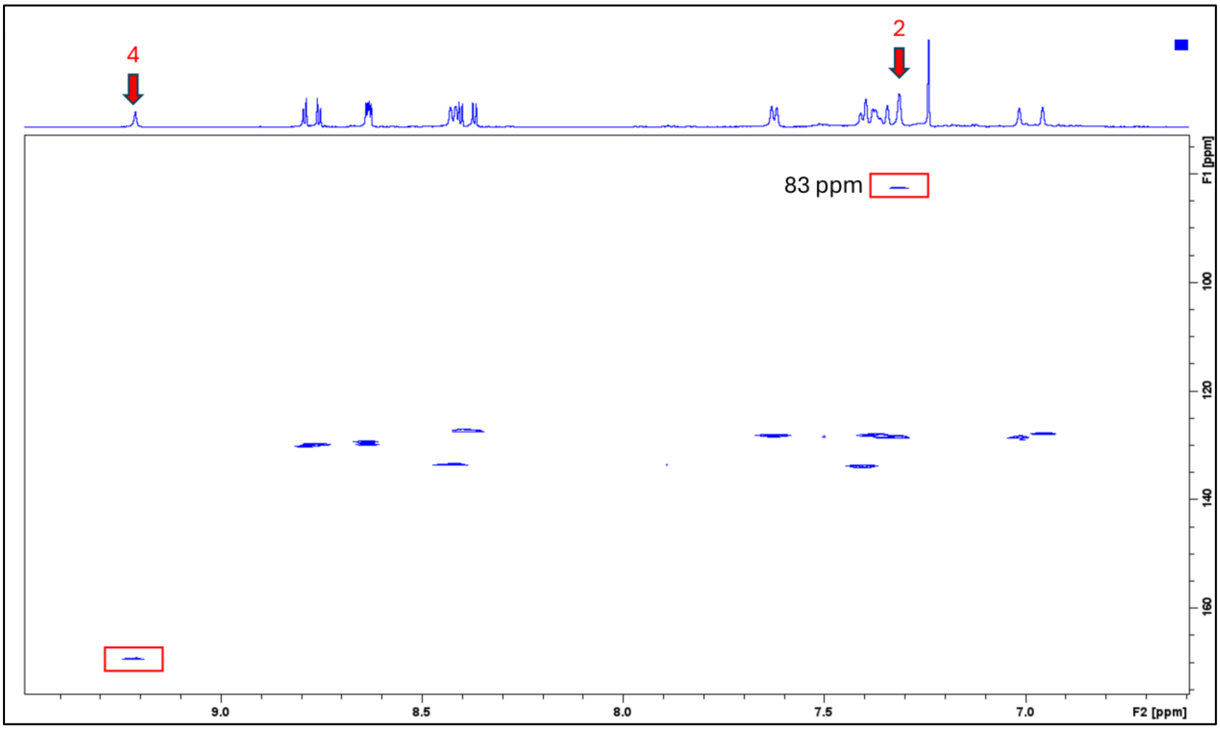


**Figure S17.** $^1H$-$^{13}C$ HSQC spectrum (600 MHz, $CDCl_3$, 250 K) of 6 (selected range).

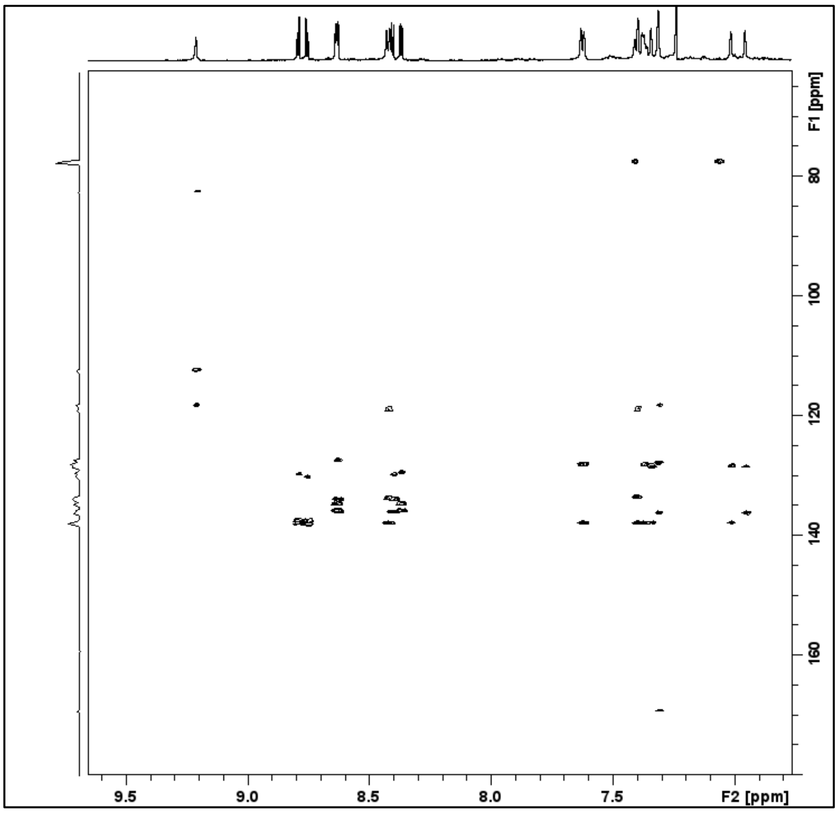


**Figure S18.** $^1H$-$^{13}C$ HMBC spectrum (600 MHz, $CDCl_3$, 250 K) of 6 (selected range).

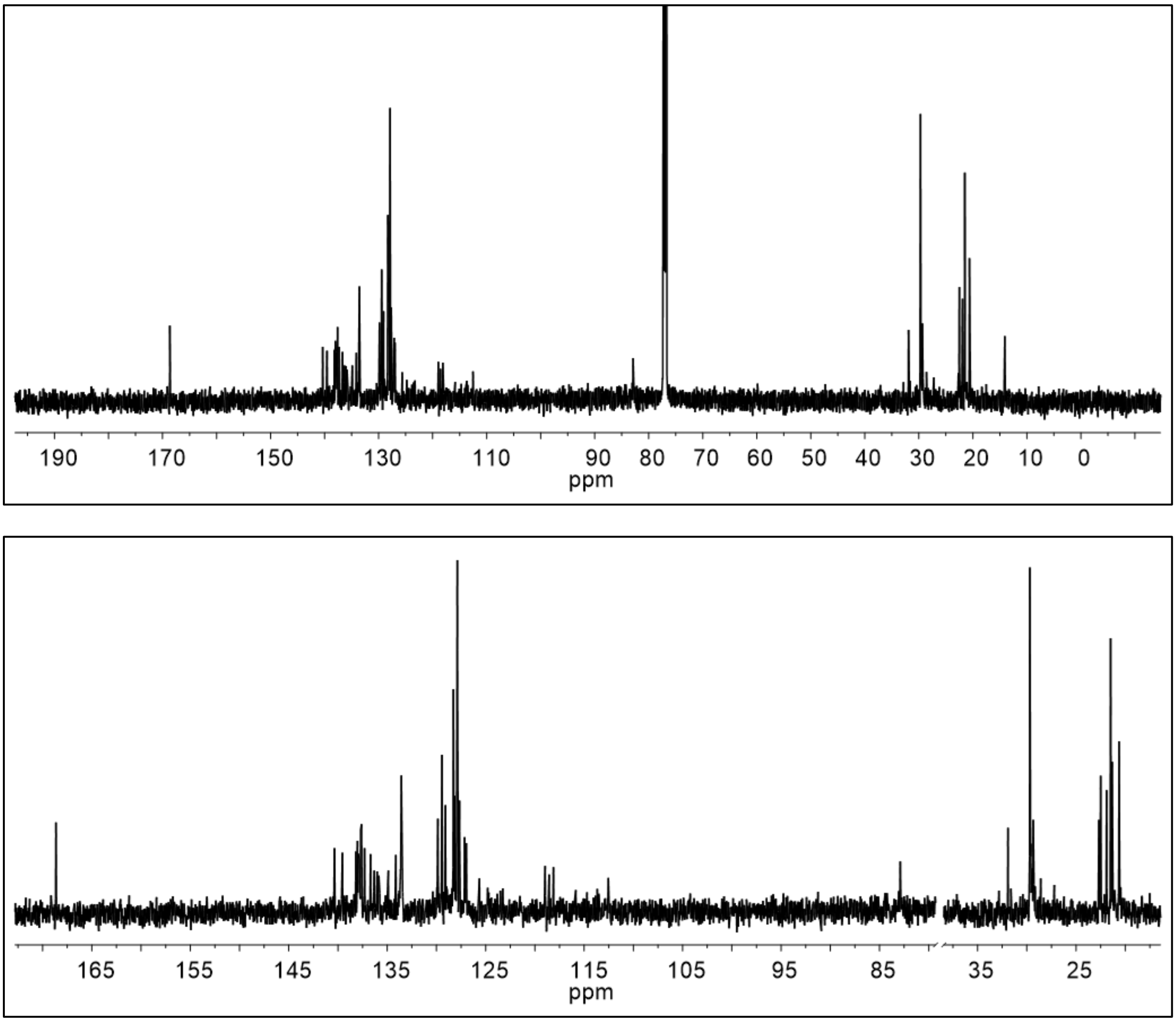


**Figure S19.** $^{13}$**C NMR spectrum (150 MHz, $CDCl_3$, 300 K) of 6; (top: the whole spectral range, bottom: the most informative region).**

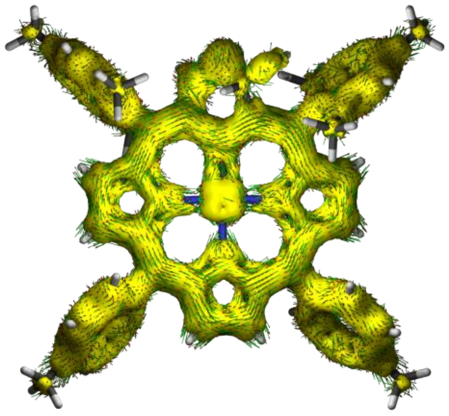

**Figure S20. AICD plot for 6.**

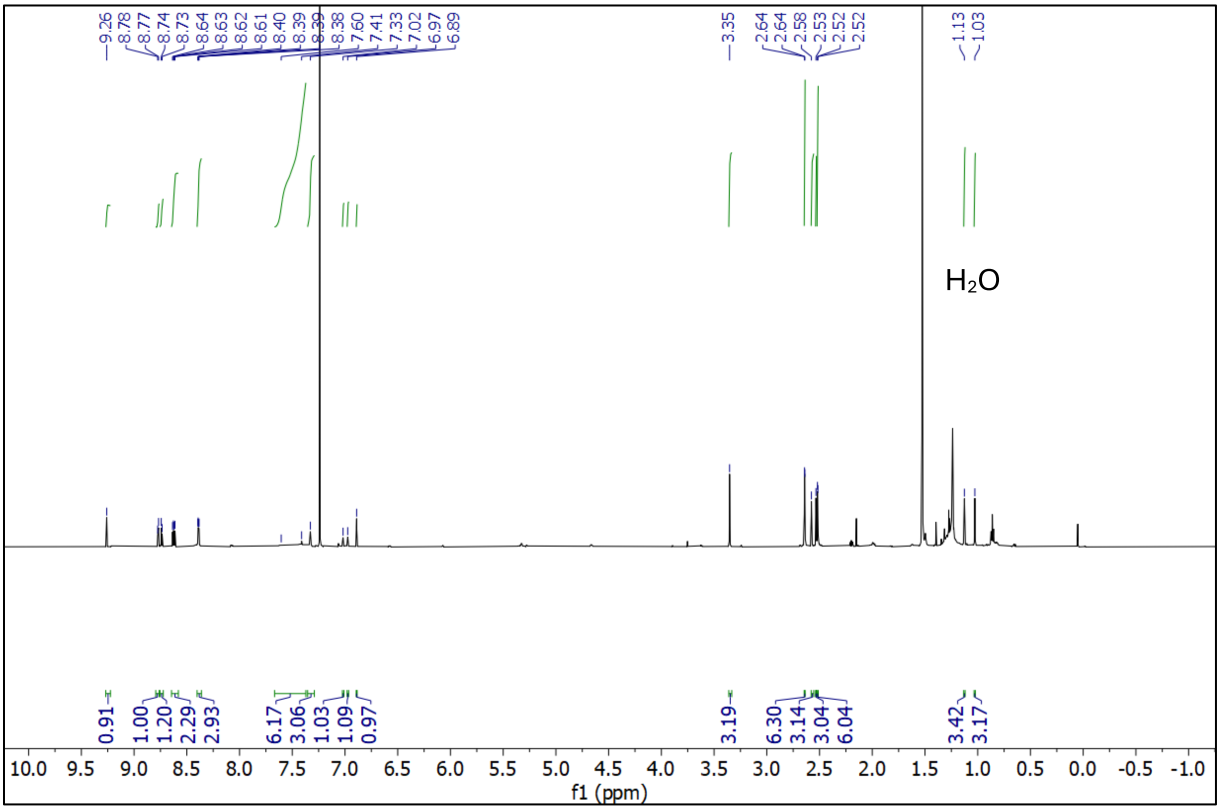


**Figure S21.** **$^1$H NMR spectrum of 6-OMe (600 MHz, $CDCl_3$, 300 K).**

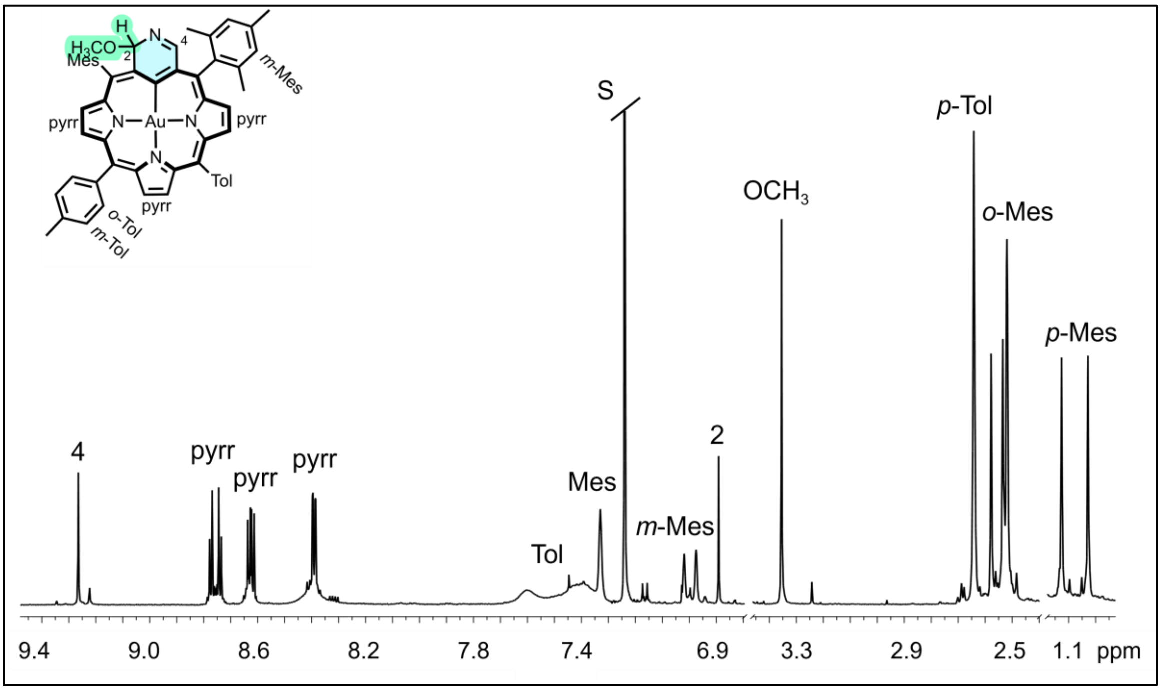


**Figure S22. Signals assignment; $^1$H NMR spectrum of 6-*$OCH_3$*; 500 MHz, $CDCl_3$, 300 K (selected ranges).**

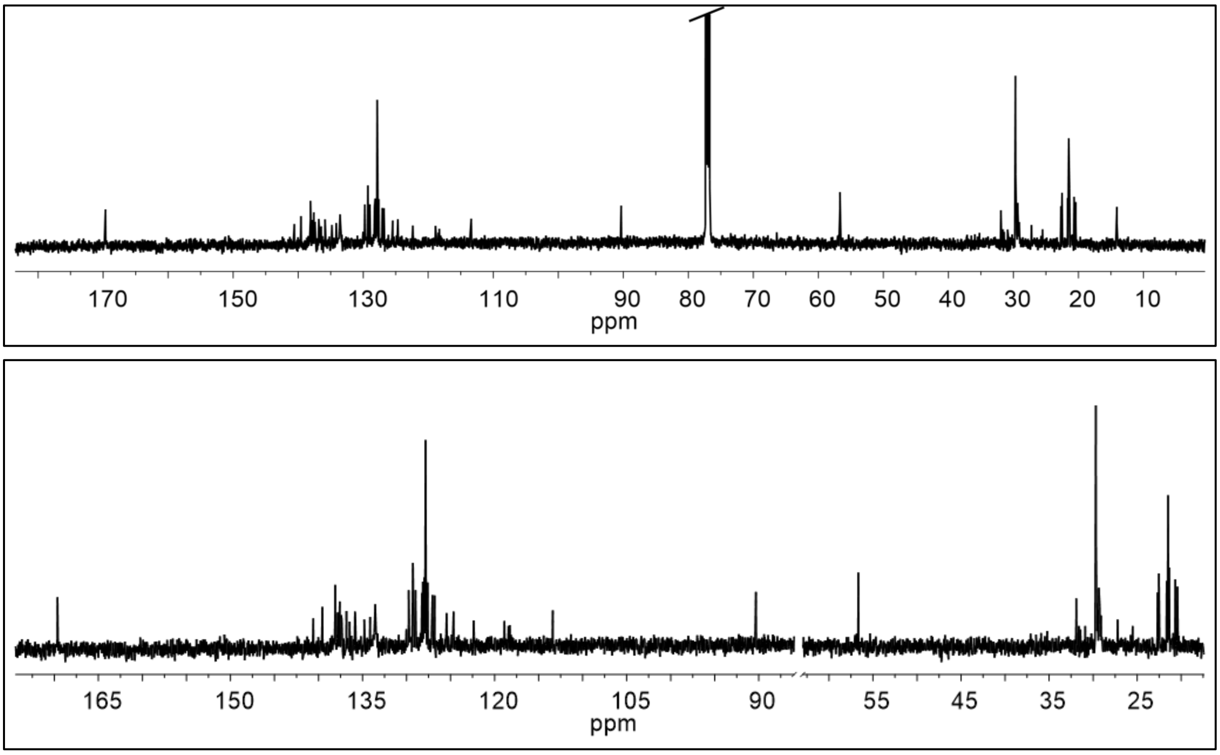


**Figure S23.** $^{13}$**C NMR spectrum (150 MHz, CDCl$_3$, 300 K) of 6-OCH$_3$; (top: the whole spectral range, bottom: the most informative region).**

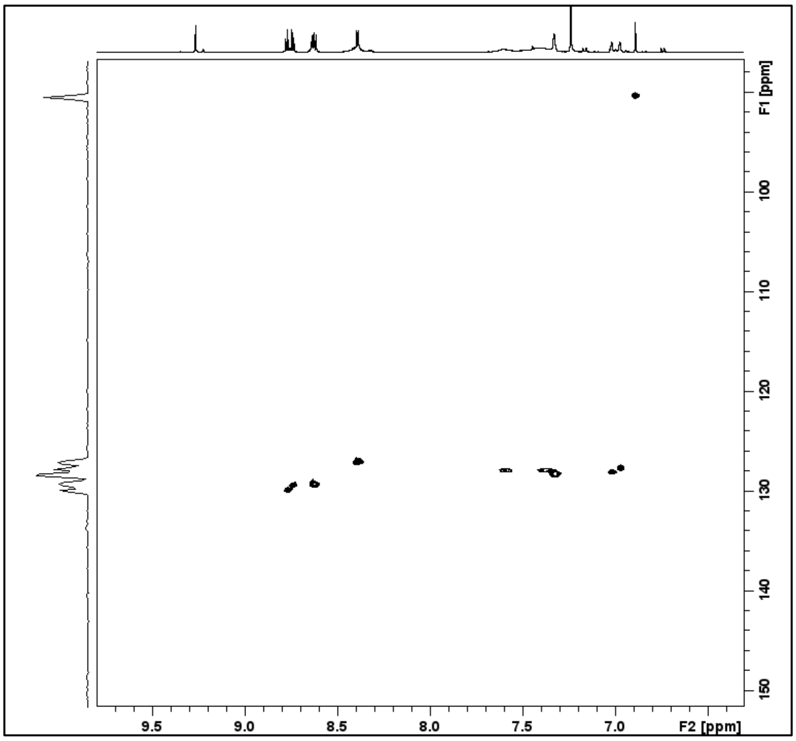


**Figure S24.** $^{1}$**H-**$^{13}$**C HSQC spectrum (600 MHz, CDCl$_3$, 300 K) of 6-*OCH$_3$* (selected range).**

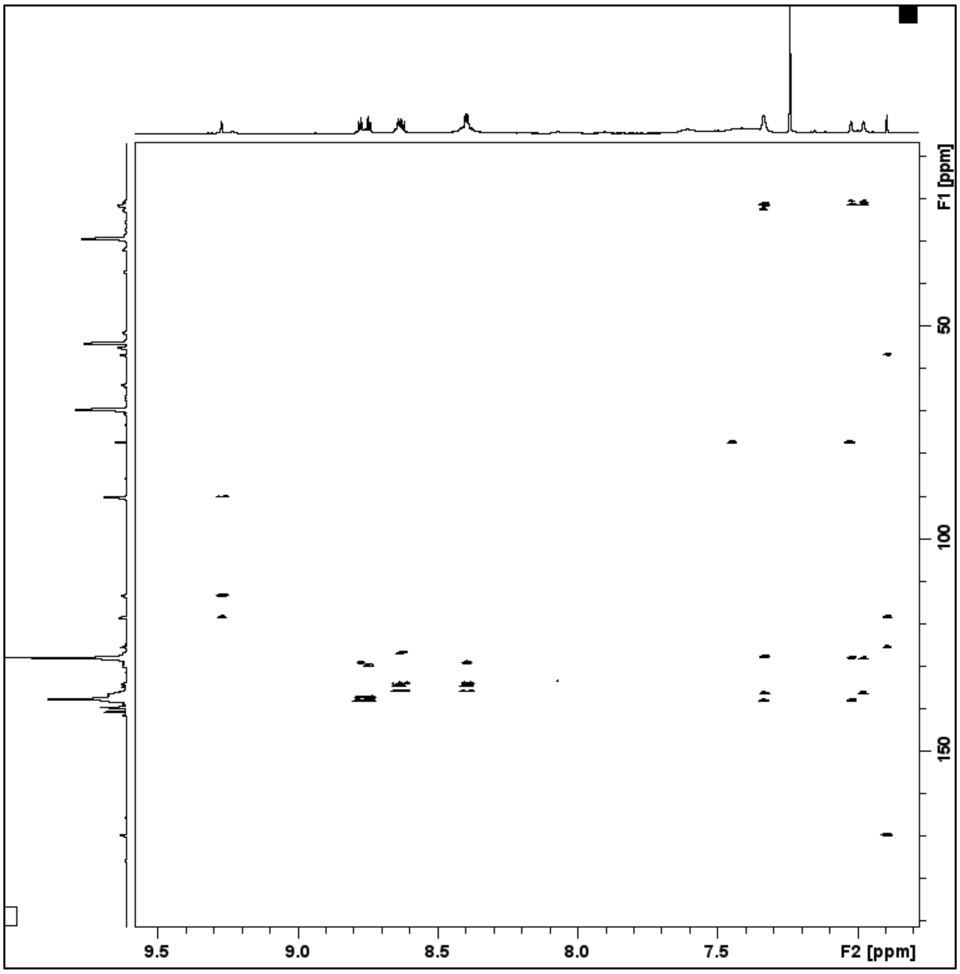


**Figure S25.** $^1H$-$^{13}C$ **HSQC spectrum (600 MHz,** $CDCl_3$**, 300 K) of 6-*OCH₃* (selected range).**

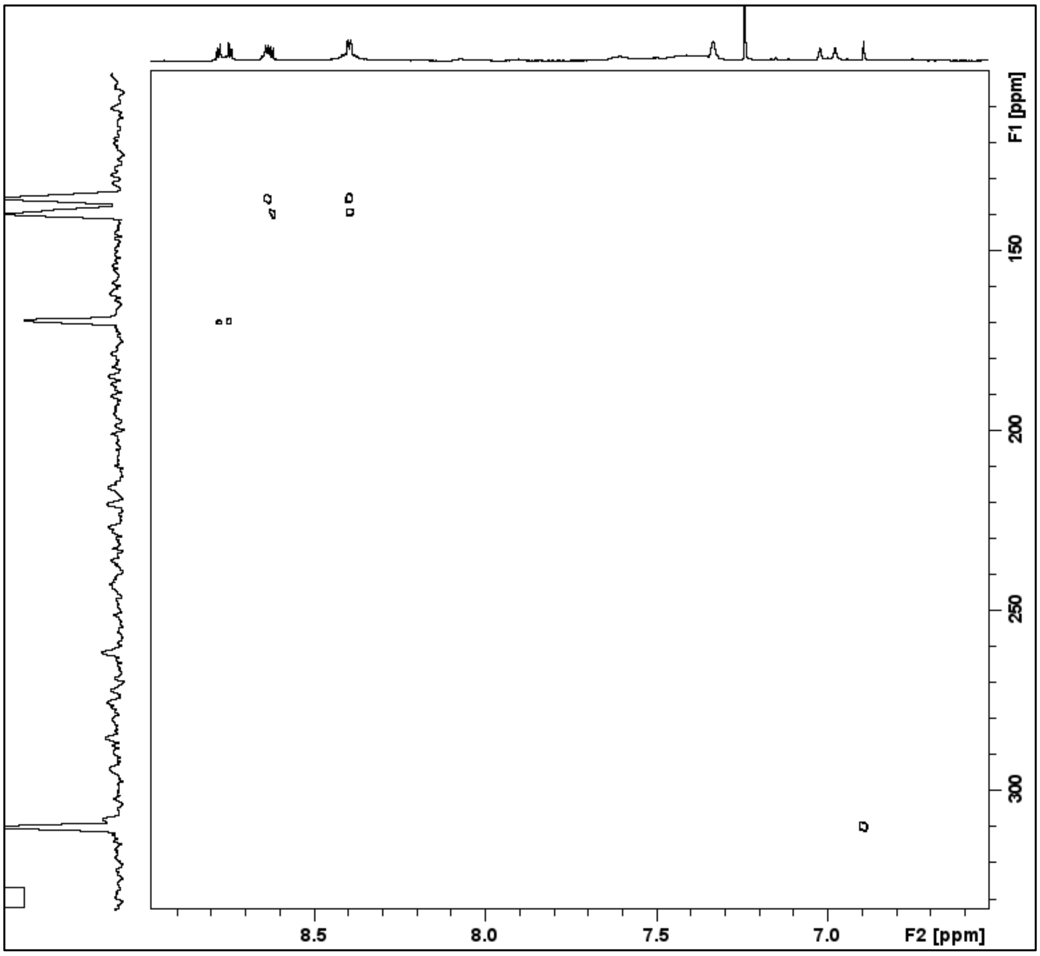


**Figure S26. $^{1}H$-$^{15}N$ HMBC spectrum (600 MHz, $CDCl_3$, 300 K) of 6-*$OCH_3$* (selected range).**

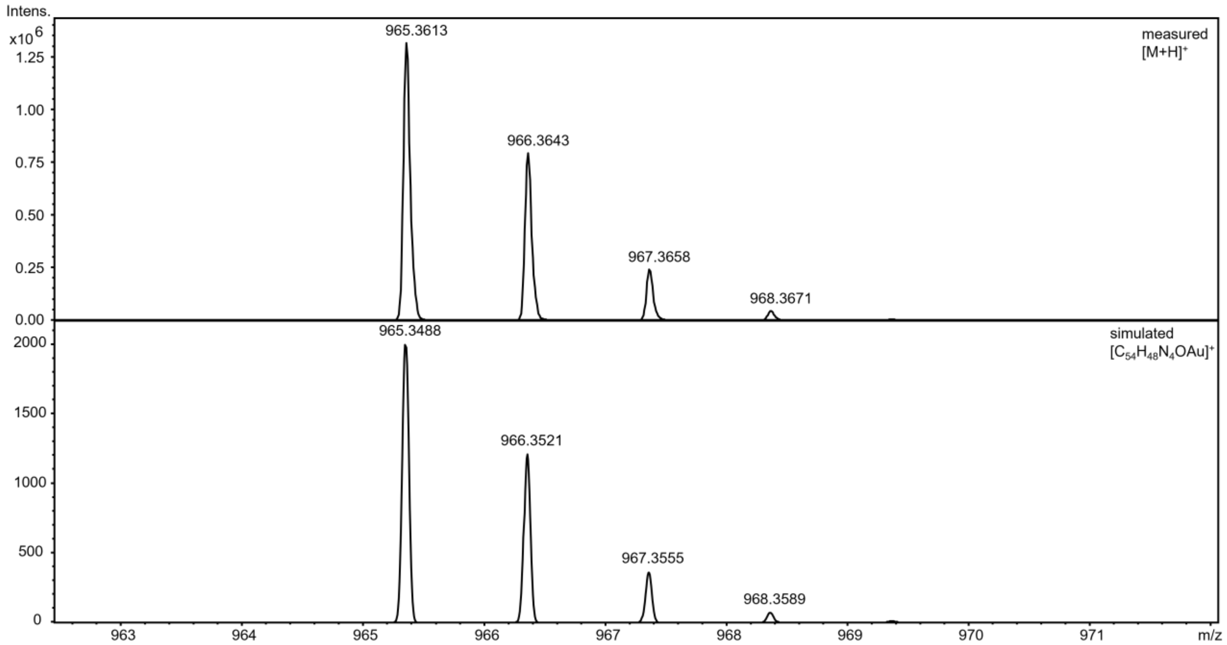


**Figure S27. Selected region of HRMS ESI (+MS) spectrum of 6-*$OCH_3$*.**

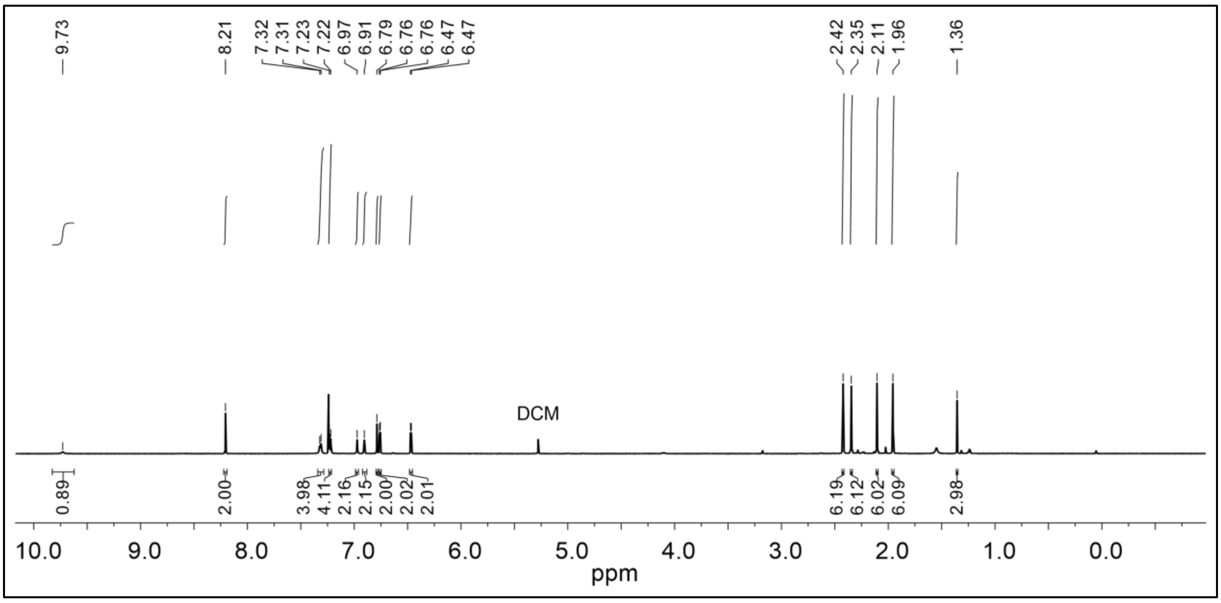


**Figure S28. $^{1}$H NMR spectrum of 7 (600 MHz, $CDCl_3$, 300 K).**

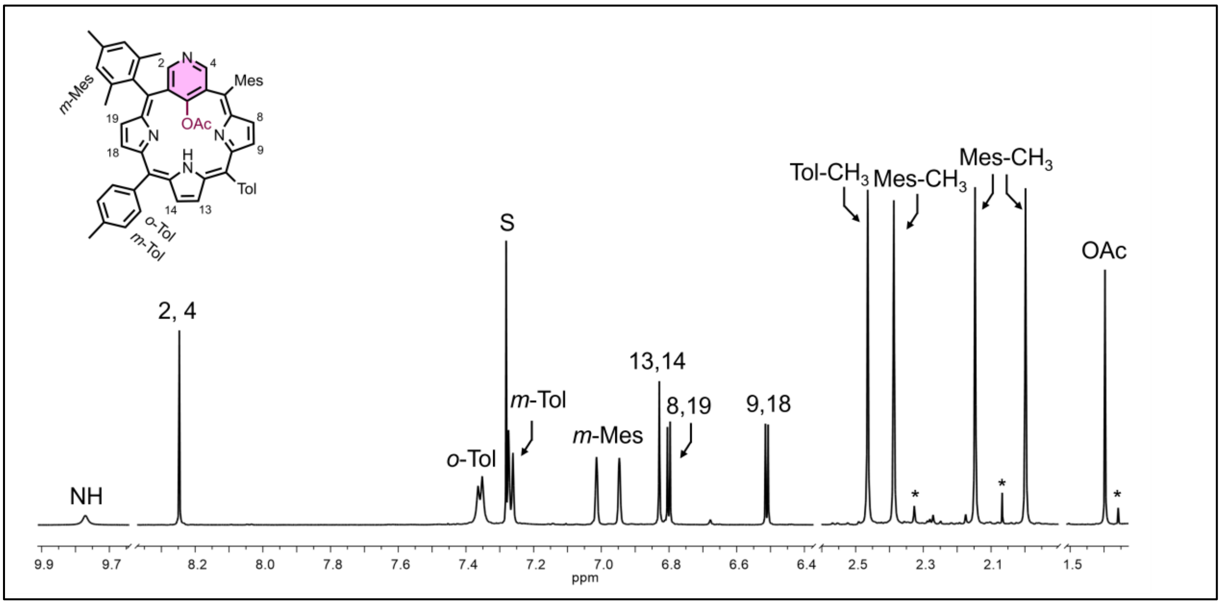


**Figure S29. Signals assignment; $^{1}$H NMR spectrum of 7; 600 MHz, $CDCl_3$, 300 K (selected ranges).**

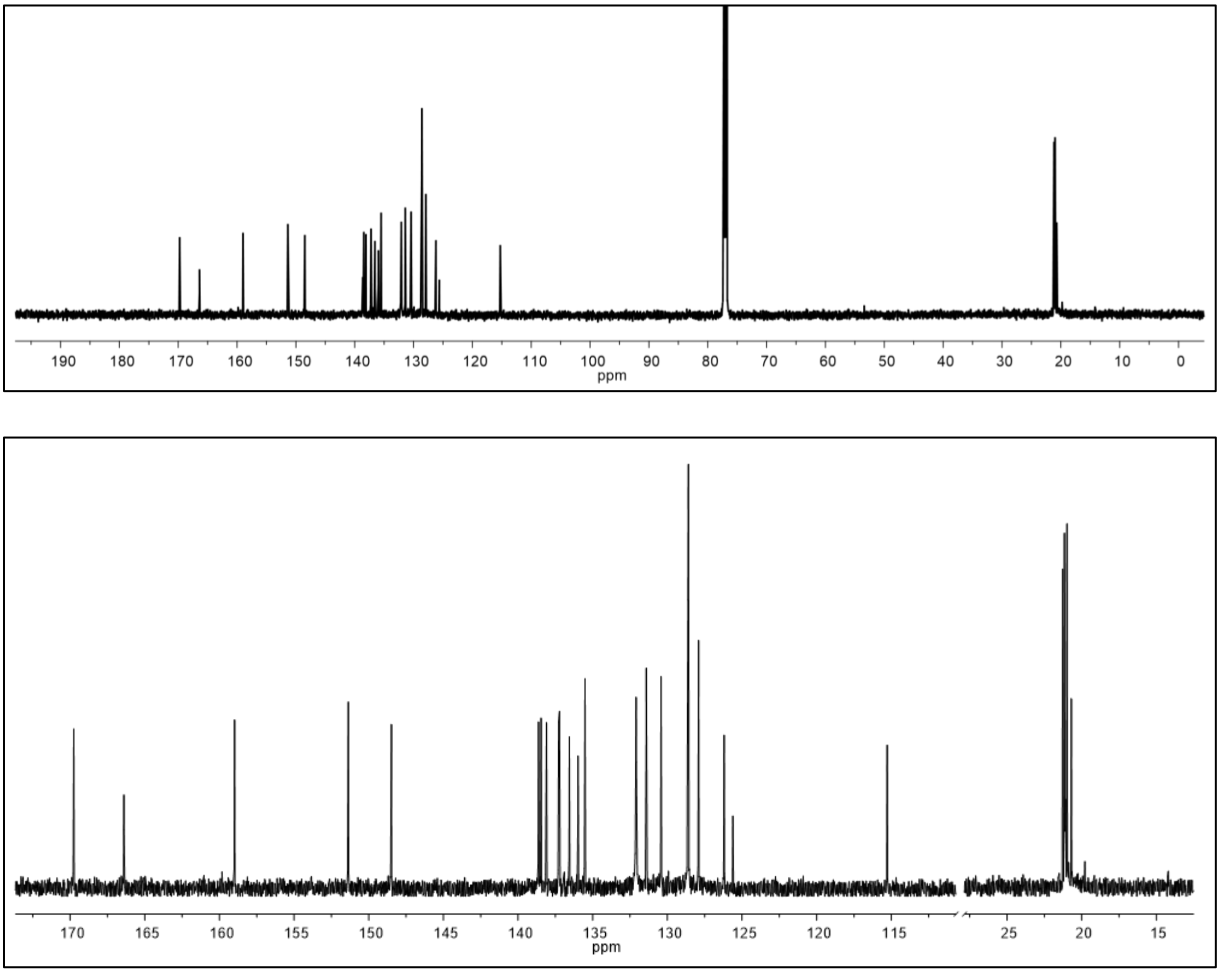


**Figure S30. $^{13}C$ NMR spectrum (125 MHz, $CDCl_3$, 300 K) of 7; (top: the whole spectral range, bottom: the most informative region).**

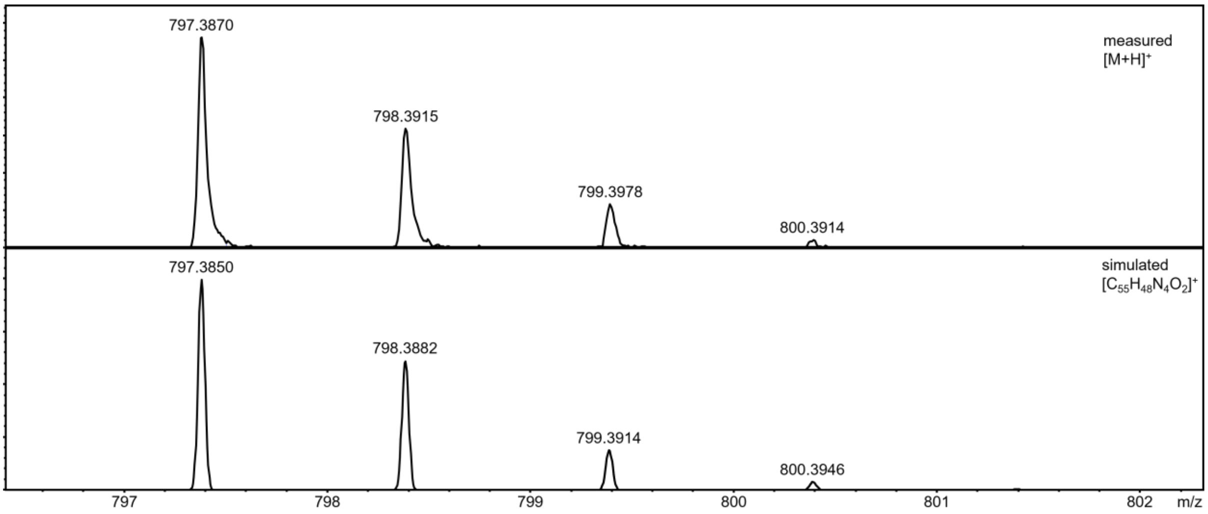


**Figure S31. Selected region of HRMS ESI (+MS) spectrum of 7.**

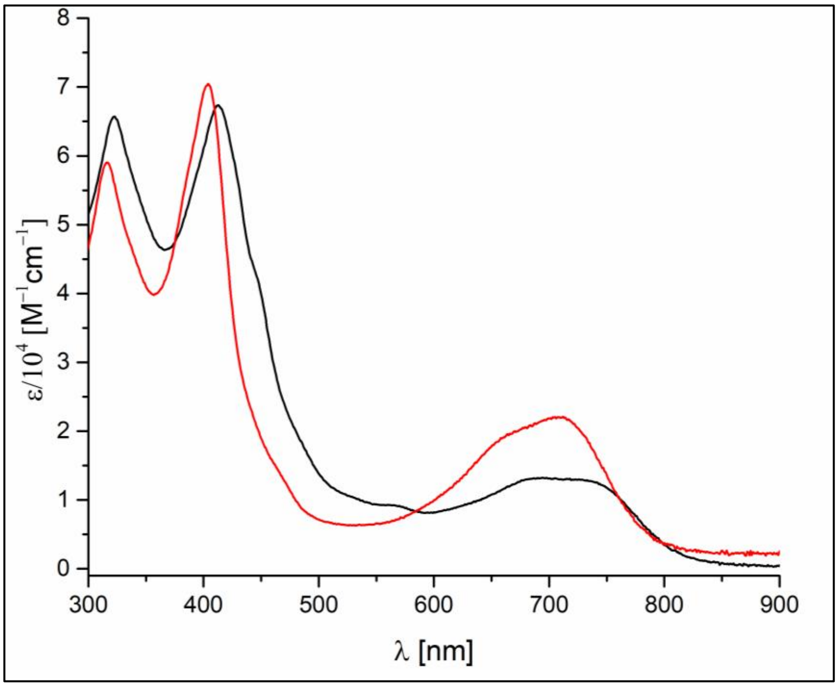


**Figure S32. UV-Vis spectra of 7 (black line) and 8 (red line).**

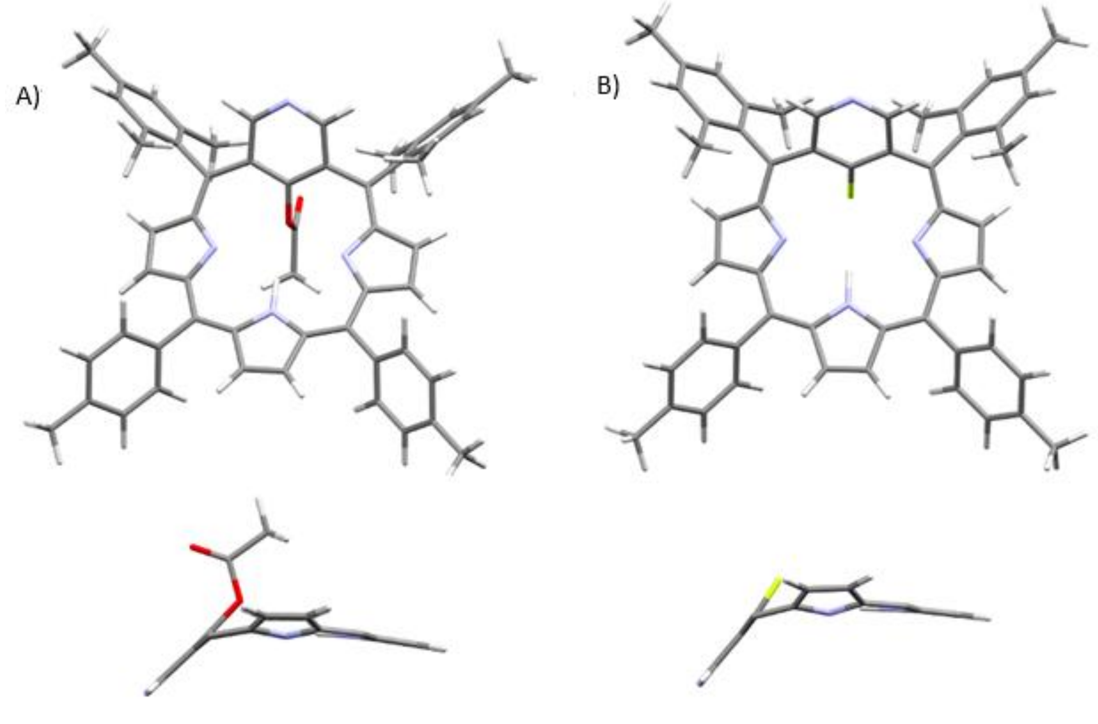


**Figure S33. The DFT-optimised model of 7 and 8.**

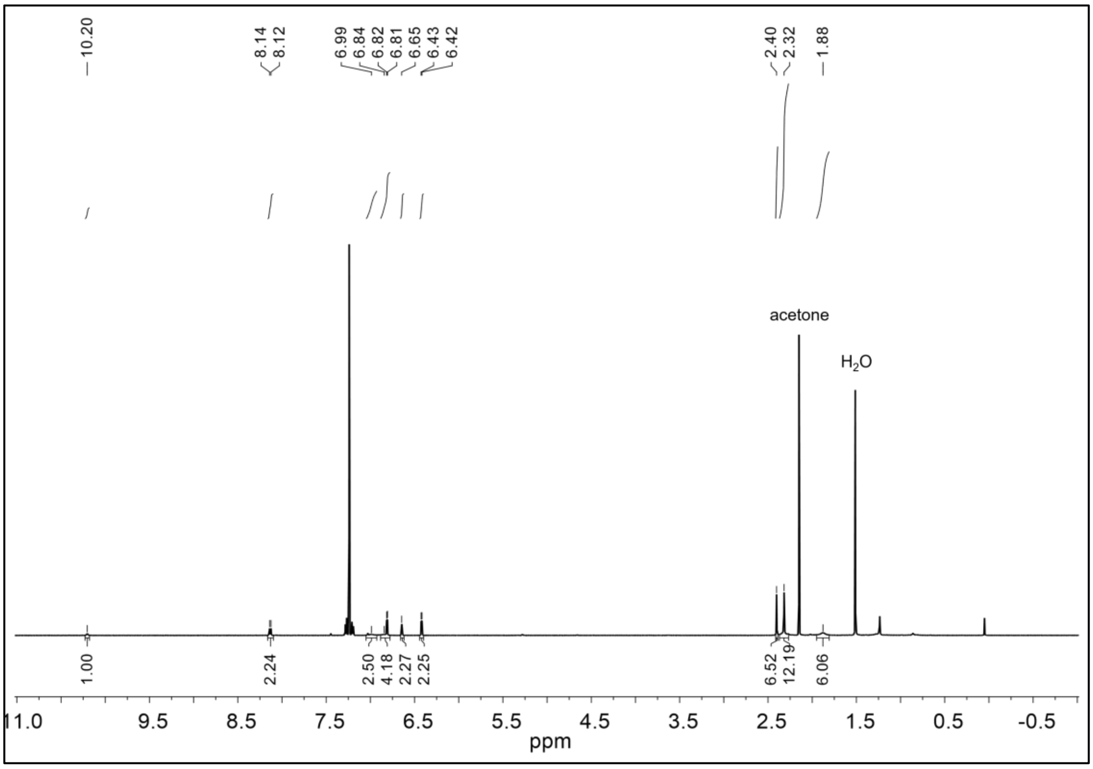


**Figure S34.** $^1$**H NMR spectrum of 8 (600 MHz,** $CD_2Cl_2$**, 300 K.**

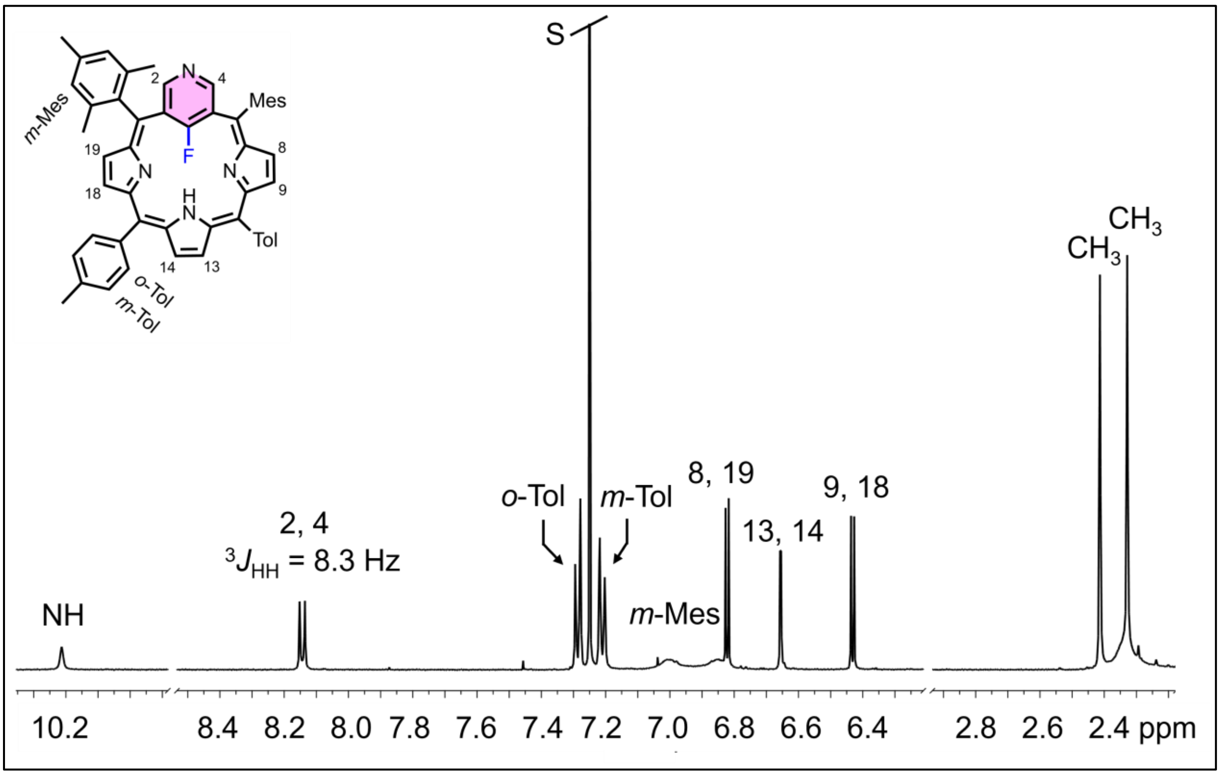


**Figure S35. Signals assignment; 1H NMR spectrum of 8; 500 MHz, CDCl3, 300 K (selected ranges).**

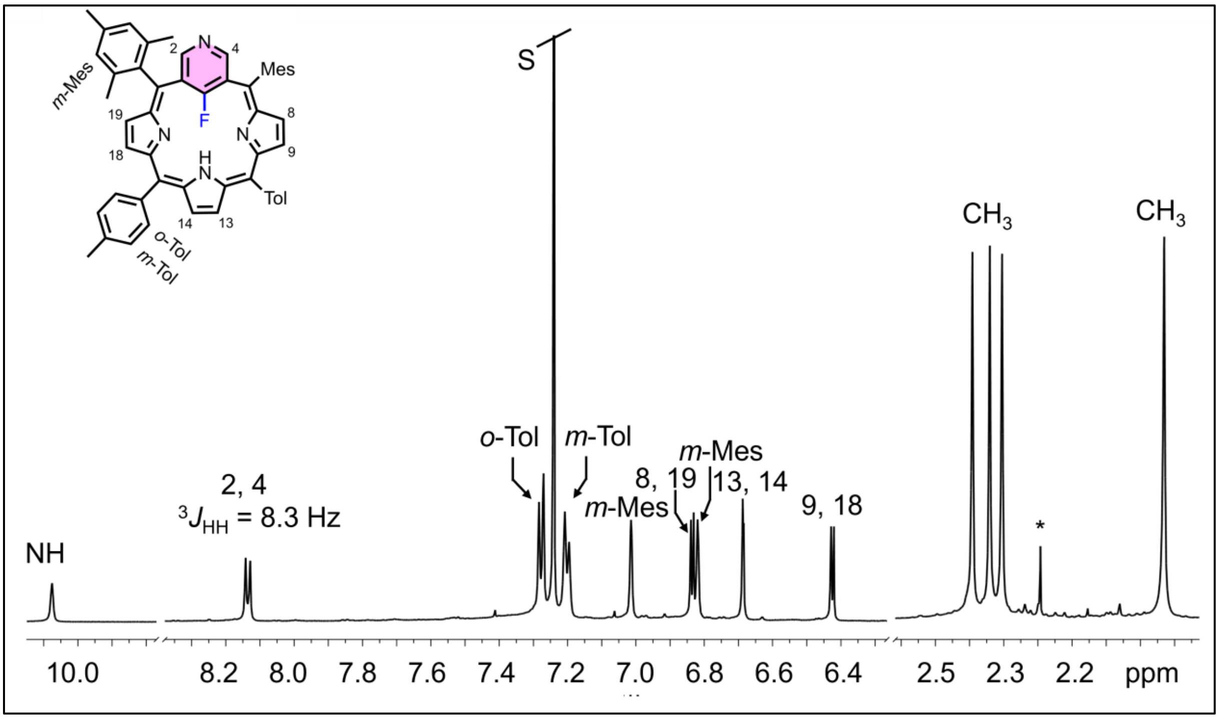


**Figure S36. Signals assignment; $^1$H NMR spectrum of 8; 500 MHz, $CDCl_3$, 230 K (selected ranges).**

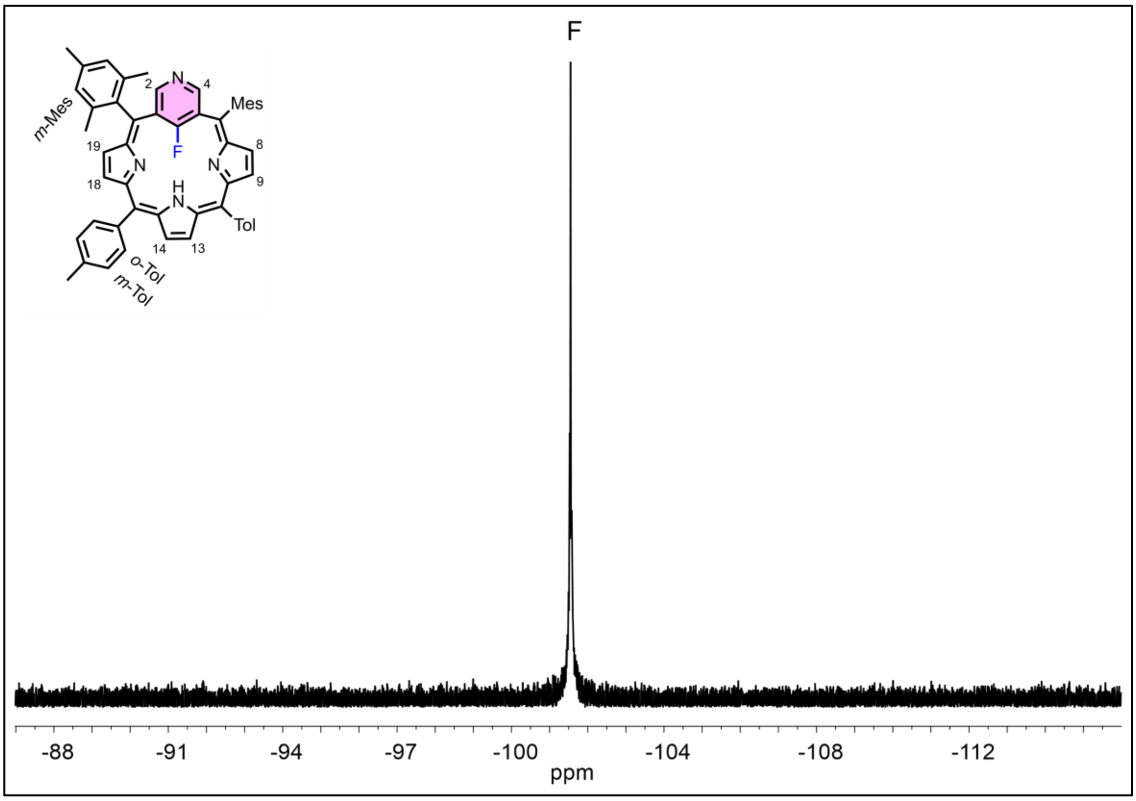


**Figure S37. Signal assignment; $^{19}$F NMR spectrum of 8; 470 MHz, $CDCl_3$, 300 K (selected range).**

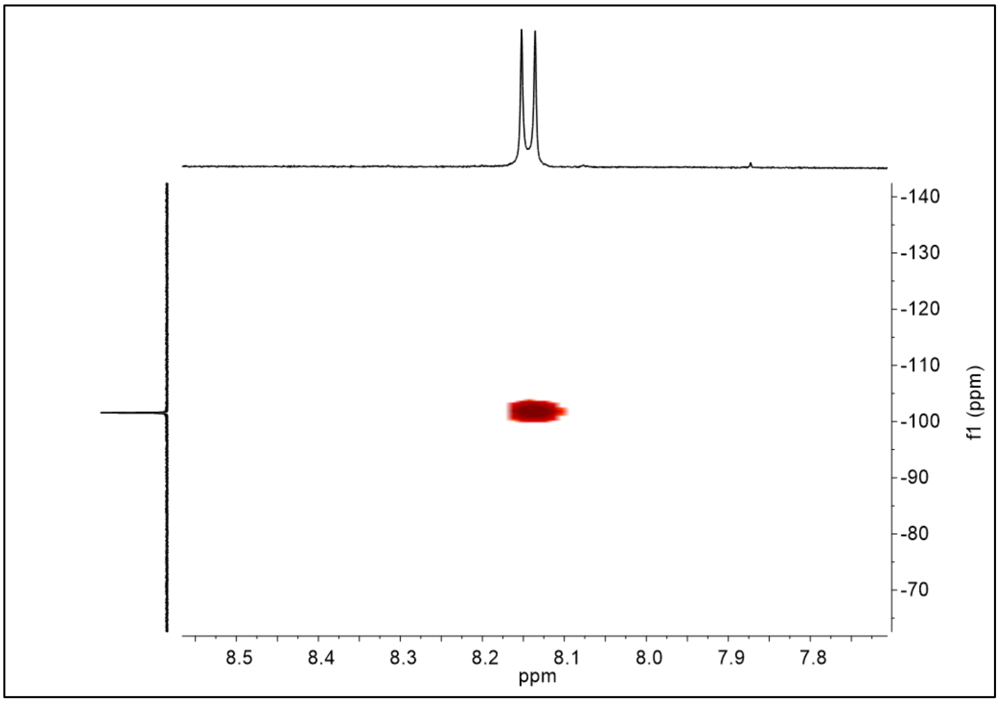


**Figure S38.** $^{1}H$-$^{19}F$ **COSY spectrum (500 MHz, $CDCl_3$, 300 K) of 8.**

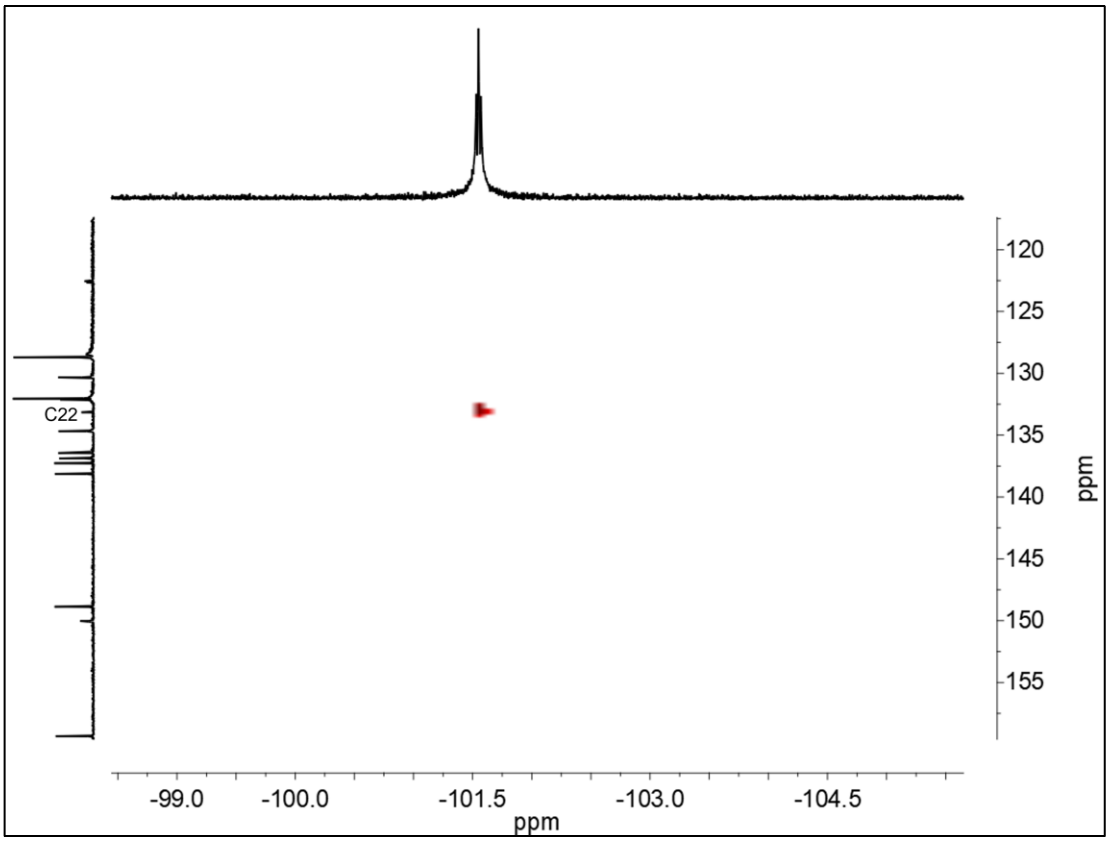


**Figure S39.** $^{13}C$-$^{19}F$ **HSQC spectrum (500 MHz, $CDCl_3$, 300 K) of 8.**

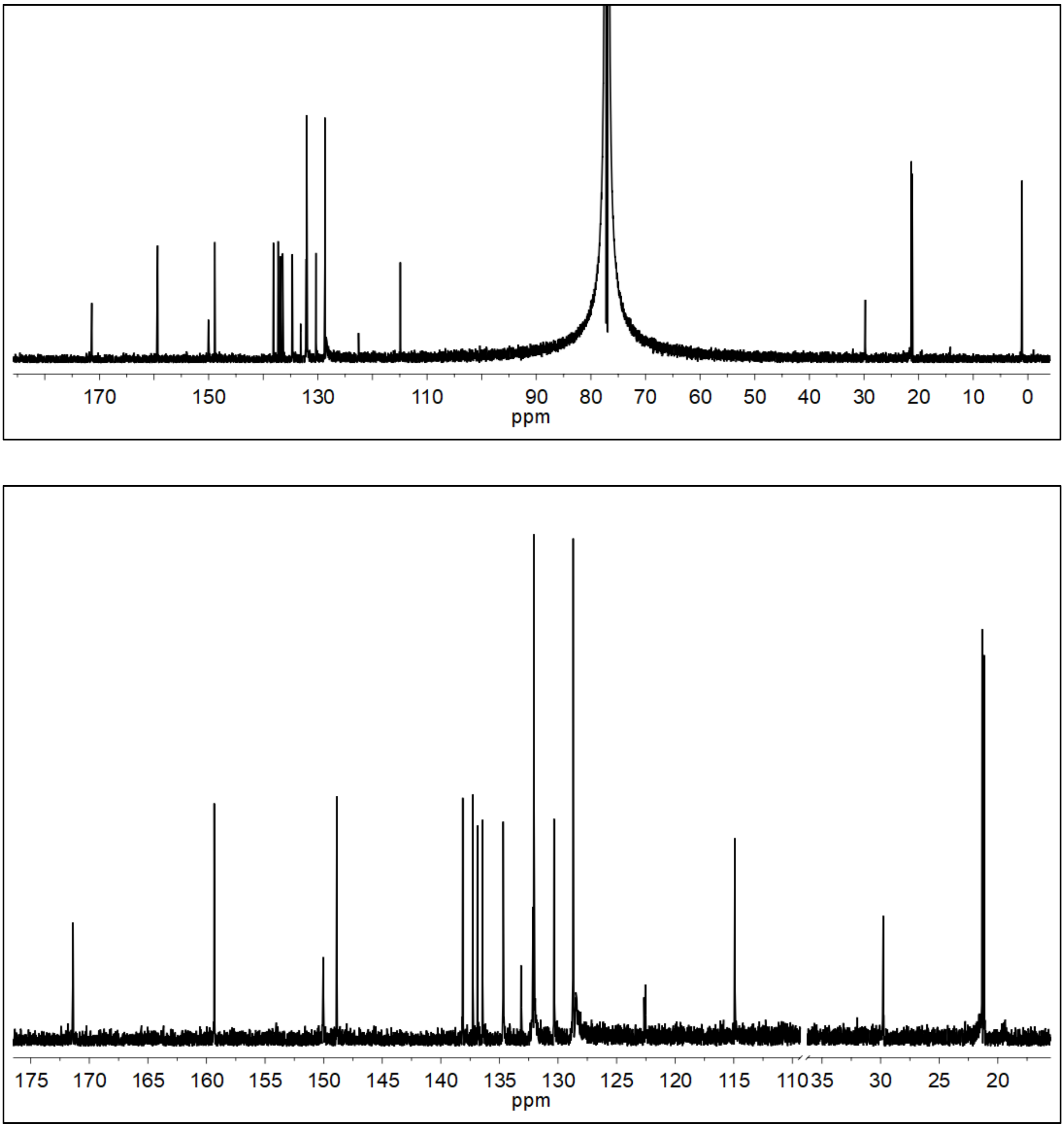


**Figure S40.** $^{13}C$ **NMR spectrum (125 MHz,** $CD_2Cl_2$**, 300 K) of 8; (top: the whole spectral range, bottom: the most informative region).**

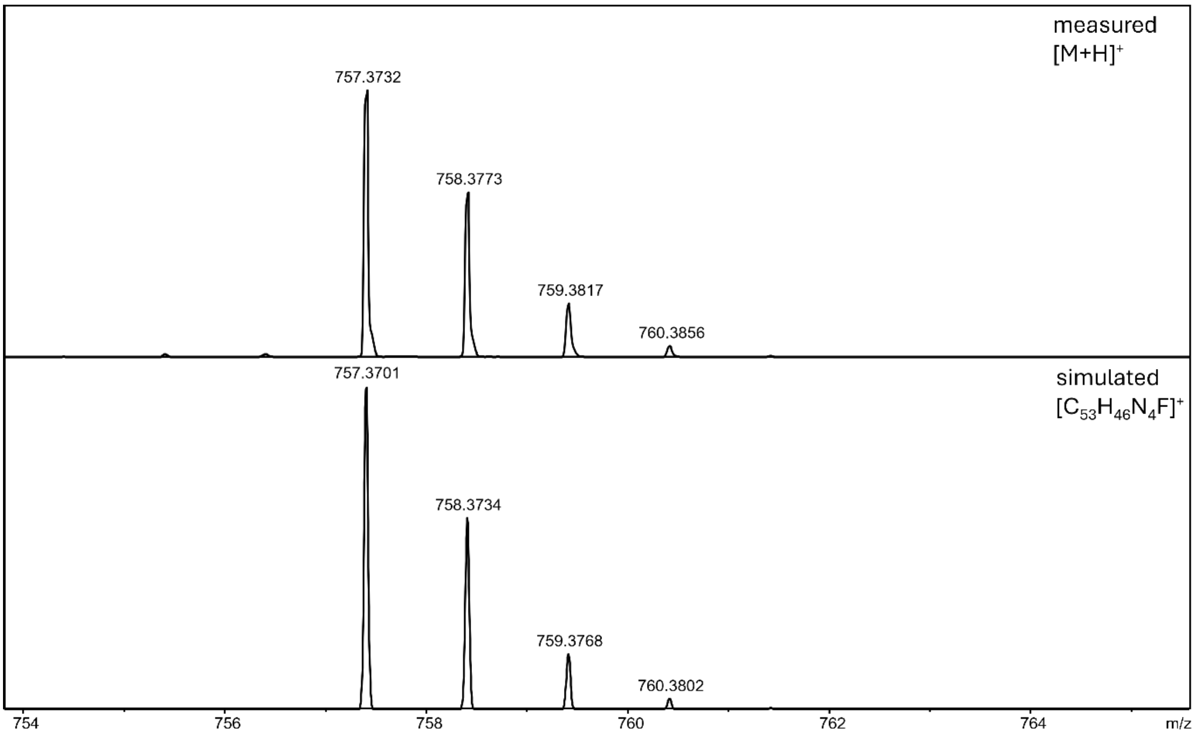


**Figure S41. Selected region of HRMS ESI (+MS) spectrum of 8.**

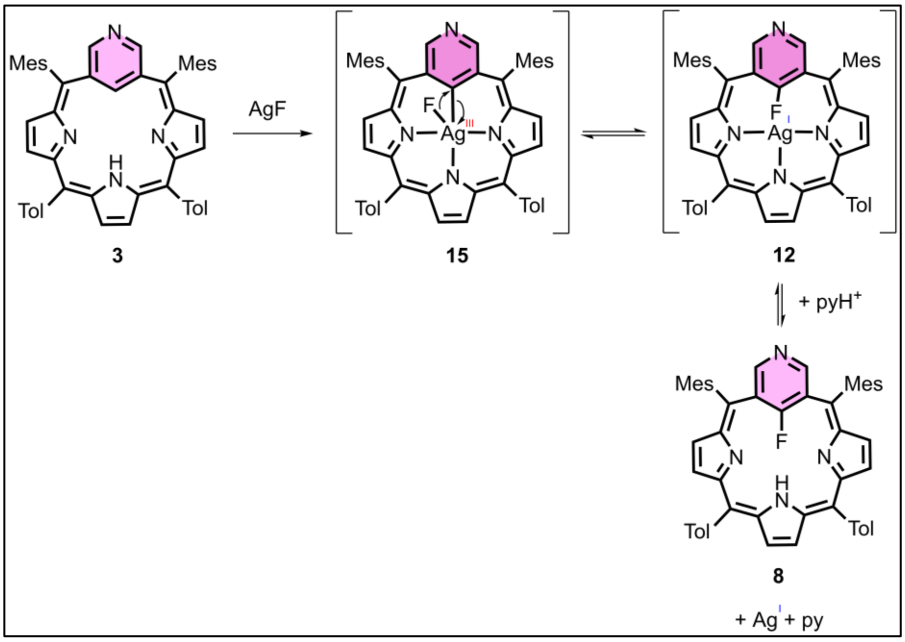


**Scheme S1. The postulated pathway of forming compound 8.**

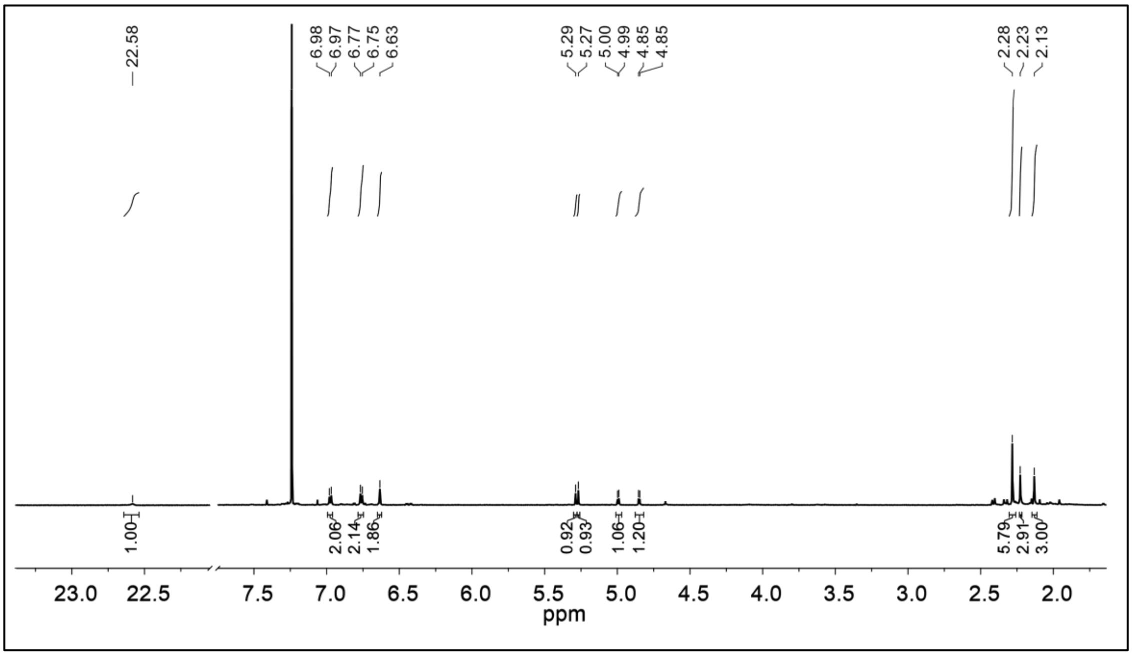


**Figure S42. $^1$H NMR spectrum of 9 (500 MHz, $C_6D_6$, 300 K).**

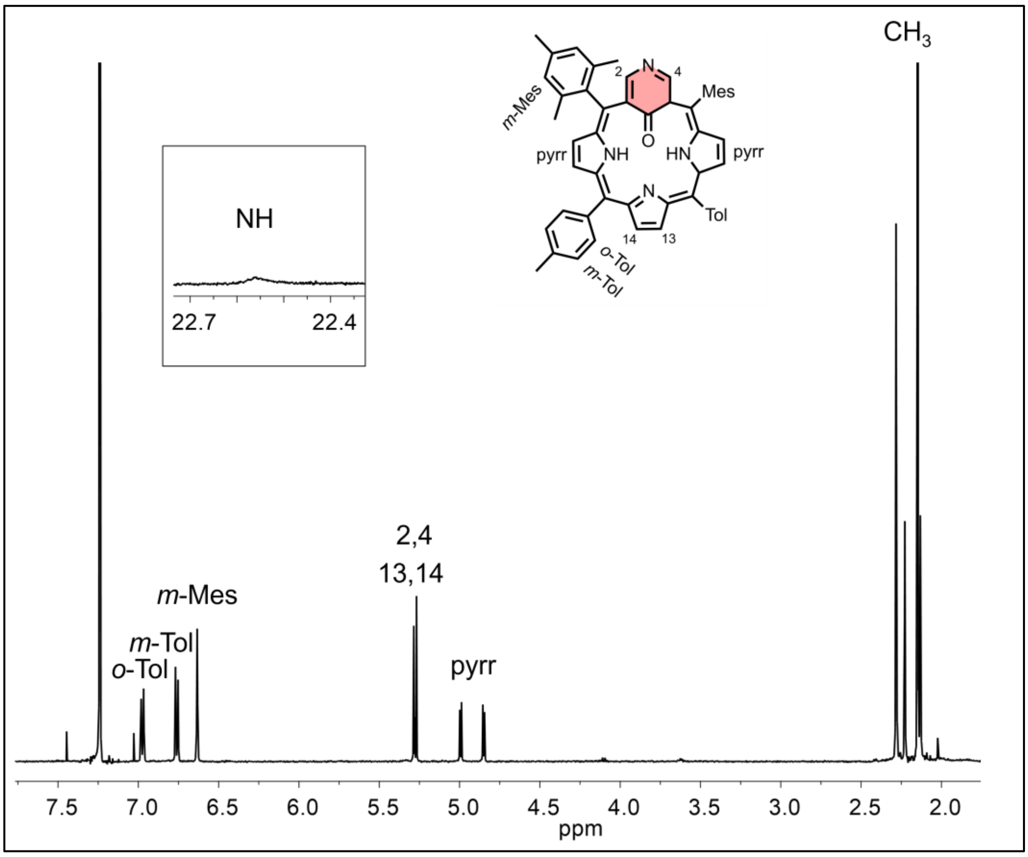


**Figure S43. Signals assignment; $^1$H NMR spectrum of 9; 500 MHz, $CDCl_3$, 300 K (selected ranges).**

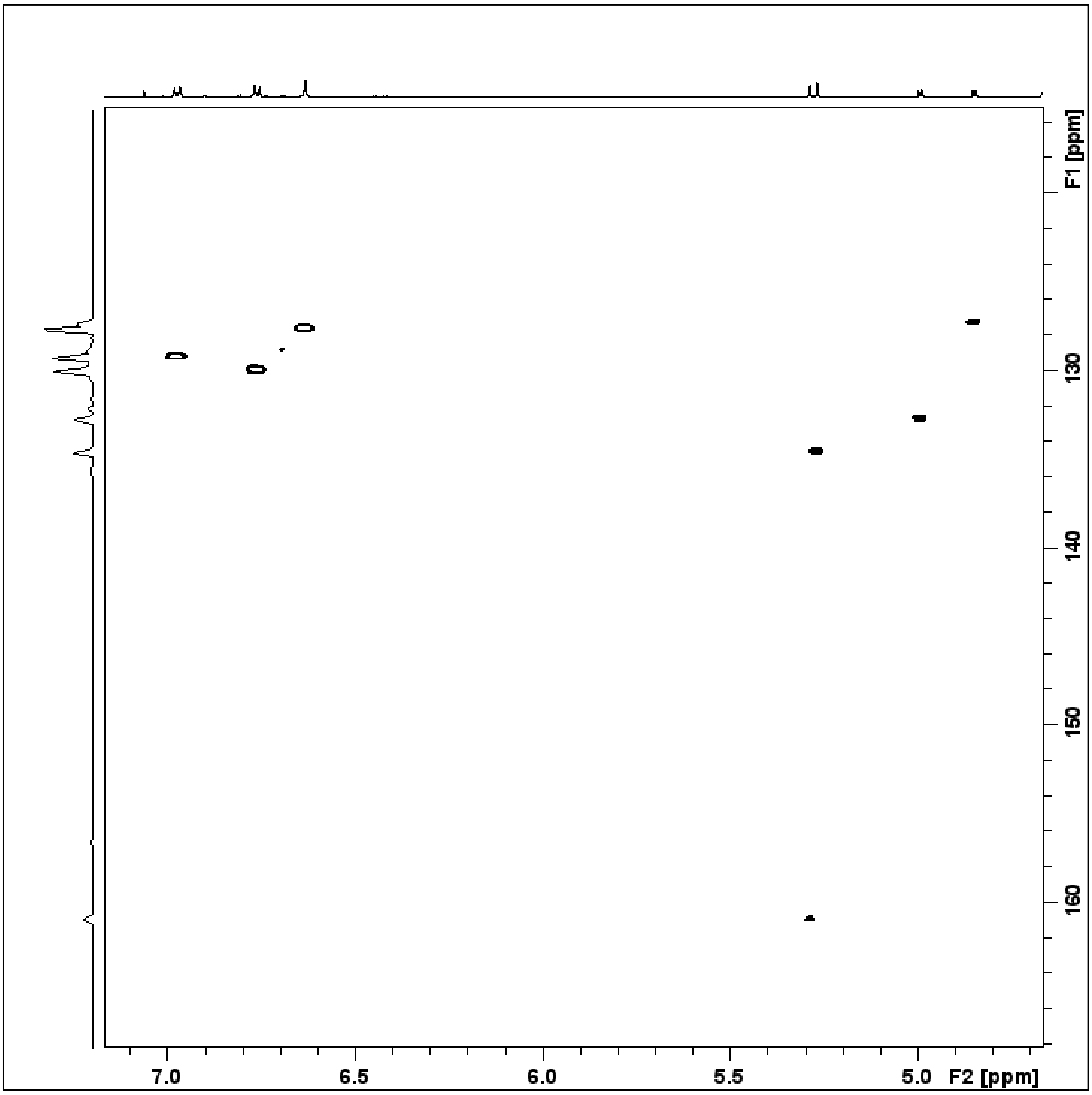


**Figure S44.** **$^1H$-$^{13}C$ HSQC spectrum (600 MHz, $CDCl_3$, 300 K) of 9 (selected range).**

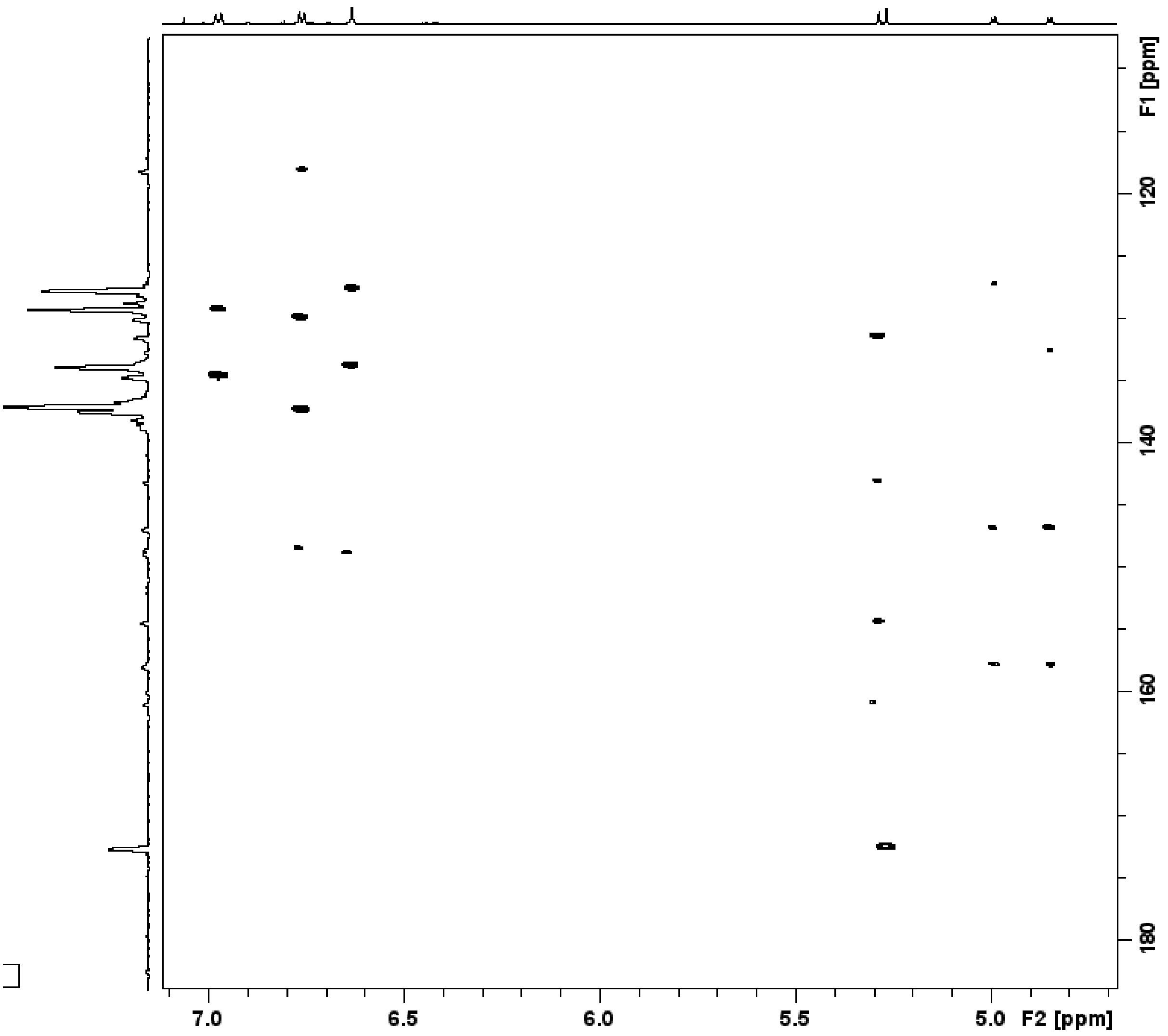


**Figure S45. $^{1}$H-$^{13}$C HMBC spectrum (600 MHz, $CDCl_3$, 300 K) of 9 (selected range).**

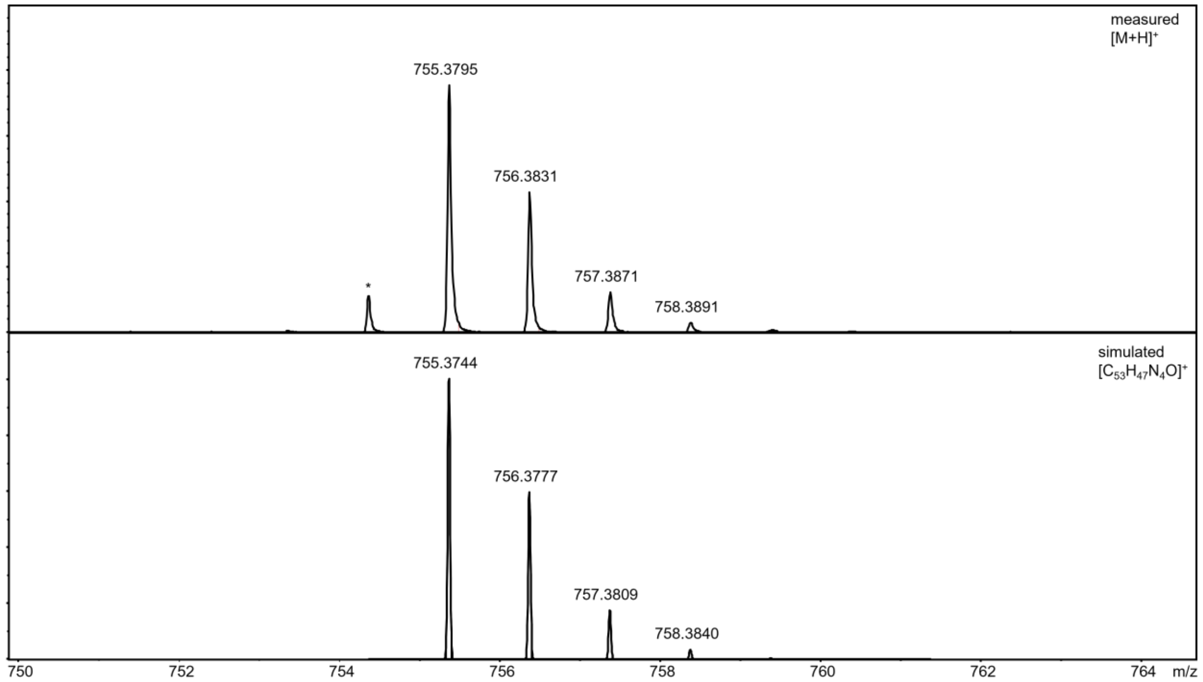


**Figure S46. Selected regions of HRMS ESI (+MS) spectrum of 9.**

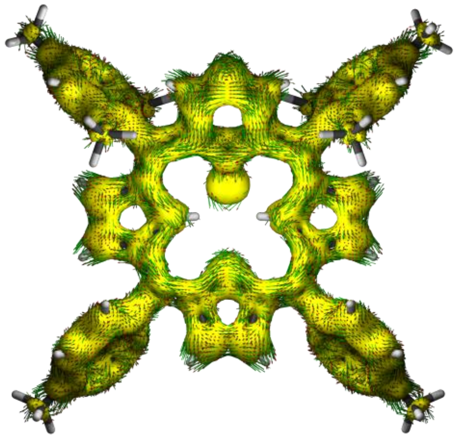

**Figure S47. AICD plot for 9.**

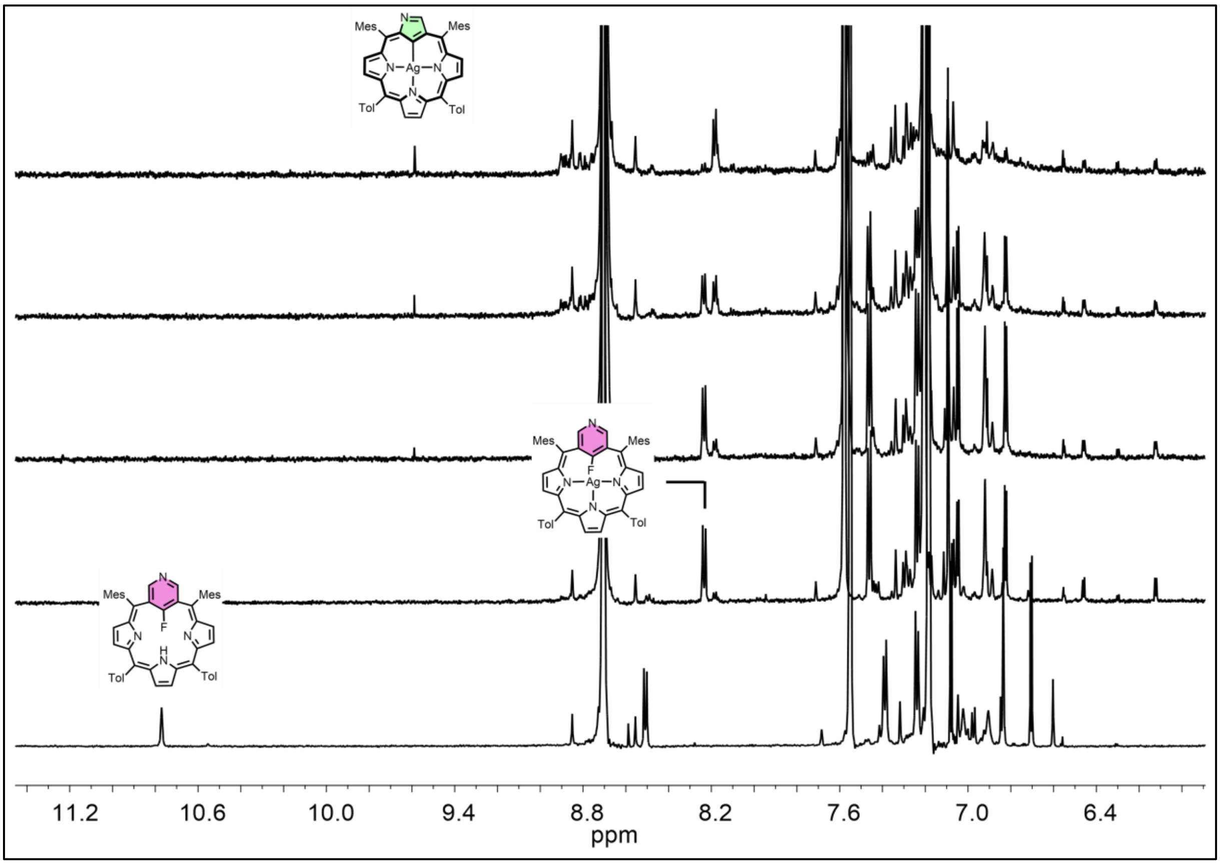


**Figure S48. Conversion of compound 8 into compounds 12 and 10 in the presence of silver ions.**

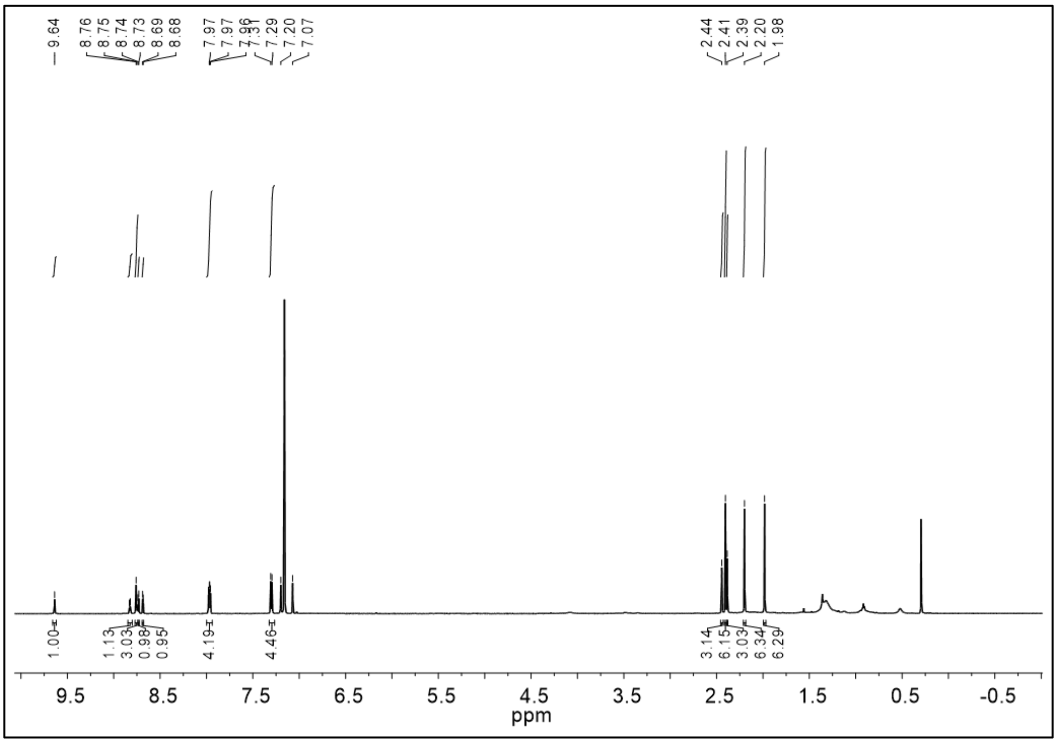


**Figure S49. $^1$H NMR spectrum of 10 (600 MHz, $C_6D_6$, 300 K).**

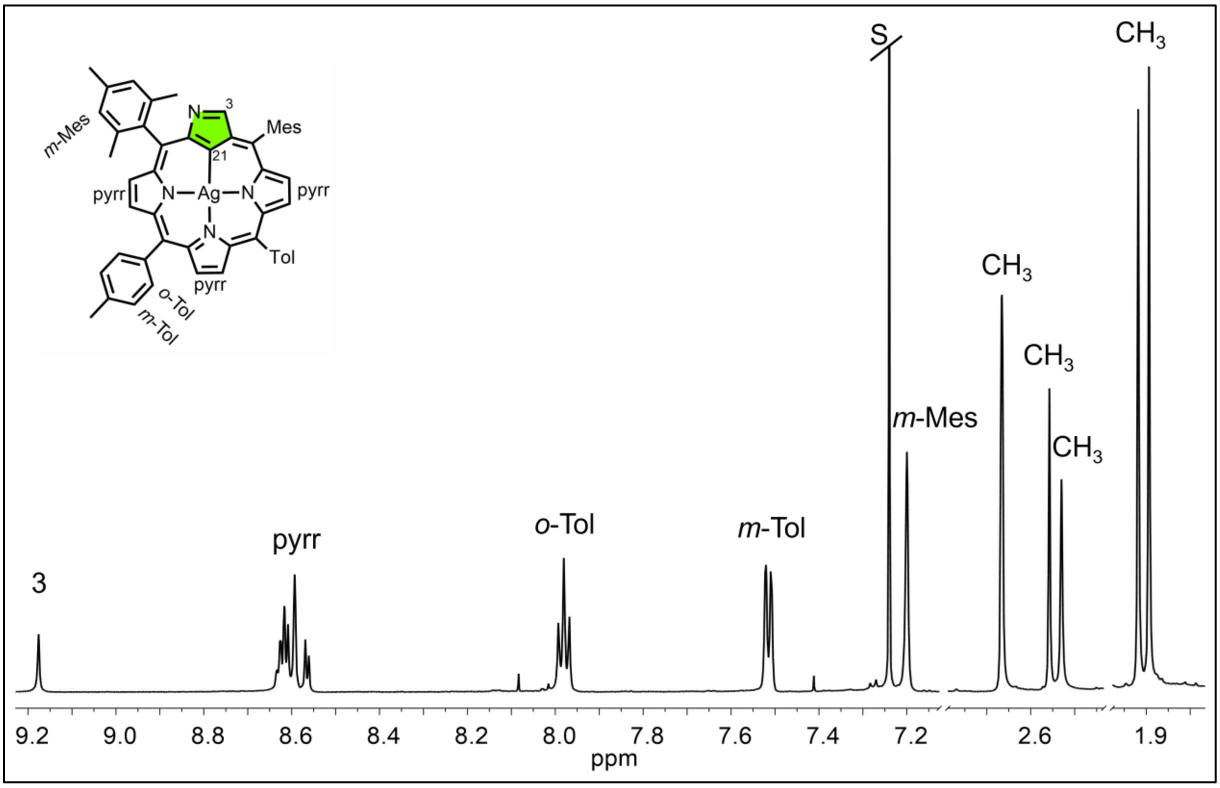


**Figure S50. Signals assignment; $^{1}H$ NMR spectrum of 10; 500 MHz, $CDCl_3$, 300 K (selected ranges).**

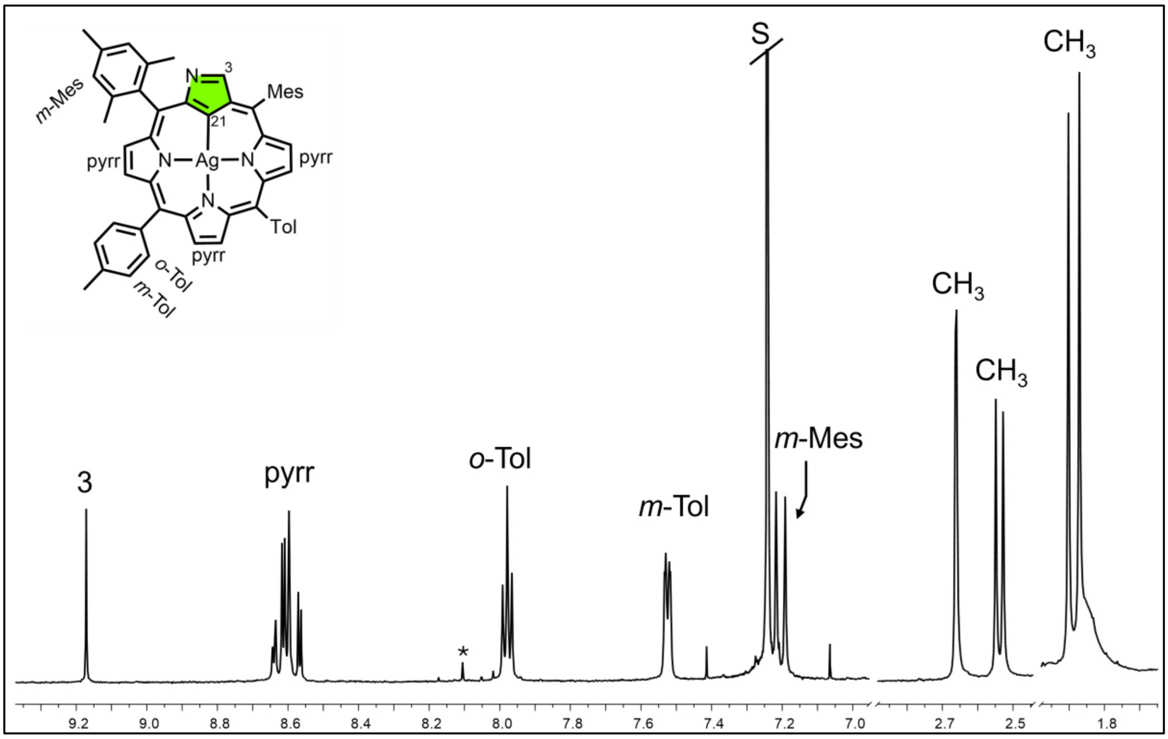


**Figure S51. Signals assignment; $^{1}H$ NMR spectrum of 10; 500 MHz, $CDCl_3$, 230 K (selected ranges).**

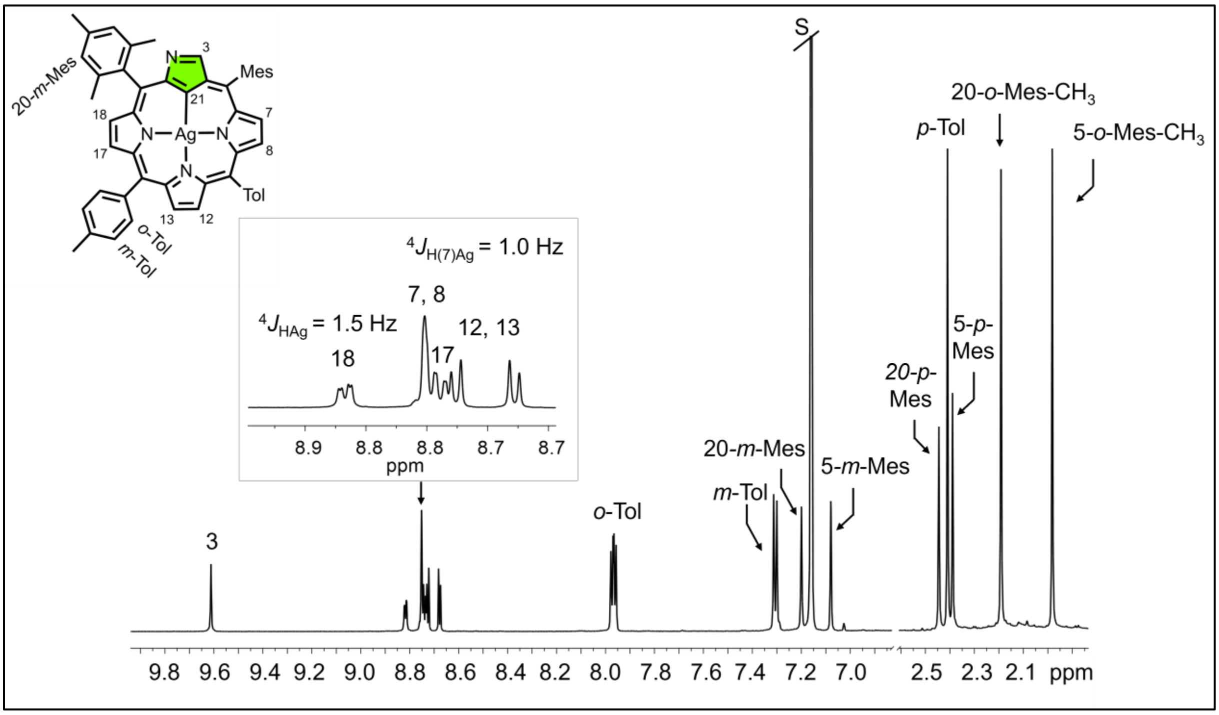


**Figure S52. Signals assignment; $^1$H NMR spectrum of 10; 600 MHz, $C_6D_6$, 310 K (selected ranges).**

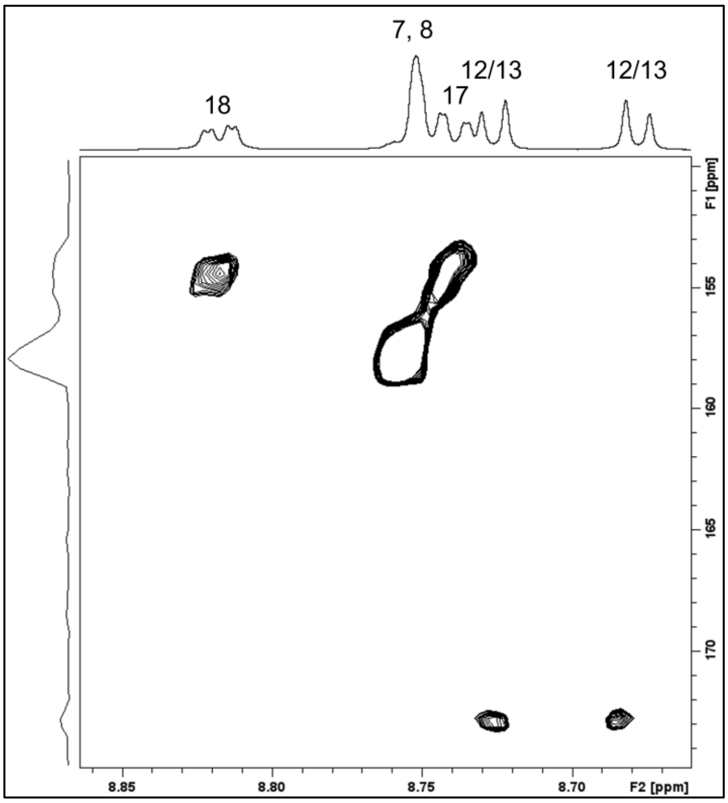


**Figure S53. $^1$H-$^{15}$N HMBC spectrum (600 MHz, $C_6D_6$, 310 K) of 10.**

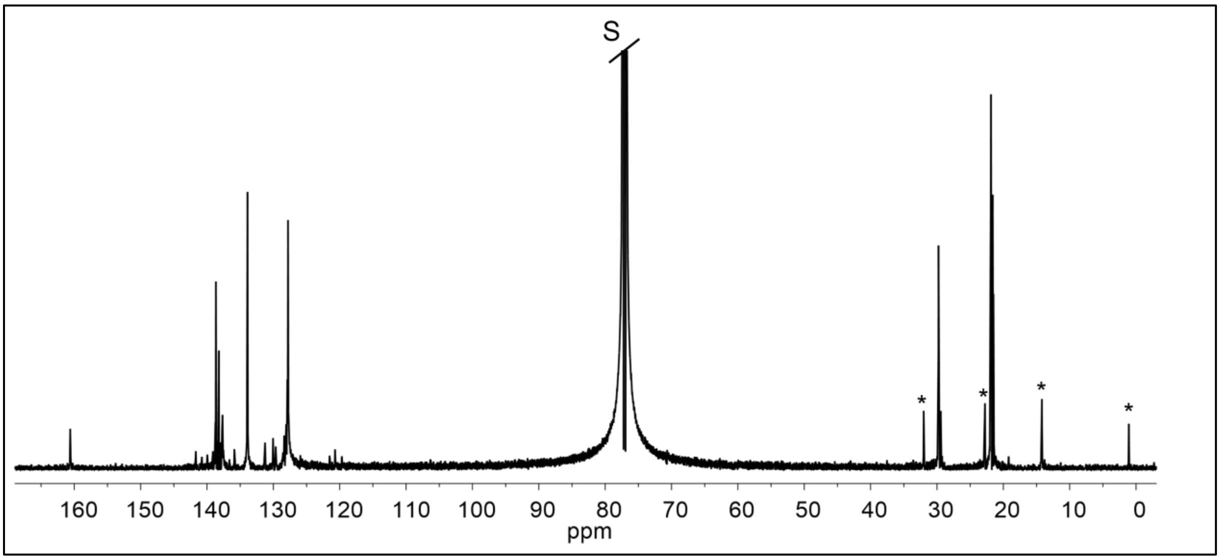


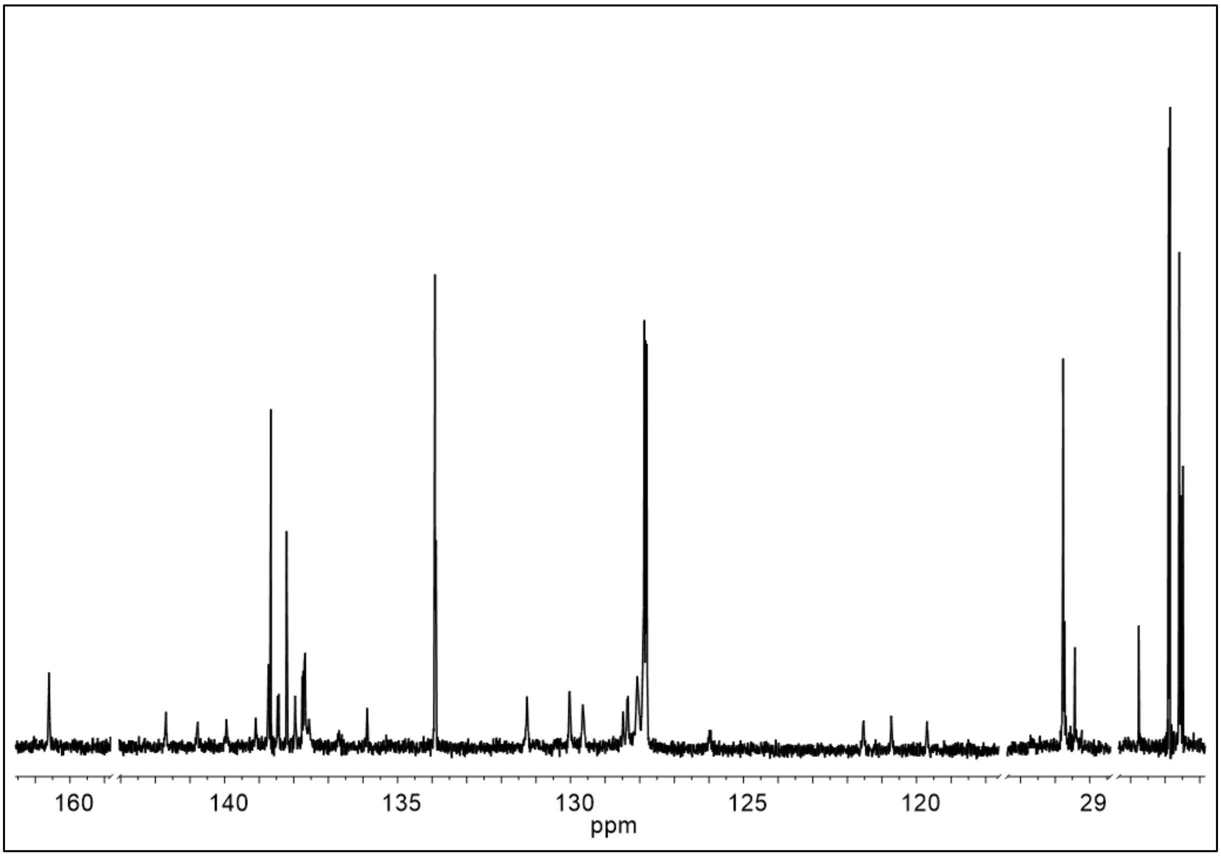


**Figure S54. $^{13}C$ NMR spectrum (125 MHz, $CDCl_3$, 300 K) of 10; (top: the whole spectral range, bottom: the most informative region).**

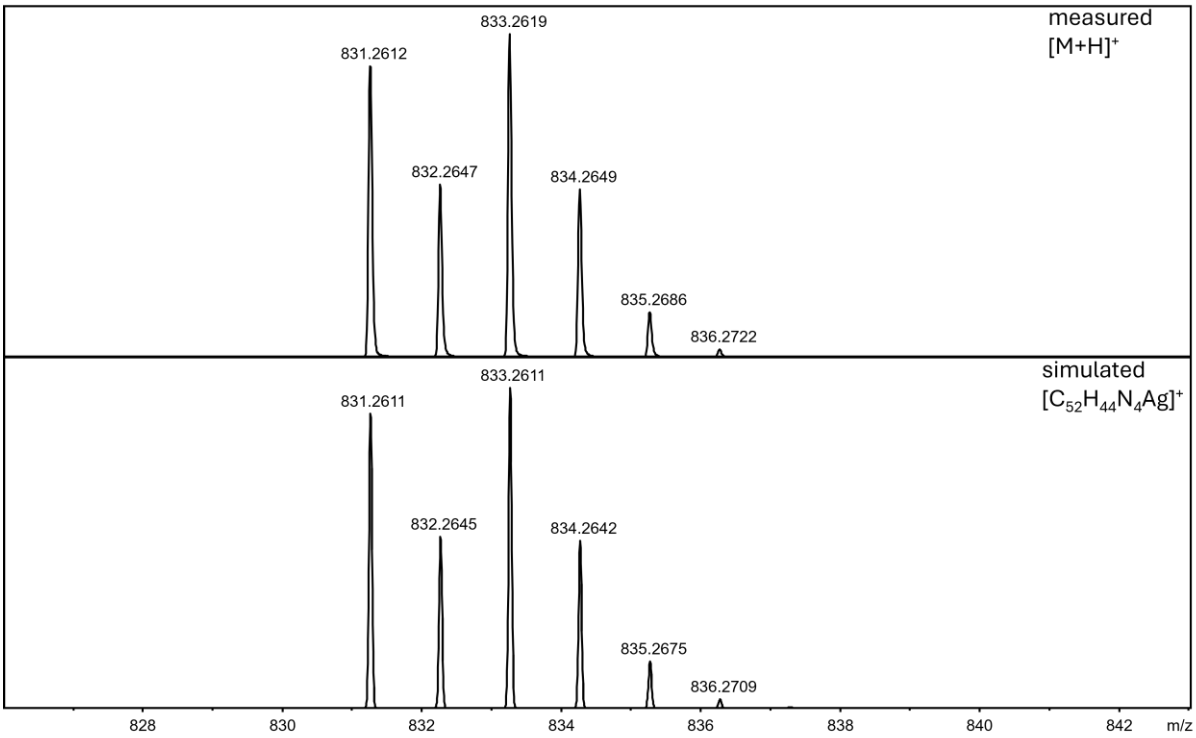


**Figure S55. Selected regions of HRMS ESI (+MS) spectrum of 10.**

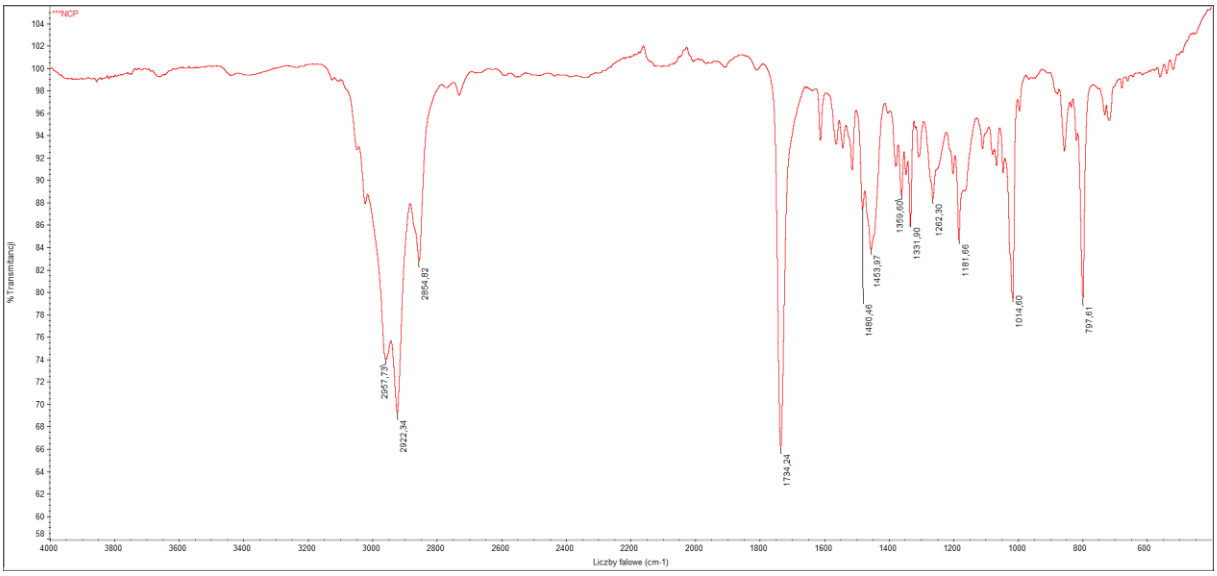


**Figure S56. IR spectrum of 10.**

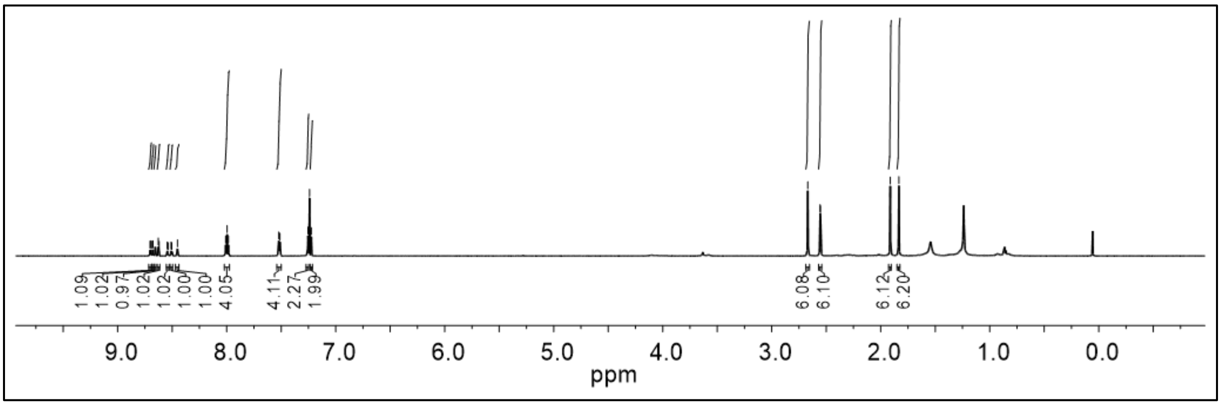


**Figure S57. $^1H$ NMR spectrum of 11 (600 MHz, $CD_2Cl_2$, 300 K).**

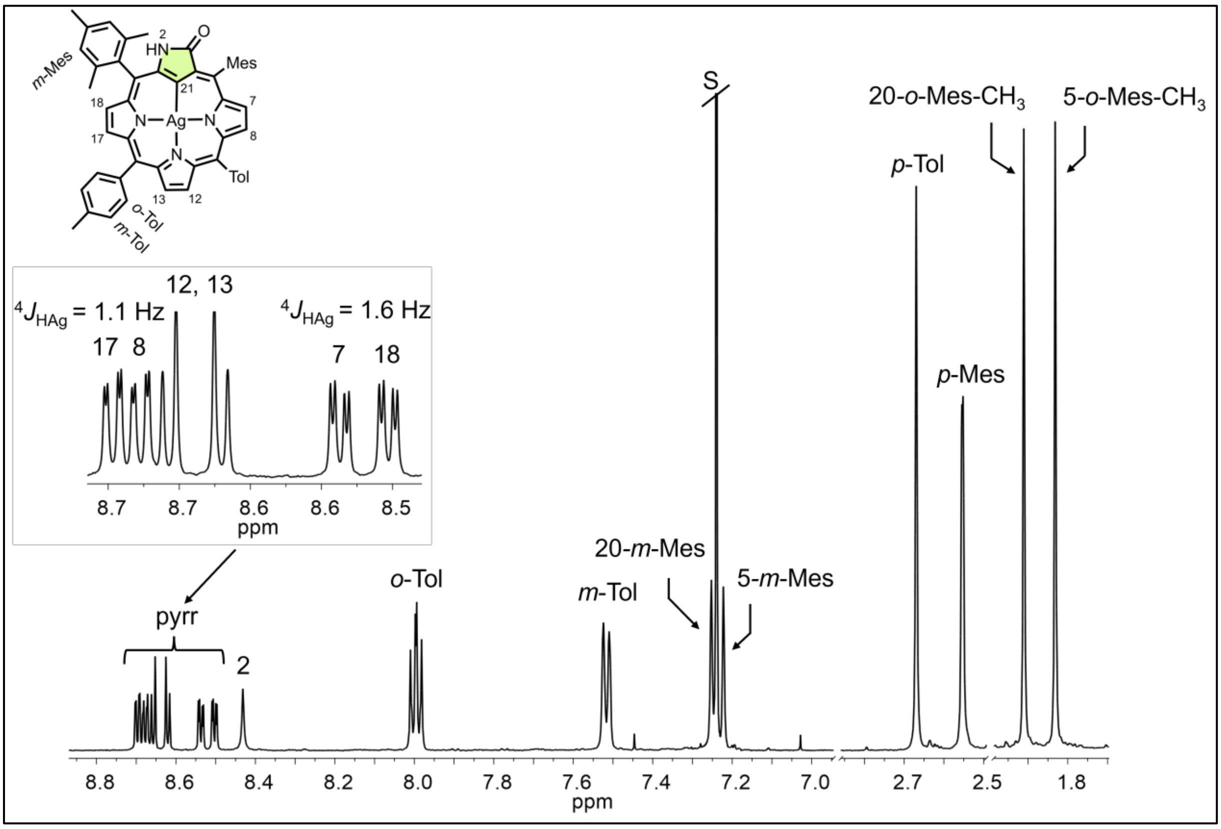


**Figure S58. Signals assignment; $^1H$ NMR spectrum of 11; 500 MHz, $CDCl_3$, 300 K (selected ranges).**

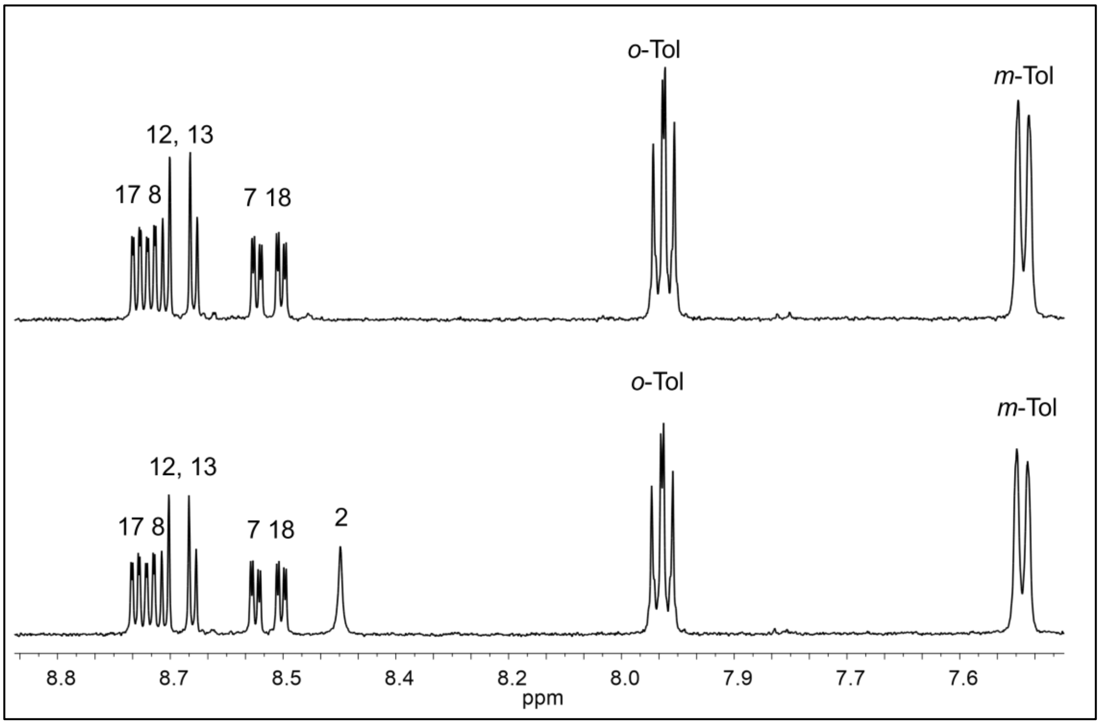


**Figure S59. Comparison of selected range $^1$H NMR spectra of 11 before (bottom) and after (top) adding $D_2O$ (500 MHz, $CDCl_3$, 300K).**

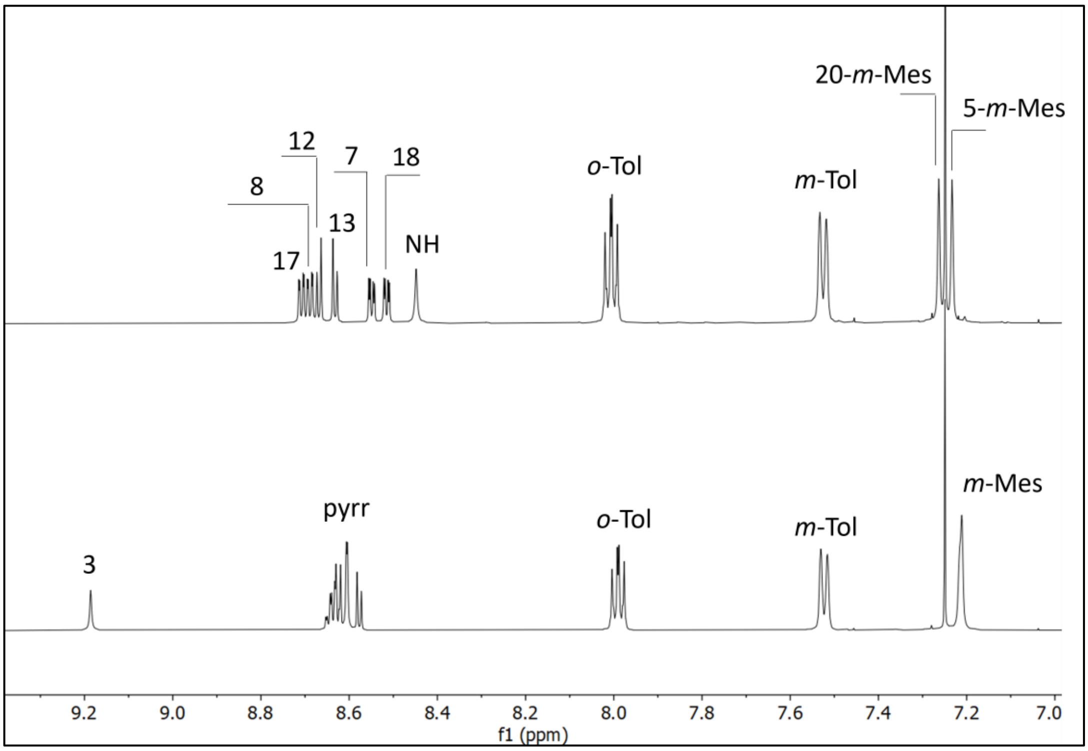


**Figure S60. Part of the $^1$H NMR spectra of 10 and 11 (500 MHz, $CDCl_3$, 300 K).**

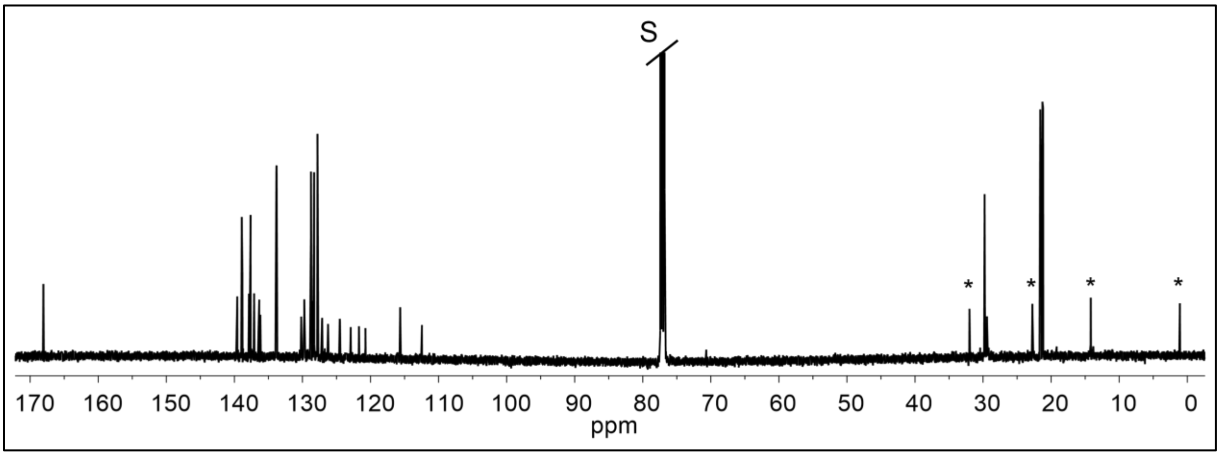


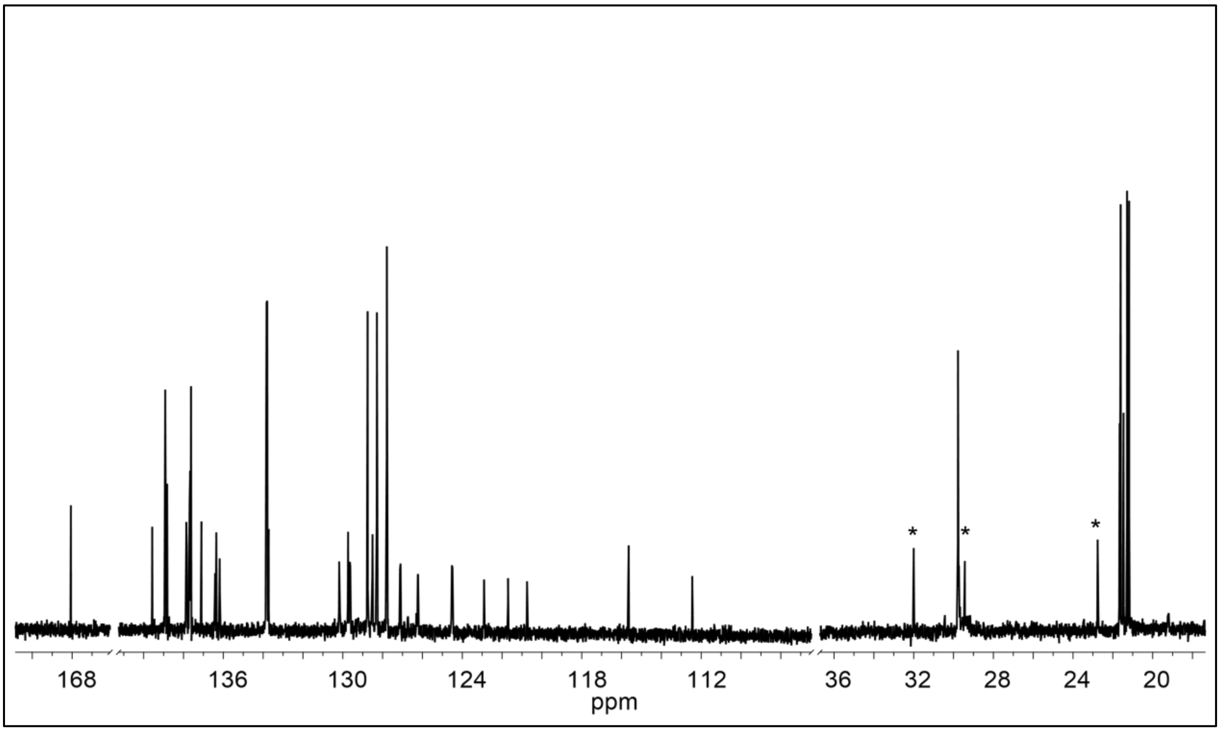


**Figure S61.$^{13}$C NMR spectrum (125 MHz, CDCl$_3$, 300 K) of 11; (top: the whole spectral range, bottom: the most informative region).**

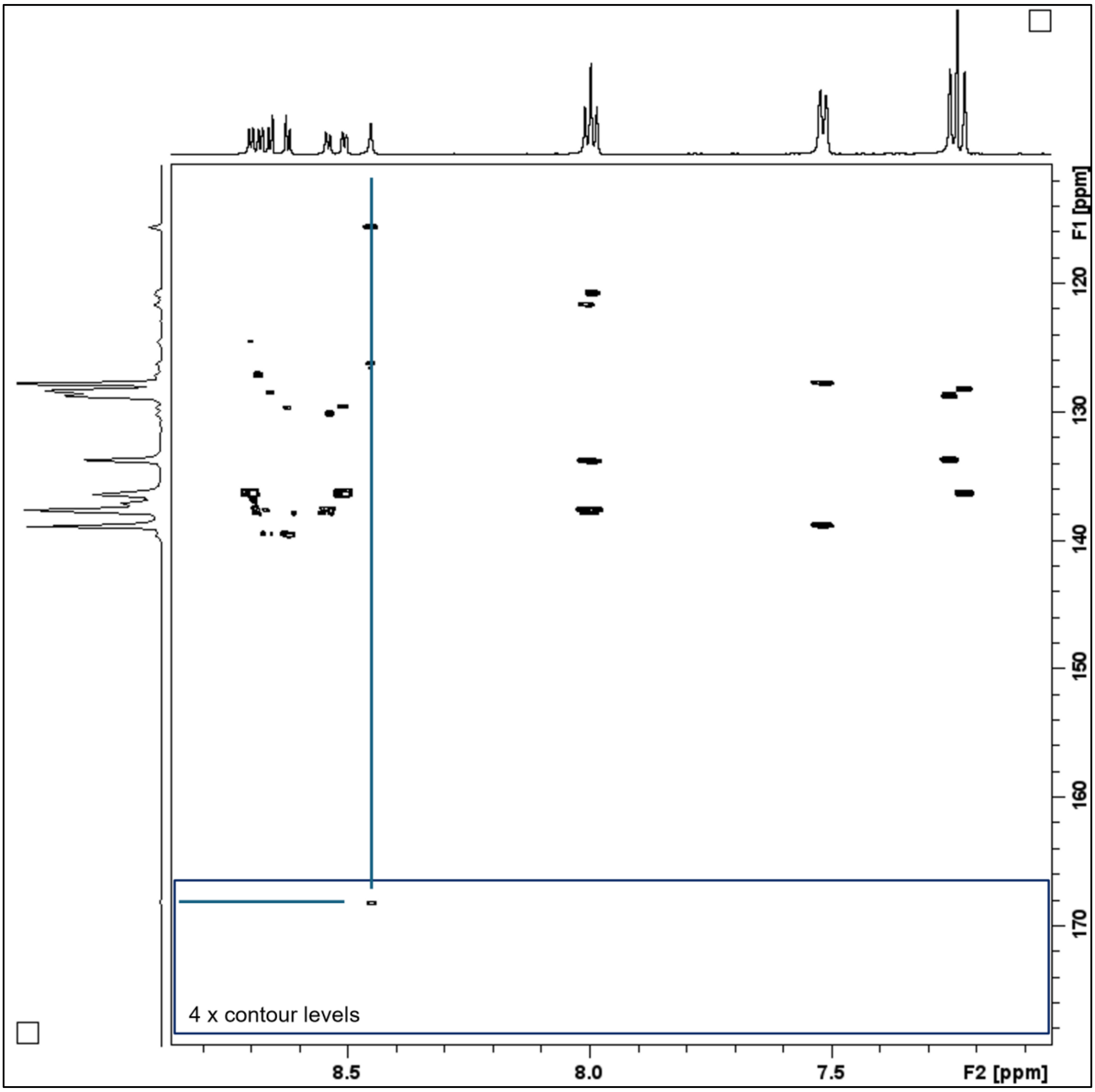


**Figure S62. $^{1}H$-$^{13}C$ HMBC spectrum (600 MHz, $CDCl_3$, 300 K) of 11 (selected range).**

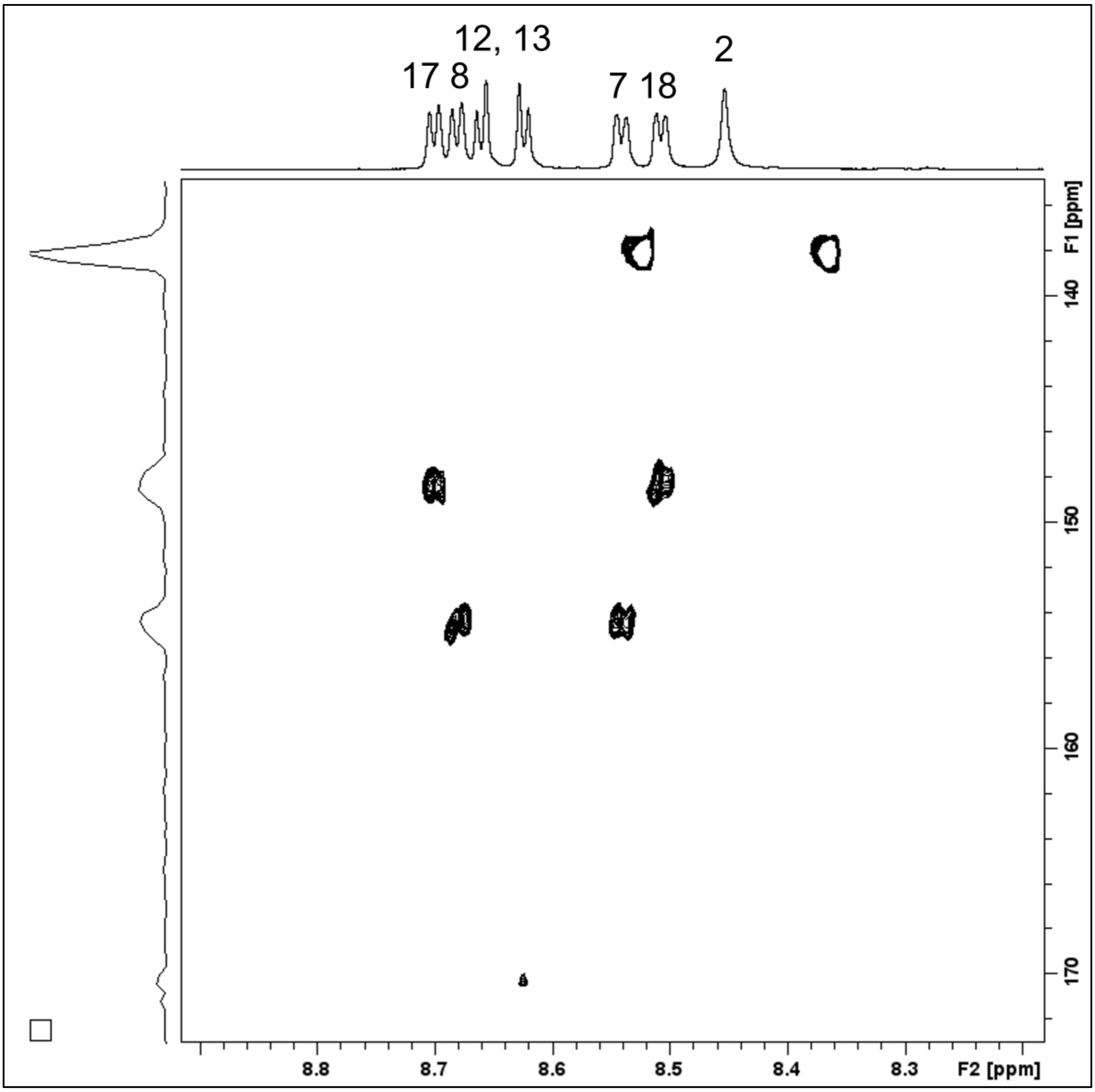


**Figure S63.** $^{1}H$-$^{15}N$ **HMBC spectrum (600 MHz, $CDCl_3$, 300 K) of 11.**

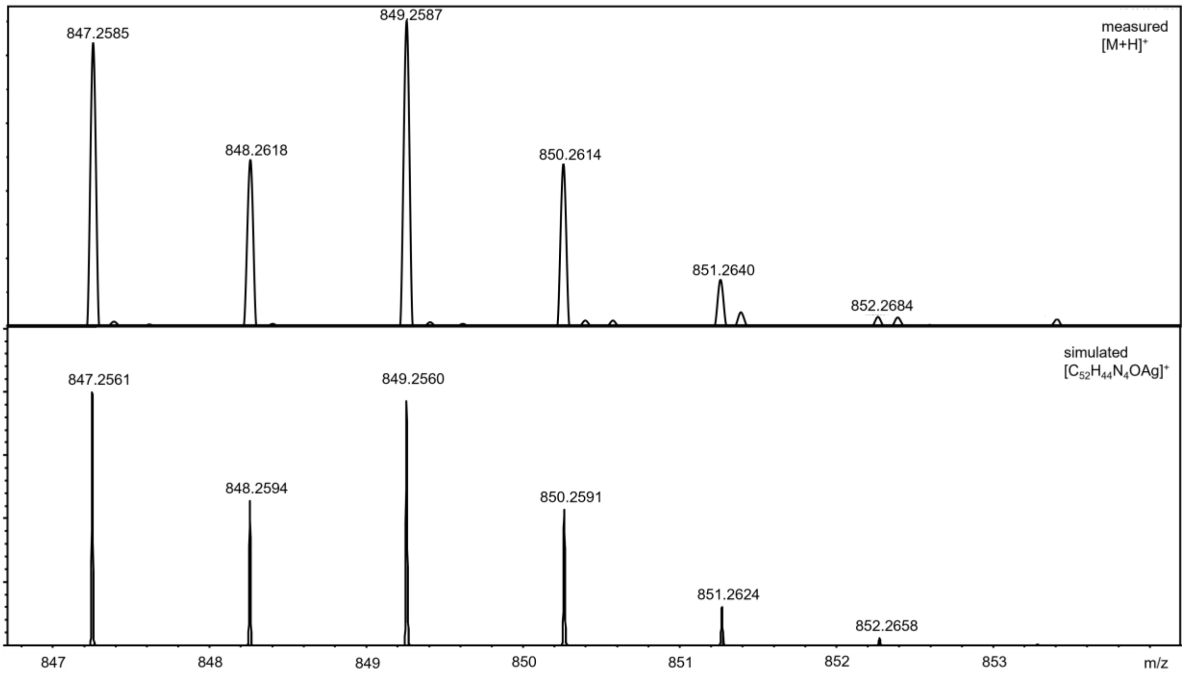


**Figure S64. Selected regions of HRMS ESI (+MS) spectrum of 11.**

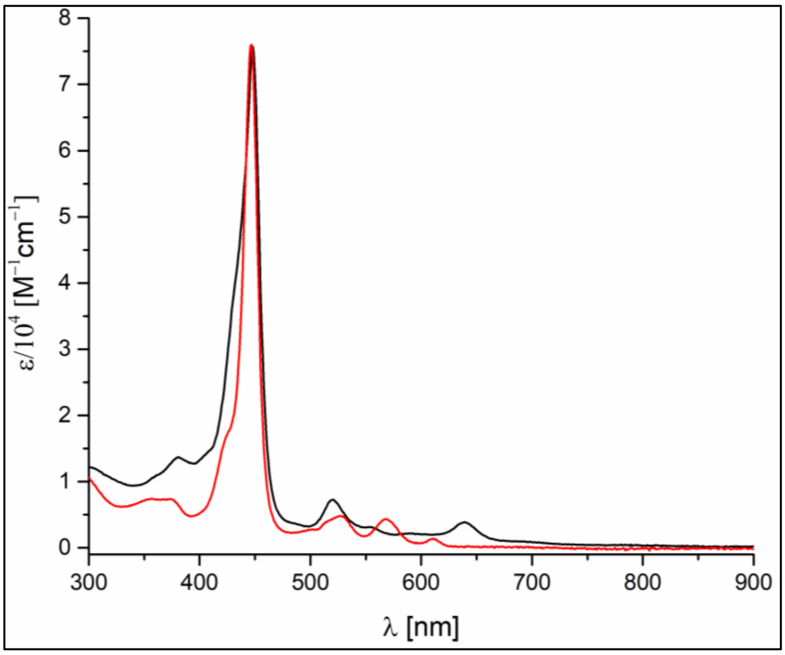


**Figure S65. UV-Vis spectra of 8 (blue line), 10 (black line) and 11 (red line).**

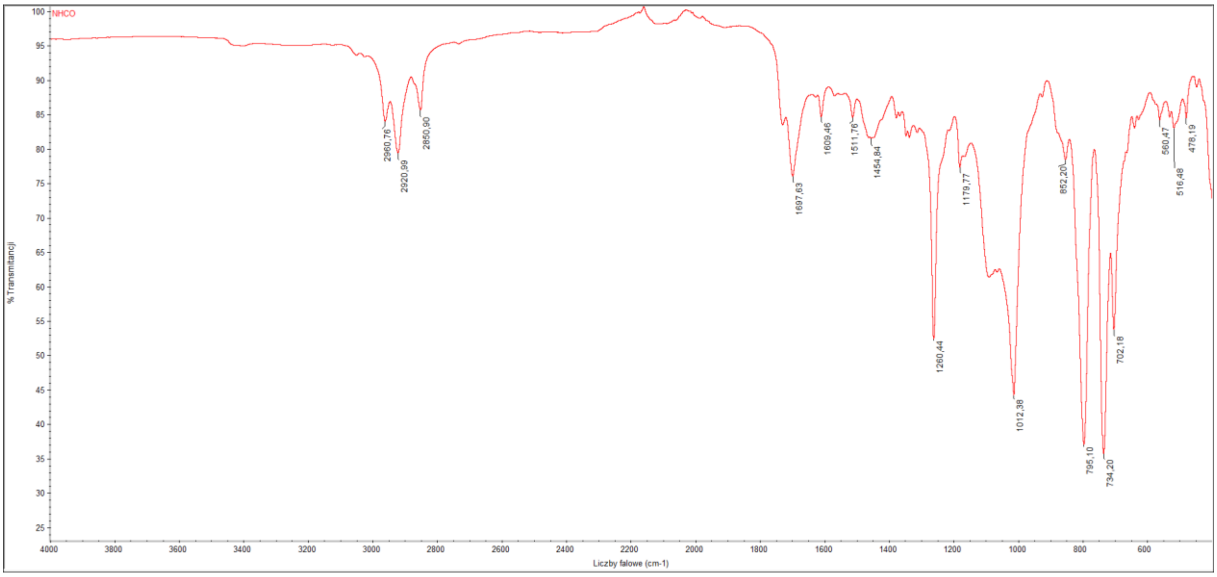


**Figure S66. IR spectrum of 11.**

# Cartesian coordinates

**Coordinates for compound 4**

| | Atomic Number | | Coordinates (Angstroms) X | Y | Z |
|---|---|---|---|---|---|
| 1 | 6 | C | -1.253615 | 4.223769 | 0.675587 |
| 2 | 6 | C | -2.603450 | 4.147389 | 0.668707 |
| 3 | 6 | C | -2.965928 | 2.776699 | 0.366385 |
| 4 | 6 | C | -4.299653 | 2.359876 | 0.169981 |
| 5 | 6 | C | -4.652318 | 1.040296 | -0.098180 |
| 6 | 6 | C | -5.981501 | 0.613857 | -0.502880 |
| 7 | 6 | C | -5.935796 | -0.724281 | -0.687643 |
| 8 | 6 | C | -4.584242 | -1.157552 | -0.374854 |
| 9 | 6 | C | -4.161639 | -2.480935 | -0.356853 |
| 10 | 6 | C | -2.839368 | -2.859947 | -0.026641 |
| 11 | 6 | C | -2.472133 | -4.227977 | 0.280429 |
| 12 | 6 | C | -1.156887 | -4.240079 | 0.594760 |
| 13 | 6 | C | -0.662856 | -2.888296 | 0.444392 |
| 14 | 6 | C | 0.651364 | -2.526411 | 0.538283 |
| 15 | 6 | C | 0.585790 | 2.596539 | 0.252775 |
| 16 | 6 | C | -0.740597 | 2.899896 | 0.393401 |
| 17 | 7 | N | -1.822412 | 2.032357 | 0.242375 |
| 18 | 7 | N | -3.824785 | -0.055891 | -0.044148 |
| 19 | 7 | N | -1.734095 | -2.064402 | 0.088592 |
| 20 | 6 | C | 0.355760 | 0.022488 | 0.200219 |
| 21 | 6 | C | 1.082074 | 1.237632 | 0.073153 |
| 22 | 6 | C | 2.461960 | 1.178692 | -0.204673 |
| 23 | 6 | C | 1.122385 | -1.169917 | 0.268617 |
| 24 | 6 | C | 2.511839 | -1.104261 | 0.051935 |
| 25 | 7 | N | 3.143797 | 0.033836 | -0.237619 |
| 26 | 79 | Au | -1.713384 | -0.012122 | 0.140584 |
| 27 | 1 | H | 3.116585 | -1.998329 | 0.101088 |
| 28 | 1 | H | 3.016175 | 2.084297 | -0.405067 |
| 29 | 1 | H | -0.633598 | 5.088183 | 0.858104 |
| 30 | 1 | H | -3.315385 | 4.937928 | 0.845880 |
| 31 | 1 | H | -6.819328 | 1.278506 | -0.646334 |
| 32 | 1 | H | -6.729714 | -1.379232 | -1.011311 |
| 33 | 1 | H | -3.162132 | -5.056961 | 0.270701 |
| 34 | 1 | H | -0.546509 | -5.080807 | 0.887528 |
| 35 | 6 | C | 1.567443 | 3.730925 | 0.341433 |
| 36 | 6 | C | 1.826785 | 4.526911 | -0.797393 |
| 37 | 6 | C | 2.223593 | 3.996080 | 1.565729 |
| 38 | 6 | C | 2.750796 | 5.571451 | -0.688751 |
| 39 | 6 | C | 1.146580 | 4.264663 | -2.123003 |
| 40 | 6 | C | 3.130743 | 5.058190 | 1.624370 |
| 41 | 6 | C | 1.951312 | 3.174023 | 2.805910 |
| 42 | 6 | C | 3.410938 | 5.857344 | 0.510596 |
| 43 | 1 | H | 2.961376 | 6.174547 | -1.568405 |
| 44 | 1 | H | 3.632281 | 5.264961 | 2.566633 |
| 45 | 6 | C | 4.378945 | 7.011336 | 0.607993 |
| 46 | 6 | C | -5.382772 | 3.388667 | 0.213359 |
| 47 | 6 | C | -6.373003 | 3.338278 | 1.206086 |
| 48 | 6 | C | -5.441925 | 4.419043 | -0.739434 |
| 49 | 6 | C | -7.388022 | 4.294043 | 1.245466 |
| 50 | 6 | C | -6.461556 | 5.366971 | -0.695433 |
| 51 | 6 | C | -7.453673 | 5.322540 | 0.296026 |
| 52 | 1 | H | -8.137836 | 4.241571 | 2.030262 |
| 53 | 1 | H | -6.489895 | 6.153068 | -1.445318 |
| 54 | 6 | C | -5.148978 | -3.565586 | -0.637808 |
| 55 | 6 | C | -6.259810 | -3.769016 | 0.195582 |
| 56 | 6 | C | -4.976485 | -4.416390 | -1.742034 |
| 57 | 6 | C | -7.171381 | -4.789351 | -0.073461 |
| 58 | 6 | C | -5.894462 | -5.430267 | -2.006176 |
| 59 | 6 | C | -7.009693 | -5.634749 | -1.179603 |
| 60 | 1 | H | -8.018273 | -4.934302 | 0.591783 |
| 61 | 1 | H | -5.743495 | -6.072888 | -2.869489 |
| 62 | 6 | C | 1.643550 | -3.587867 | 0.918788 |
| 63 | 6 | C | 2.065728 | -3.681358 | 2.265435 |
| 64 | 6 | C | 2.145987 | -4.481513 | -0.053250 |
| 65 | 6 | C | 2.983009 | -4.675681 | 2.613147 |
| 66 | 6 | C | 1.533325 | -2.745485 | 3.327482 |
| 67 | 6 | C | 3.069478 | -5.454897 | 0.345498 |
| 68 | 6 | C | 1.725227 | -4.401529 | -1.504496 |
| 69 | 6 | C | 3.498266 | -5.573058 | 1.670540 |
| 70 | 1 | H | 3.305604 | -4.748859 | 3.648817 |
| 71 | 1 | H | 3.466154 | -6.135113 | -0.403819 |
| 72 | 6 | C | 4.475537 | -6.647128 | 2.082280 |
| 73 | 1 | H | 4.852121 | 7.217480 | -0.355919 |
| 74 | 1 | H | 3.863707 | 7.927449 | 0.922204 |
| 75 | 1 | H | 5.164717 | 6.812322 | 1.342093 |
| 76 | 1 | H | 2.516572 | 3.561297 | 3.656262 |
| 77 | 1 | H | 0.889354 | 3.187382 | 3.074501 |
| 78 | 1 | H | 2.235124 | 2.123790 | 2.674194 |
| 79 | 1 | H | 1.302499 | 3.236246 | -2.467098 |
| 80 | 1 | H | 0.064104 | 4.422193 | -2.061877 |
| 81 | 1 | H | 1.536965 | 4.933357 | -2.893151 |
| 82 | 1 | H | 2.323954 | -5.080423 | -2.115628 |
| 83 | 1 | H | 0.672781 | -4.675040 | -1.638857 |
| 84 | 1 | H | 1.846578 | -3.392254 | -1.913530 |
| 85 | 1 | H | 1.790885 | -1.700878 | 3.118829 |
| 86 | 1 | H | 0.441482 | -2.799450 | 3.402343 |
| 87 | 1 | H | 1.948538 | -2.996582 | 4.305907 |
| 88 | 1 | H | 5.229638 | -6.255106 | 2.771602 |
| 89 | 1 | H | 3.961188 | -7.466715 | 2.598634 |
| 90 | 1 | H | 4.990161 | -7.072521 | 1.216970 |
| 91 | 6 | C | -8.568178 | 6.340034 | 0.321039 |
| 92 | 1 | H | -8.207231 | 7.333743 | 0.039370 |
| 93 | 1 | H | -9.359482 | 6.070439 | -0.389112 |
| 94 | 1 | H | -9.025561 | 6.408363 | 1.311599 |
| 95 | 6 | C | -8.013471 | -6.719099 | -1.486797 |
| 96 | 1 | H | -8.748666 | -6.372894 | -2.223624 |
| 97 | 1 | H | -7.528067 | -7.605010 | -1.906597 |
| 98 | 1 | H | -8.564183 | -7.019996 | -0.591538 |
| 99 | 1 | H | -6.400172 | -3.137799 | 1.068174 |
| 100 | 1 | H | -4.124243 | -4.276346 | -2.400908 |
| 101 | 1 | H | -4.691881 | 4.470771 | -1.523575 |
| 102 | 1 | H | -6.339284 | 2.556250 | 1.959087 |
| 103 | 79 | Au | 5.186539 | 0.020130 | -0.733668 |
| 104 | 17 | Cl | 7.464912 | -0.001420 | -1.303664 |
| 105 | 17 | Cl | 4.660302 | 1.224862 | -2.728198 |
| 106 | 17 | Cl | 5.643579 | -1.182887 | 1.275493 |

**Coordinates for compound 5**

| | Atomic Number | | Coordinates (Angstroms) X | Y | Z |
|---|---|---|---|---|---|
| 1 | 6 | C | 1.336836 | -4.270139 | 0.112013 |
| 2 | 6 | C | 2.663754 | -4.199065 | -0.190692 |
| 3 | 6 | C | 3.018321 | -2.808247 | -0.292875 |
| 4 | 6 | C | 4.334474 | -2.344493 | -0.458969 |
| 5 | 6 | C | 4.718430 | -1.007699 | -0.292068 |
| 6 | 6 | C | 6.071513 | -0.510527 | -0.342493 |
| 7 | 6 | C | 6.029302 | 0.813628 | -0.026972 |
| 8 | 6 | C | 4.645557 | 1.163582 | 0.178982 |
| 9 | 6 | C | 4.171443 | 2.456593 | 0.439043 |
| 10 | 6 | C | 2.817272 | 2.825006 | 0.384548 |
| 11 | 6 | C | 2.330178 | 4.163578 | 0.560954 |
| 12 | 6 | C | 0.997683 | 4.163438 | 0.268145 |
| 13 | 6 | C | 0.620486 | 2.815777 | -0.050198 |
| 14 | 6 | C | -0.675455 | 2.408694 | -0.421919 |
| 15 | 6 | C | -0.497363 | -2.572748 | 0.384472 |
| 16 | 6 | C | 0.833081 | -2.926793 | 0.161832 |
| 17 | 7 | N | 1.873382 | -2.057843 | -0.089572 |
| 18 | 7 | N | 3.874987 | 0.038085 | 0.008979 |
| 19 | 7 | N | 1.738305 | 2.020669 | 0.042820 |
| 20 | 6 | C | -0.295982 | -0.070026 | -0.160008 |
| 21 | 6 | C | -0.993325 | -1.261968 | 0.178587 |
| 22 | 6 | C | -2.427707 | -1.125316 | 0.399901 |
| 23 | 6 | C | -1.055384 | 1.060925 | -0.552579 |
| 24 | 6 | C | -2.411869 | 0.656473 | -1.165409 |
| 25 | 7 | N | -3.122804 | -0.206417 | -0.172186 |
| 26 | 79 | Au | 1.755940 | -0.015939 | -0.037434 |
| 27 | 8 | O | -3.268386 | 1.627186 | -1.646592 |
| 28 | 1 | H | -2.936062 | -1.831032 | 1.048026 |
| 29 | 1 | H | 0.737466 | -5.154162 | 0.270149 |
| 30 | 1 | H | 3.359790 | -5.015595 | -0.305958 |
| 31 | 1 | H | 6.940508 | -1.105919 | -0.577185 |
| 32 | 1 | H | 6.855006 | 1.506704 | 0.029043 |
| 33 | 1 | H | 2.940826 | 5.006067 | 0.846042 |
| 34 | 1 | H | 0.322464 | 5.005025 | 0.280134 |
| 35 | 6 | C | -1.428179 | -3.680686 | 0.800576 |
| 36 | 6 | C | -2.215015 | -4.358394 | -0.156628 |
| 37 | 6 | C | -1.501111 | -4.046204 | 2.164810 |
| 38 | 6 | C | -3.061236 | -5.388798 | 0.270896 |
| 39 | 6 | C | -2.176365 | -3.992575 | -1.624180 |
| 40 | 6 | C | -2.368400 | -5.074881 | 2.545064 |
| 41 | 6 | C | -0.671564 | -3.340563 | 3.214997 |
| 42 | 6 | C | -3.155229 | -5.762331 | 1.614312 |
| 43 | 1 | H | -3.664116 | -5.910033 | -0.469072 |
| 44 | 1 | H | -2.430491 | -5.345347 | 3.596683 |
| 45 | 6 | C | -4.057959 | -6.893150 | 2.047023 |
| 46 | 6 | C | 5.392311 | -3.356670 | -0.768085 |
| 47 | 6 | C | 6.401464 | -3.664041 | 0.157520 |
| 48 | 6 | C | 5.390697 | -4.034754 | -1.997001 |
| 49 | 6 | C | 7.377964 | -4.613705 | -0.141417 |
| 50 | 6 | C | 6.371391 | -4.981366 | -2.290788 |
| 51 | 6 | C | 7.384711 | -5.286434 | -1.371242 |
| 52 | 1 | H | 8.143200 | -4.840753 | 0.596597 |
| 53 | 1 | H | 6.349126 | -5.490674 | -3.250976 |
| 54 | 6 | C | 5.185436 | 3.515446 | 0.738656 |
| 55 | 6 | C | 5.954695 | 3.450067 | 1.910766 |
| 56 | 6 | C | 5.406801 | 4.586806 | -0.140841 |
| 57 | 6 | C | 6.906270 | 4.428863 | 2.195311 |
| 58 | 6 | C | 6.362804 | 5.559911 | 0.147416 |
| 59 | 6 | C | 7.130581 | 5.499685 | 1.318986 |
| 60 | 1 | H | 7.480740 | 4.361334 | 3.115762 |
| 61 | 1 | H | 6.516666 | 6.378265 | -0.551449 |
| 62 | 6 | C | -1.633514 | 3.563723 | -0.604252 |
| 63 | 6 | C | -2.368844 | 4.042907 | 0.504400 |
| 64 | 6 | C | -1.712915 | 4.232624 | -1.849972 |
| 65 | 6 | C | -3.199410 | 5.159994 | 0.332366 |
| 66 | 6 | C | -2.245083 | 3.409361 | 1.872395 |
| 67 | 6 | C | -2.550152 | 5.343584 | -1.969287 |
| 68 | 6 | C | -0.936400 | 3.741075 | -3.048205 |
| 69 | 6 | C | -3.309669 | 5.820363 | -0.893283 |
| 70 | 1 | H | -3.765333 | 5.524278 | 1.186148 |
| 71 | 1 | H | -2.613836 | 5.849537 | -2.929881 |

| | | | | | |
|---|---|---|---|---|---|
| 72 | 6 | C | -4.233960 | 7.002083 | -1.063177 |
| 73 | 1 | H | -4.894838 | -7.023446 | 1.355194 |
| 74 | 1 | H | -3.509918 | -7.843130 | 2.080482 |
| 75 | 1 | H | -4.464452 | -6.718647 | 3.047660 |
| 76 | 1 | H | -0.925670 | -3.700617 | 4.214878 |
| 77 | 1 | H | 0.400004 | -3.508358 | 3.059707 |
| 78 | 1 | H | -0.828355 | -2.256503 | 3.198091 |
| 79 | 1 | H | -2.770551 | -3.093698 | -1.829361 |
| 80 | 1 | H | -1.157268 | -3.797659 | -1.970295 |
| 81 | 1 | H | -2.594679 | -4.798812 | -2.232133 |
| 82 | 1 | H | -1.058746 | 4.418931 | -3.896445 |
| 83 | 1 | H | 0.133394 | 3.655423 | -2.831349 |
| 84 | 1 | H | -1.286457 | 2.749775 | -3.356648 |
| 85 | 1 | H | -2.506966 | 2.347702 | 1.861122 |
| 86 | 1 | H | -1.220339 | 3.488636 | 2.252351 |
| 87 | 1 | H | -2.908247 | 3.903823 | 2.585852 |
| 88 | 1 | H | -4.464191 | 7.471364 | -0.102760 |
| 89 | 1 | H | -3.796257 | 7.759853 | -1.720432 |
| 90 | 1 | H | -5.184829 | 6.693124 | -1.514229 |
| 91 | 6 | C | 8.462123 | -6.290872 | -1.704050 |
| 92 | 1 | H | 8.091353 | -7.065489 | -2.381657 |
| 93 | 1 | H | 9.312596 | -5.805886 | -2.199350 |
| 94 | 1 | H | 8.846101 | -6.778614 | -0.803356 |
| 95 | 6 | C | 8.184334 | 6.540431 | 1.613813 |
| 96 | 1 | H | 9.154907 | 6.247345 | 1.194359 |
| 97 | 1 | H | 7.922380 | 7.509292 | 1.178719 |
| 98 | 1 | H | 8.323517 | 6.674646 | 2.690471 |
| 99 | 1 | H | 5.795997 | 2.632882 | 2.608488 |
| 100 | 1 | H | 4.830542 | 4.653128 | -1.059016 |
| 101 | 1 | H | 4.617208 | -3.814969 | -2.727277 |
| 102 | 1 | H | 6.411886 | -3.165765 | 1.122536 |
| 103 | 1 | H | -3.268967 | 2.395738 | -1.052012 |
| 104 | 1 | H | -2.209305 | -0.006195 | -2.016552 |
| 105 | 79 | Au | -5.192615 | -0.113402 | -0.049649 |
| 106 | 17 | Cl | -7.560218 | -0.041532 | 0.062239 |
| 107 | 17 | Cl | -5.290324 | -1.206903 | -2.180418 |
| 108 | 17 | Cl | -5.022128 | 0.842805 | 2.131930 |

**Coordinates for compound 6**

| | Atomic Number | | Coordinates (Angstroms) | | |
|---|---|---|---|---|---|
| | | | X | Y | Z |
| 1 | 6 | C | 3.728272 | -2.133403 | 0.033965 |
| 2 | 6 | C | 4.288965 | -0.919319 | -0.237985 |
| 3 | 6 | C | 3.228796 | 0.050047 | -0.309138 |
| 4 | 6 | C | 3.430891 | 1.432670 | -0.447842 |
| 5 | 6 | C | 2.427276 | 2.400313 | -0.280286 |
| 6 | 6 | C | 2.617596 | 3.828543 | -0.318794 |
| 7 | 6 | C | 1.420806 | 4.406624 | -0.011435 |
| 8 | 6 | C | 0.466319 | 3.344518 | 0.181037 |
| 9 | 6 | C | -0.901503 | 3.517107 | 0.444995 |
| 10 | 6 | C | -1.856534 | 2.487892 | 0.396993 |
| 11 | 6 | C | -3.263725 | 2.665381 | 0.614278 |
| 12 | 6 | C | -3.882827 | 1.480728 | 0.329996 |
| 13 | 6 | C | -2.869415 | 0.534493 | -0.033178 |
| 14 | 6 | C | -3.105018 | -0.801586 | -0.425139 |
| 15 | 6 | C | 1.364111 | -2.964903 | 0.310168 |
| 16 | 6 | C | 2.306596 | -1.950458 | 0.098863 |
| 17 | 7 | N | 2.029138 | -0.618736 | -0.111601 |
| 18 | 7 | N | 1.107192 | 2.140660 | 0.005954 |
| 19 | 7 | N | -1.648585 | 1.162207 | 0.031045 |
| 20 | 6 | C | -0.729720 | -1.603550 | -0.193800 |
| 21 | 6 | C | -0.021049 | -2.785697 | 0.129062 |
| 22 | 6 | C | -0.881561 | -3.972468 | 0.375066 |
| 23 | 6 | C | -2.084595 | -1.745072 | -0.588718 |
| 24 | 6 | C | -2.332169 | -3.146186 | -1.187437 |
| 25 | 7 | N | -2.011895 | -4.167740 | -0.188513 |
| 26 | 79 | Au | 0.169149 | 0.232756 | -0.057657 |
| 27 | 8 | O | -3.569928 | -3.400865 | -1.786728 |
| 28 | 1 | H | -0.531855 | -4.705978 | 1.101092 |
| 29 | 1 | H | 4.229278 | -3.081239 | 0.164210 |
| 30 | 1 | H | 5.336984 | -0.686989 | -0.350377 |
| 31 | 1 | H | 3.551215 | 4.323786 | -0.538606 |
| 32 | 1 | H | 1.193064 | 5.460353 | 0.047832 |
| 33 | 1 | H | -3.725691 | 3.591026 | 0.921356 |
| 34 | 1 | H | -4.939031 | 1.262420 | 0.373257 |
| 35 | 6 | C | 1.906556 | -4.317746 | 0.689376 |
| 36 | 6 | C | 2.121047 | -5.306391 | -0.295346 |
| 37 | 6 | C | 2.212378 | -4.593347 | 2.040683 |
| 38 | 6 | C | 2.638230 | -6.548905 | 0.089804 |
| 39 | 6 | C | 1.800882 | -5.052316 | -1.751717 |
| 40 | 6 | C | 2.719496 | -5.852010 | 2.381702 |
| 41 | 6 | C | 1.990408 | -3.557905 | 3.120945 |
| 42 | 6 | C | 2.944746 | -6.843547 | 1.421504 |
| 43 | 1 | H | 2.803216 | -7.306412 | -0.673360 |
| 44 | 1 | H | 2.943839 | -6.061595 | 3.425393 |
| 45 | 6 | C | 3.526923 | -8.182835 | 1.808593 |
| 46 | 6 | C | 4.822102 | 1.902426 | -0.739980 |
| 47 | 6 | C | 5.559558 | 2.632632 | 0.204789 |
| 48 | 6 | C | 5.429524 | 1.613510 | -1.971930 |
| 49 | 6 | C | 6.856291 | 3.061973 | -0.077323 |
| 50 | 6 | C | 6.725831 | 2.046434 | -2.249223 |
| 51 | 6 | C | 7.462289 | 2.781403 | -1.309747 |
| 52 | 1 | H | 7.408875 | 3.618142 | 0.675985 |
| 53 | 1 | H | 7.171801 | 1.810387 | -3.212314 |
| 54 | 6 | C | -1.370561 | 4.903551 | 0.757289 |
| 55 | 6 | C | -0.938551 | 5.556004 | 1.922644 |
| 56 | 6 | C | -2.238207 | 5.594595 | -0.103250 |
| 57 | 6 | C | -1.365708 | 6.850251 | 2.218186 |
| 58 | 6 | C | -2.659173 | 6.889951 | 0.195449 |
| 59 | 6 | C | -2.230748 | 7.542761 | 1.359837 |
| 60 | 1 | H | -1.024625 | 7.327965 | 3.133367 |
| 61 | 1 | H | -3.329456 | 7.403335 | -0.489588 |
| 62 | 6 | C | -4.566130 | -1.155733 | -0.557373 |
| 63 | 6 | C | -5.240527 | -1.743724 | 0.534473 |
| 64 | 6 | C | -5.278695 | -0.835464 | -1.735478 |
| 65 | 6 | C | -6.613915 | -2.009188 | 0.422037 |
| 66 | 6 | C | -4.521533 | -2.081725 | 1.823700 |
| 67 | 6 | C | -6.645704 | -1.113427 | -1.800629 |
| 68 | 6 | C | -4.572462 | -0.242672 | -2.931257 |
| 69 | 6 | C | -7.335004 | -1.703932 | -0.733893 |
| 70 | 1 | H | -7.129798 | -2.459091 | 1.267314 |
| 71 | 1 | H | -7.187086 | -0.867723 | -2.711850 |
| 72 | 6 | C | -8.809036 | -2.015987 | -0.843560 |
| 73 | 1 | H | 3.205075 | -8.972171 | 1.122869 |
| 74 | 1 | H | 4.623759 | -8.158870 | 1.785072 |
| 75 | 1 | H | 3.231437 | -8.469291 | 2.822409 |
| 76 | 1 | H | 2.205778 | -3.973832 | 4.108541 |
| 77 | 1 | H | 2.634046 | -2.683093 | 2.976232 |
| 78 | 1 | H | 0.957807 | -3.192058 | 3.125408 |
| 79 | 1 | H | 0.719949 | -4.981931 | -1.918595 |
| 80 | 1 | H | 2.240304 | -4.113983 | -2.104717 |
| 81 | 1 | H | 2.179770 | -5.863350 | -2.379003 |
| 82 | 1 | H | -5.279148 | -0.030414 | -3.737815 |
| 83 | 1 | H | -4.053634 | 0.687972 | -2.679380 |
| 84 | 1 | H | -3.819964 | -0.941521 | -3.312620 |
| 85 | 1 | H | -3.758149 | -2.852581 | 1.672614 |
| 86 | 1 | H | -4.011492 | -1.206352 | 2.239886 |
| 87 | 1 | H | -5.226800 | -2.450706 | 2.572790 |
| 88 | 1 | H | -9.247909 | -2.223506 | 0.136339 |
| 89 | 1 | H | -9.360820 | -1.184854 | -1.295049 |
| 90 | 1 | H | -8.981304 | -2.895958 | -1.475476 |
| 91 | 6 | C | 8.854599 | 3.274943 | -1.624576 |
| 92 | 1 | H | 9.374442 | 2.596987 | -2.308024 |
| 93 | 1 | H | 8.824232 | 4.260520 | -2.105946 |
| 94 | 1 | H | 9.457975 | 3.375459 | -0.717611 |
| 95 | 6 | C | -2.665777 | 8.956430 | 1.665355 |
| 96 | 1 | H | -1.974662 | 9.685697 | 1.224283 |
| 97 | 1 | H | -3.659812 | 9.165791 | 1.259236 |
| 98 | 1 | H | -2.689960 | 9.143322 | 2.742952 |
| 99 | 1 | H | -0.272050 | 5.038633 | 2.606653 |
| 100 | 1 | H | -2.579639 | 5.114004 | -1.015358 |
| 101 | 1 | H | 4.878878 | 1.047114 | -2.717531 |
| 102 | 1 | H | 5.117045 | 2.853990 | 1.171697 |
| 103 | 1 | H | -4.250343 | -3.405564 | -1.099036 |
| 104 | 1 | H | -1.613687 | -3.247502 | -2.014535 |

**Coordinates for compound 7**

| | Atomic Number | | Coordinates (Angstroms) | | |
|---|---|---|---|---|---|
| | | | X | Y | Z |
| 1 | 6 | C | 4.262448 | -0.240173 | 0.491470 |
| 2 | 6 | C | 4.246092 | 1.109812 | 0.475493 |
| 3 | 6 | C | 2.884564 | 1.497334 | 0.065197 |
| 4 | 6 | C | 2.461615 | 2.863857 | -0.088306 |
| 5 | 6 | C | 1.134401 | 3.259448 | -0.210538 |
| 6 | 6 | C | 0.671383 | 4.616119 | -0.427563 |
| 7 | 6 | C | -0.684815 | 4.614126 | -0.426480 |
| 8 | 6 | C | -1.143430 | 3.256043 | -0.208885 |
| 9 | 6 | C | -2.469206 | 2.856022 | -0.085226 |
| 10 | 6 | C | -2.887517 | 1.487884 | 0.066464 |
| 11 | 6 | C | -4.247875 | 1.095378 | 0.475744 |
| 12 | 6 | C | -4.259876 | -0.254680 | 0.489757 |
| 13 | 6 | C | -2.915732 | -0.687783 | 0.086724 |
| 14 | 6 | C | -2.570486 | -2.003393 | -0.133370 |
| 15 | 6 | C | 2.578964 | -1.995081 | -0.130198 |
| 16 | 6 | C | 2.919806 | -0.678197 | 0.088679 |
| 17 | 7 | N | 2.105873 | 0.432910 | -0.133920 |
| 18 | 7 | N | -0.003298 | 2.474115 | -0.119378 |
| 19 | 7 | N | -2.105292 | 0.426232 | -0.133933 |
| 20 | 6 | C | 0.004133 | -1.978637 | 0.001499 |
| 21 | 6 | C | 1.229539 | -2.458696 | -0.506240 |
| 22 | 6 | C | 1.142725 | -3.492528 | -1.461944 |
| 23 | 6 | C | -1.219111 | -2.462416 | -0.507990 |
| 24 | 6 | C | -1.127781 | -3.495732 | -1.463856 |
| 25 | 7 | N | 0.008594 | -4.005772 | -1.938991 |
| 26 | 1 | H | -2.044060 | -3.920060 | -1.862620 |
| 27 | 1 | H | 2.060893 | -3.914352 | -1.859010 |
| 28 | 1 | H | 5.080279 | -0.894841 | 0.756947 |
| 29 | 1 | H | 5.047162 | 1.788624 | 0.729681 |
| 30 | 1 | H | 1.323510 | 5.462703 | -0.575440 |
| 31 | 1 | H | -1.339715 | 5.458721 | -0.573365 |
| 32 | 1 | H | -5.051209 | 1.771211 | 0.730762 |
| 33 | 1 | H | -5.075627 | -0.912366 | 0.754124 |
| 34 | 6 | C | 3.677732 | -3.023203 | -0.143505 |
| 35 | 6 | C | 4.590978 | -3.060842 | -1.224376 |

| | | | | | |
|---|---|---|---|---|---|
| 36 | 6 | C | 3.797025 | -3.964472 | 0.905378 |
| 37 | 6 | C | 5.592389 | -4.039473 | -1.234452 |
| 38 | 6 | C | 4.515287 | -2.085907 | -2.380517 |
| 39 | 6 | C | 4.821450 | -4.914053 | 0.856309 |
| 40 | 6 | C | 2.874055 | -3.931613 | 2.099123 |
| 41 | 6 | C | 5.727286 | -4.975576 | -0.207044 |
| 42 | 1 | H | 6.283762 | -4.069215 | -2.073518 |
| 43 | 1 | H | 4.913681 | -5.624471 | 1.674793 |
| 44 | 6 | C | 6.798526 | -6.038841 | -0.254647 |
| 45 | 6 | C | 3.519141 | 3.919136 | -0.097365 |
| 46 | 6 | C | 3.544676 | 4.944557 | 0.864309 |
| 47 | 6 | C | 4.537045 | 3.905139 | -1.063955 |
| 48 | 6 | C | 4.540774 | 5.918133 | 0.850321 |
| 49 | 6 | C | 5.532795 | 4.881936 | -1.073323 |
| 50 | 6 | C | 5.554244 | 5.906850 | -0.119276 |
| 51 | 6 | C | -3.530458 | 3.907511 | -0.090788 |
| 52 | 6 | C | -3.557453 | 4.931748 | 0.869479 |
| 53 | 6 | C | -4.549646 | 3.891773 | -1.058649 |
| 54 | 6 | C | -4.557044 | 5.904355 | 0.855593 |
| 55 | 6 | C | -5.546340 | 4.864993 | -1.067874 |
| 56 | 6 | C | -5.571192 | 5.889340 | -0.110411 |
| 57 | 6 | C | -3.666523 | -3.034305 | -0.149400 |
| 58 | 6 | C | -3.779727 | -3.983058 | 0.893441 |
| 59 | 6 | C | -4.578354 | -3.072278 | -1.231548 |
| 60 | 6 | C | -4.798379 | -4.938514 | 0.838604 |
| 61 | 6 | C | -2.852156 | -3.956976 | 2.083740 |
| 62 | 6 | C | -5.573881 | -4.056809 | -1.247456 |
| 63 | 6 | C | -4.502672 | -2.096084 | -2.386639 |
| 64 | 6 | C | -5.707405 | -4.995564 | -0.222253 |
| 65 | 1 | H | -4.881515 | -5.659183 | 1.649077 |
| 66 | 1 | H | -6.259896 | -4.090835 | -2.090745 |
| 67 | 6 | C | -6.810422 | -6.026658 | -0.245883 |
| 68 | 1 | H | 6.406696 | -6.974829 | -0.671816 |
| 69 | 1 | H | 7.640950 | -5.730549 | -0.880284 |
| 70 | 1 | H | 7.180007 | -6.266762 | 0.745317 |
| 71 | 1 | H | 3.094901 | -4.755628 | 2.782415 |
| 72 | 1 | H | 2.980591 | -2.995231 | 2.658113 |
| 73 | 1 | H | 1.821213 | -3.998508 | 1.814174 |
| 74 | 1 | H | 3.487907 | -1.930185 | -2.722233 |
| 75 | 1 | H | 4.907713 | -1.101450 | -2.103248 |
| 76 | 1 | H | 5.102578 | -2.451369 | -3.226854 |
| 77 | 1 | H | -5.087570 | -2.462123 | -3.234385 |
| 78 | 1 | H | -4.898280 | -1.113007 | -2.109018 |
| 79 | 1 | H | -3.475115 | -1.937167 | -2.726353 |
| 80 | 1 | H | -1.800191 | -4.019222 | 1.794625 |
| 81 | 1 | H | -2.958749 | -3.024937 | 2.649847 |
| 82 | 1 | H | -3.068709 | -4.786364 | 2.761883 |
| 83 | 1 | H | -6.480251 | -6.975269 | 0.188196 |
| 84 | 1 | H | -7.677836 | -5.689955 | 0.335507 |
| 85 | 1 | H | -7.156303 | -6.219000 | -1.265418 |
| 86 | 8 | O | 0.002043 | -1.020355 | 0.995229 |
| 87 | 6 | C | 0.000695 | -1.380962 | 2.311850 |
| 88 | 8 | O | 0.002041 | -2.527465 | 2.698175 |
| 89 | 6 | C | -0.002417 | -0.148812 | 3.174065 |
| 90 | 1 | H | 0.878337 | 0.458522 | 2.948276 |
| 91 | 1 | H | -0.883069 | 0.457103 | 2.944203 |
| 92 | 1 | H | -0.004513 | -0.435685 | 4.224642 |
| 93 | 1 | H | -0.001913 | 1.458846 | -0.048586 |
| 94 | 1 | H | -2.792334 | 4.959120 | 1.639659 |
| 95 | 1 | H | -4.551588 | 6.684290 | 1.612883 |
| 96 | 1 | H | -6.314568 | 4.833178 | -1.836550 |
| 97 | 1 | H | -4.548876 | 3.116237 | -1.818923 |
| 98 | 1 | H | 2.781759 | 4.967364 | 1.636843 |
| 99 | 1 | H | 4.536014 | 6.695683 | 1.610404 |
| 100 | 1 | H | 6.303172 | 4.849378 | -1.839472 |
| 101 | 1 | H | 4.540866 | 3.127413 | -1.821956 |
| 102 | 6 | C | -6.670876 | 6.924099 | -0.106760 |
| 103 | 1 | H | -6.986237 | 7.175567 | -1.123900 |
| 104 | 1 | H | -7.557522 | 6.556497 | 0.424705 |
| 105 | 6 | C | 6.631352 | 6.965288 | -0.125011 |
| 106 | 1 | H | 6.202772 | 7.968773 | -0.226492 |
| 107 | 1 | H | 7.203358 | 6.955518 | 0.809802 |
| 108 | 1 | H | 7.333737 | 6.815649 | -0.949083 |
| 109 | 1 | H | -6.352102 | 7.844946 | 0.389680 |

**Coordinates for compound 8**

| | Atomic Number | | Coordinates (Angstroms) | | |
|---|---|---|---|---|---|
| | | | X | Y | Z |
| 1 | 6 | C | -4.200182 | -0.302547 | -0.648131 |
| 2 | 6 | C | -4.194425 | 1.047529 | -0.646356 |
| 3 | 6 | C | -2.867166 | 1.452389 | -0.147325 |
| 4 | 6 | C | -2.465226 | 2.827797 | 0.001514 |
| 5 | 6 | C | -1.144896 | 3.234168 | 0.144391 |
| 6 | 6 | C | -0.686276 | 4.595003 | 0.350630 |
| 7 | 6 | C | 0.669805 | 4.597152 | 0.350457 |
| 8 | 6 | C | 1.132682 | 3.237751 | 0.144192 |
| 9 | 6 | C | 2.454273 | 2.835452 | 0.001377 |
| 10 | 6 | C | 2.860726 | 1.461238 | -0.146004 |
| 11 | 6 | C | 4.189918 | 1.060324 | -0.643003 |
| 12 | 6 | C | 4.200075 | -0.289727 | -0.643747 |
| 13 | 6 | C | 2.892967 | -0.713013 | -0.129491 |
| 14 | 6 | C | 2.550371 | -2.013650 | 0.145661 |
| 15 | 6 | C | -2.545968 | -2.021552 | 0.142458 |
| 16 | 6 | C | -2.892378 | -0.721944 | -0.132459 |
| 17 | 7 | N | -2.099502 | 0.398946 | 0.127570 |
| 18 | 7 | N | -0.004874 | 2.452045 | 0.071891 |
| 19 | 7 | N | 2.096172 | 0.405467 | 0.128774 |
| 20 | 6 | C | 0.002139 | -1.971814 | 0.128750 |
| 21 | 6 | C | -1.219702 | -2.417216 | 0.649966 |
| 22 | 6 | C | -1.134197 | -3.359168 | 1.692315 |
| 23 | 6 | C | 1.224717 | -2.413288 | 0.651619 |
| 24 | 6 | C | 1.140833 | -3.355424 | 1.693931 |
| 25 | 7 | N | 0.003695 | -3.816760 | 2.220284 |
| 26 | 1 | H | 2.061396 | -3.750551 | 2.115285 |
| 27 | 1 | H | -2.054071 | -3.757301 | 2.112338 |
| 28 | 1 | H | -4.990514 | -0.967933 | -0.965589 |
| 29 | 1 | H | -4.979164 | 1.717652 | -0.966491 |
| 30 | 1 | H | -1.341452 | 5.440849 | 0.489448 |
| 31 | 1 | H | 1.322305 | 5.445064 | 0.489287 |
| 32 | 1 | H | 4.972913 | 1.732761 | -0.962535 |
| 33 | 1 | H | 4.993002 | -0.952752 | -0.959640 |
| 34 | 6 | C | -3.555520 | -3.118394 | -0.011990 |
| 35 | 6 | C | -4.643382 | -3.236882 | 0.883650 |
| 36 | 6 | C | -3.402461 | -4.056748 | -1.059802 |
| 37 | 6 | C | -5.553562 | -4.286322 | 0.707436 |
| 38 | 6 | C | -4.850949 | -2.275344 | 2.034826 |
| 39 | 6 | C | -4.346091 | -5.076639 | -1.204595 |
| 40 | 6 | C | -2.247616 | -3.971111 | -2.032415 |
| 41 | 6 | C | -5.427595 | -5.214696 | -0.328475 |
| 42 | 1 | H | -6.384478 | -4.378107 | 1.403230 |
| 43 | 1 | H | -4.233670 | -5.781122 | -2.025832 |
| 44 | 6 | C | -6.411353 | -6.350015 | -0.482957 |
| 45 | 6 | C | -3.534265 | 3.869093 | -0.030326 |
| 46 | 6 | C | -3.533556 | 4.898997 | -0.985274 |
| 47 | 6 | C | -4.596956 | 3.832274 | 0.889605 |
| 48 | 6 | C | -4.545951 | 5.858065 | -1.009604 |
| 49 | 6 | C | -5.606316 | 4.791665 | 0.860368 |
| 50 | 6 | C | -5.598887 | 5.826220 | -0.086569 |
| 51 | 6 | C | 3.520528 | 3.879601 | -0.031372 |
| 52 | 6 | C | 3.515649 | 4.910623 | -0.985068 |
| 53 | 6 | C | 4.580379 | 3.849167 | 0.892067 |
| 54 | 6 | C | 4.522373 | 5.875702 | -1.006208 |
| 55 | 6 | C | 5.584027 | 4.814632 | 0.866077 |
| 56 | 6 | C | 5.576118 | 5.846269 | -0.084017 |
| 57 | 6 | C | 3.564405 | -3.106618 | -0.006503 |
| 58 | 6 | C | 3.412840 | -4.051187 | -1.048933 |
| 59 | 6 | C | 4.651131 | -3.219956 | 0.891322 |
| 60 | 6 | C | 4.358134 | -5.070327 | -1.188075 |
| 61 | 6 | C | 2.253792 | -3.977816 | -2.017584 |
| 62 | 6 | C | 5.563063 | -4.268809 | 0.720851 |
| 63 | 6 | C | 4.851617 | -2.257635 | 2.043110 |
| 64 | 6 | C | 5.442750 | -5.198742 | -0.314390 |
| 65 | 1 | H | 4.242855 | -5.783802 | -2.001118 |
| 66 | 1 | H | 6.389284 | -4.360391 | 1.422225 |
| 67 | 6 | C | 6.462727 | -6.296855 | -0.499628 |
| 68 | 1 | H | -6.040369 | -7.261979 | 0.001135 |
| 69 | 1 | H | -7.375907 | -6.108579 | -0.027563 |
| 70 | 1 | H | -6.581042 | -6.590921 | -1.536783 |
| 71 | 1 | H | -2.466562 | -4.531486 | -2.945043 |
| 72 | 1 | H | -2.020042 | -2.938028 | -2.309637 |
| 73 | 1 | H | -1.333118 | -4.394957 | -1.599752 |
| 74 | 1 | H | -3.910757 | -2.018360 | 2.530982 |
| 75 | 1 | H | -5.296592 | -1.332700 | 1.698855 |
| 76 | 1 | H | -5.521268 | -2.711345 | 2.780165 |
| 77 | 1 | H | 5.521759 | -2.690946 | 2.790186 |
| 78 | 1 | H | 5.294619 | -1.313405 | 1.708104 |
| 79 | 1 | H | 3.909241 | -2.004011 | 2.536838 |
| 80 | 1 | H | 1.344466 | -4.408459 | -1.580724 |
| 81 | 1 | H | 2.016741 | -2.947335 | -2.296482 |
| 82 | 1 | H | 2.474354 | -4.538389 | -2.929706 |
| 83 | 1 | H | 5.997844 | -7.216709 | -0.867137 |
| 84 | 1 | H | 7.226705 | -6.006255 | -1.231502 |
| 85 | 1 | H | 6.978946 | -6.525127 | 0.437088 |
| 86 | 9 | F | 0.001621 | -1.192546 | -0.960069 |
| 87 | 1 | H | 2.720883 | 4.947840 | -1.724044 |
| 88 | 1 | H | 4.491544 | 6.660629 | -1.757744 |
| 89 | 1 | H | 6.383964 | 4.771178 | 1.601136 |
| 90 | 1 | H | 4.605730 | 3.068167 | 1.646325 |
| 91 | 1 | H | -2.741591 | 4.935661 | -1.727277 |
| 92 | 1 | H | -4.522101 | 6.638092 | -1.766468 |
| 93 | 1 | H | -6.412432 | 4.738928 | 1.588030 |
| 94 | 1 | H | -4.624200 | 3.046657 | 1.638982 |
| 95 | 6 | C | 6.682499 | 6.872982 | -0.126294 |
| 96 | 1 | H | 7.029016 | 7.128657 | 0.879691 |
| 97 | 1 | H | 7.550965 | 6.495699 | -0.680512 |
| 98 | 6 | C | -6.680159 | 6.880109 | -0.095552 |
| 99 | 1 | H | -6.468636 | 7.669192 | 0.636797 |
| 100 | 1 | H | -6.763964 | 7.358485 | -1.075391 |
| 101 | 1 | H | -7.655224 | 6.455302 | 0.161894 |
| 102 | 1 | H | 6.355849 | 7.792961 | -0.619141 |
| 103 | 1 | H | -0.003283 | 1.437642 | 0.002507 |

**Coordinates for compound 10**

| | Atomic Number | | Coordinates (Angstroms) X | Y | Z |
|---|---|---|---|---|---|
| 1 | 6 | C | 4.315043 | -0.303583 | 0.203091 |
| 2 | 6 | C | 4.289080 | 1.058566 | 0.167499 |
| 3 | 6 | C | 2.913439 | 1.475627 | 0.005425 |
| 4 | 6 | C | 2.439928 | 2.813799 | -0.031594 |
| 5 | 6 | C | 1.085368 | 3.189876 | -0.082092 |
| 6 | 6 | C | 0.665307 | 4.598169 | -0.205813 |
| 7 | 6 | C | -0.680824 | 4.595791 | -0.206541 |
| 8 | 6 | C | -1.096035 | 3.186022 | -0.083171 |
| 9 | 6 | C | -2.449346 | 2.805175 | -0.033804 |
| 10 | 6 | C | -2.918162 | 1.465409 | 0.003358 |
| 11 | 6 | C | -4.292415 | 1.043675 | 0.165274 |
| 12 | 6 | C | -4.313665 | -0.318507 | 0.201827 |
| 13 | 6 | C | -2.961344 | -0.801186 | 0.036697 |
| 14 | 6 | C | -2.574171 | -2.125965 | -0.085412 |
| 15 | 6 | C | 2.581922 | -2.116831 | -0.085811 |
| 16 | 6 | C | 2.964428 | -0.790782 | 0.037444 |
| 17 | 7 | N | 2.164407 | 0.347695 | -0.082619 |
| 18 | 7 | N | -0.003936 | 2.364298 | -0.034880 |
| 19 | 7 | N | -2.165222 | 0.339990 | -0.083971 |
| 20 | 6 | C | 0.003608 | -1.962913 | 0.049963 |
| 21 | 6 | C | 1.236730 | -2.639457 | -0.324939 |
| 22 | 6 | C | 1.139106 | -3.899855 | -0.941464 |
| 23 | 6 | C | -1.227118 | -2.643864 | -0.324737 |
| 24 | 6 | C | -1.125184 | -3.903829 | -0.941285 |
| 25 | 7 | N | 0.008091 | -4.538067 | -1.262939 |
| 26 | 1 | H | -2.039240 | -4.426973 | -1.209094 |
| 27 | 1 | H | 2.054988 | -4.419695 | -1.209423 |
| 28 | 1 | H | 5.182466 | -0.936558 | 0.314926 |
| 29 | 1 | H | 5.126164 | 1.732632 | 0.260903 |
| 30 | 1 | H | 1.328233 | 5.445301 | -0.298369 |
| 31 | 1 | H | -1.346636 | 5.440583 | -0.299797 |
| 32 | 1 | H | -5.131835 | 1.714914 | 0.258062 |
| 33 | 1 | H | -5.178900 | -0.954400 | 0.313933 |
| 34 | 6 | C | 3.699647 | -3.123409 | 0.008240 |
| 35 | 6 | C | 4.556181 | -3.359070 | -1.089221 |
| 36 | 6 | C | 3.884810 | -3.835257 | 1.214939 |
| 37 | 6 | C | 5.581454 | -4.303495 | -0.956967 |
| 38 | 6 | C | 4.389401 | -2.627155 | -2.403505 |
| 39 | 6 | C | 4.930095 | -4.759057 | 1.303271 |
| 40 | 6 | C | 2.989193 | -3.600823 | 2.410105 |
| 41 | 6 | C | 5.787874 | -5.013623 | 0.228270 |
| 42 | 1 | H | 6.234145 | -4.487654 | -1.807270 |
| 43 | 1 | H | 5.077368 | -5.293601 | 2.239042 |
| 44 | 6 | C | 6.884489 | -6.046251 | 0.337779 |
| 45 | 6 | C | 3.483715 | 3.888164 | 0.005487 |
| 46 | 6 | C | 3.594861 | 4.751936 | 1.106000 |
| 47 | 6 | C | 4.383114 | 4.062592 | -1.058688 |
| 48 | 6 | C | 4.565250 | 5.753368 | 1.137548 |
| 49 | 6 | C | 5.352815 | 5.063392 | -1.021907 |
| 50 | 6 | C | 5.464455 | 5.926378 | 0.077322 |
| 51 | 6 | C | -3.496948 | 3.875859 | 0.002097 |
| 52 | 6 | C | -3.612186 | 4.739496 | 1.102286 |
| 53 | 6 | C | -4.395988 | 4.046832 | -1.062952 |
| 54 | 6 | C | -4.586187 | 5.737460 | 1.132705 |
| 55 | 6 | C | -5.369292 | 5.044160 | -1.027305 |
| 56 | 6 | C | -5.485040 | 5.906991 | 0.071628 |
| 57 | 6 | C | -3.688458 | -3.136144 | 0.010020 |
| 58 | 6 | C | -3.865969 | -3.853298 | 1.214973 |
| 59 | 6 | C | -4.544161 | -3.376020 | -1.086971 |
| 60 | 6 | C | -4.904450 | -4.784494 | 1.302668 |
| 61 | 6 | C | -2.964304 | -3.622399 | 2.406295 |
| 62 | 6 | C | -5.562710 | -4.328093 | -0.955398 |
| 63 | 6 | C | -4.379526 | -2.645913 | -2.402542 |
| 64 | 6 | C | -5.766095 | -5.038013 | 0.230276 |
| 65 | 1 | H | -5.041527 | -5.328158 | 2.234761 |
| 66 | 1 | H | -6.210695 | -4.520237 | -1.807499 |
| 67 | 6 | C | -6.893353 | -6.034674 | 0.360003 |
| 68 | 1 | H | 6.505834 | -7.049593 | 0.106429 |
| 69 | 1 | H | 7.701398 | -5.838045 | -0.359045 |
| 70 | 1 | H | 7.298884 | -6.083258 | 1.349742 |
| 71 | 1 | H | 3.351316 | -4.153059 | 3.280739 |
| 72 | 1 | H | 2.939289 | -2.539706 | 2.675008 |
| 73 | 1 | H | 1.961553 | -3.923482 | 2.210377 |
| 74 | 1 | H | 3.341381 | -2.582892 | -2.714800 |
| 75 | 1 | H | 4.745476 | -1.592942 | -2.339526 |
| 76 | 1 | H | 4.957046 | -3.123924 | -3.194399 |
| 77 | 1 | H | -4.943312 | -3.147476 | -3.193183 |
| 78 | 1 | H | -4.742014 | -1.613773 | -2.340937 |
| 79 | 1 | H | -3.331443 | -2.595813 | -2.712679 |
| 80 | 1 | H | -1.939249 | -3.951280 | 2.203287 |
| 81 | 1 | H | -2.907420 | -2.561206 | 2.669272 |
| 82 | 1 | H | -3.326287 | -4.171251 | 3.279130 |
| 83 | 1 | H | -6.600981 | -6.890703 | 0.975731 |
| 84 | 1 | H | -7.770482 | -5.579093 | 0.836210 |
| 85 | 1 | H | -7.210248 | -6.409130 | -0.617379 |
| 86 | 8 | O | 0.001633 | -0.878194 | 0.714640 |
| 87 | 1 | H | -2.932493 | 4.624392 | 1.941488 |
| 88 | 1 | H | -4.650526 | 6.392714 | 1.997816 |
| 89 | 1 | H | -6.045097 | 5.159596 | -1.871127 |
| 90 | 1 | H | -4.320772 | 3.399983 | -1.932397 |
| 91 | 1 | H | 2.914831 | 4.634180 | 1.944565 |
| 92 | 1 | H | 4.626470 | 6.408603 | 2.002865 |
| 93 | 1 | H | 6.029007 | 5.181413 | -1.865070 |
| 94 | 1 | H | 4.311027 | 3.415684 | -1.928350 |
| 95 | 6 | C | -6.559756 | 6.966873 | 0.119316 |
| 96 | 1 | H | -6.772128 | 7.366073 | -0.876994 |
| 97 | 1 | H | -7.499764 | 6.559141 | 0.511641 |
| 98 | 6 | C | 6.535235 | 6.990178 | 0.126201 |
| 99 | 1 | H | 6.243160 | 7.822235 | 0.773169 |
| 100 | 1 | H | 7.476203 | 6.585942 | 0.519832 |
| 101 | 1 | H | 6.747447 | 7.389937 | -0.869919 |
| 102 | 1 | H | -6.271554 | 7.799846 | 0.766838 |
| 103 | 1 | H | -1.150404 | 0.286917 | 0.059515 |
| 104 | 1 | H | 1.149707 | 0.290976 | 0.060381 |

**Coordinates for compound 11**

| | Atomic Number | | Coordinates (Angstroms) X | Y | Z |
|---|---|---|---|---|---|
| 1 | 6 | C | -4.209861 | -0.800990 | -0.142993 |
| 2 | 6 | C | -4.310728 | 0.559649 | -0.155889 |
| 3 | 6 | C | -2.978312 | 1.101834 | -0.044781 |
| 4 | 6 | C | -2.688090 | 2.483985 | -0.006922 |
| 5 | 6 | C | -1.398584 | 3.043557 | 0.007789 |
| 6 | 6 | C | -1.091982 | 4.449756 | 0.164022 |
| 7 | 6 | C | 0.264417 | 4.573005 | 0.152331 |
| 8 | 6 | C | 0.817476 | 3.245120 | -0.010620 |
| 9 | 6 | C | 2.185487 | 2.926848 | -0.043796 |
| 10 | 6 | C | 2.721079 | 1.618905 | -0.074714 |
| 11 | 6 | C | 4.125332 | 1.322710 | -0.195165 |
| 12 | 6 | C | 4.269011 | -0.035738 | -0.165727 |
| 13 | 6 | C | 2.960225 | -0.608028 | -0.039670 |
| 14 | 6 | C | 2.677403 | -1.986438 | 0.005493 |
| 15 | 6 | C | -2.288729 | -2.430692 | -0.010891 |
| 16 | 6 | C | -2.816589 | -1.132173 | -0.036630 |
| 17 | 7 | N | -2.091366 | 0.044569 | 0.026054 |
| 18 | 7 | N | -0.219518 | 2.350508 | -0.098292 |
| 19 | 7 | N | 2.038473 | 0.419119 | 0.017091 |
| 20 | 6 | C | 0.157551 | -1.738199 | -0.050462 |
| 21 | 6 | C | -0.903818 | -2.655277 | 0.004049 |
| 22 | 6 | C | -0.226053 | -3.951263 | 0.056559 |
| 23 | 6 | C | 1.361912 | -2.460622 | 0.015492 |
| 24 | 7 | N | 1.077451 | -3.851614 | 0.063176 |
| 25 | 1 | H | -0.723520 | -4.916008 | 0.086808 |
| 26 | 1 | H | -5.007471 | -1.526716 | -0.212395 |
| 27 | 1 | H | -5.208518 | 1.152297 | -0.242596 |
| 28 | 1 | H | -1.823071 | 5.234744 | 0.286193 |
| 29 | 1 | H | 0.843663 | 5.477345 | 0.263316 |
| 30 | 1 | H | 4.902515 | 2.064221 | -0.299518 |
| 31 | 1 | H | 5.182429 | -0.608532 | -0.235771 |
| 32 | 6 | C | -3.231116 | -3.599997 | -0.010696 |
| 33 | 6 | C | -3.737301 | -4.097276 | 1.209460 |
| 34 | 6 | C | -3.603030 | -4.207162 | -1.229622 |
| 35 | 6 | C | -4.606299 | -5.194039 | 1.187187 |
| 36 | 6 | C | -3.358355 | -3.466366 | 2.530830 |
| 37 | 6 | C | -4.476626 | -5.300234 | -1.204466 |
| 38 | 6 | C | -3.081652 | -3.693155 | -2.553126 |
| 39 | 6 | C | -4.986798 | -5.812683 | -0.007620 |
| 40 | 1 | H | -4.995065 | -5.573371 | 2.129735 |
| 41 | 1 | H | -4.765166 | -5.761680 | -2.146416 |
| 42 | 6 | C | -5.900540 | -7.015904 | -0.003900 |
| 43 | 6 | C | -3.847194 | 3.431367 | 0.026545 |
| 44 | 6 | C | -4.086566 | 4.315730 | -1.036946 |
| 45 | 6 | C | -4.724289 | 3.464951 | 1.122342 |
| 46 | 6 | C | -5.165455 | 5.199046 | -1.004286 |
| 47 | 6 | C | -5.799810 | 4.351874 | 1.151042 |
| 48 | 6 | C | -6.040004 | 5.236744 | 0.090475 |
| 49 | 6 | C | 3.154025 | 4.068528 | -0.036875 |
| 50 | 6 | C | 3.202800 | 4.977212 | -1.105381 |
| 51 | 6 | C | 4.027743 | 4.272750 | 1.043340 |
| 52 | 6 | C | 4.095946 | 6.048636 | -1.093637 |
| 53 | 6 | C | 4.916059 | 5.347146 | 1.051352 |
| 54 | 6 | C | 4.970326 | 6.252973 | -0.017767 |
| 55 | 6 | C | 3.823735 | -2.954267 | 0.029856 |
| 56 | 6 | C | 4.264792 | -3.556703 | -1.166452 |
| 57 | 6 | C | 4.464881 | -3.254887 | 1.249599 |
| 58 | 6 | C | 5.348740 | -4.440294 | -1.122624 |
| 59 | 6 | C | 3.581645 | -3.269656 | -2.483916 |
| 60 | 6 | C | 5.541799 | -4.148703 | 1.248419 |
| 61 | 6 | C | 4.003129 | -2.638798 | 2.551320 |
| 62 | 6 | C | 6.000581 | -4.753024 | 0.074178 |
| 63 | 1 | H | 5.690449 | -4.896330 | -2.049493 |
| 64 | 1 | H | 6.033383 | -4.377672 | 2.191657 |
| 65 | 6 | C | 7.147415 | -5.736516 | 0.101689 |
| 66 | 1 | H | -5.325571 | -7.948119 | 0.062629 |
| 67 | 1 | H | -6.585015 | -6.996083 | 0.849433 |
| 68 | 1 | H | -6.496972 | -7.068337 | -0.919688 |
| 69 | 1 | H | -3.505990 | -4.260750 | -3.385216 |
| 70 | 1 | H | -3.331509 | -2.636863 | -2.700895 |
| 71 | 1 | H | -1.990626 | -3.771201 | -2.613607 |
| 72 | 1 | H | -2.272985 | -3.462523 | 2.678213 |
| 73 | 1 | H | -3.688854 | -2.423322 | 2.587724 |

| | | | | | |
|---|---|---|---|---|---|
| 74 | 1 | H | -3.810546 | -4.008970 | 3.364952 |
| 75 | 1 | H | 4.575824 | -3.034949 | 3.394000 |
| 76 | 1 | H | 4.119211 | -1.549290 | 2.547950 |
| 77 | 1 | H | 2.943229 | -2.845302 | 2.735225 |
| 78 | 1 | H | 2.554629 | -3.651300 | -2.481636 |
| 79 | 1 | H | 3.523654 | -2.194630 | -2.685875 |
| 80 | 1 | H | 4.116310 | -3.742020 | -3.312293 |
| 81 | 1 | H | 7.705551 | -5.727060 | -0.839481 |
| 82 | 1 | H | 7.845458 | -5.515253 | 0.914819 |
| 83 | 1 | H | 6.786219 | -6.761439 | 0.254105 |
| 84 | 1 | H | 2.537545 | 4.839428 | -1.952870 |
| 85 | 1 | H | 4.114329 | 6.735970 | -1.935795 |
| 86 | 1 | H | 5.573791 | 5.488034 | 1.905587 |
| 87 | 1 | H | 3.999872 | 3.590279 | 1.887838 |
| 88 | 1 | H | -3.428219 | 4.300764 | -1.900775 |
| 89 | 1 | H | -5.333704 | 5.865743 | -1.846525 |
| 90 | 1 | H | -6.462222 | 4.358066 | 2.013171 |
| 91 | 1 | H | -4.558401 | 2.792180 | 1.958814 |
| 92 | 6 | C | 5.956347 | 7.396990 | -0.019042 |
| 93 | 1 | H | 6.132900 | 7.773692 | 0.992976 |
| 94 | 1 | H | 6.927125 | 7.080492 | -0.420744 |
| 95 | 6 | C | -7.189051 | 6.215790 | 0.137661 |
| 96 | 1 | H | -6.883587 | 7.160167 | 0.605484 |
| 97 | 1 | H | -7.552958 | 6.454040 | -0.866122 |
| 98 | 1 | H | -8.026696 | 5.821734 | 0.720727 |
| 99 | 1 | H | 5.604224 | 8.228106 | -0.636896 |
| 100 | 47 | Ag | -0.028278 | 0.261834 | -0.034499 |

**Coordinates for compound 14**

| | Atomic Number | | Coordinates (Angstroms) X | Y | Z |
|---|---|---|---|---|---|
| 1 | 6 | C | -2.347601 | 3.517284 | -0.186631 |
| 2 | 6 | C | -1.145775 | 4.162837 | -0.195093 |
| 3 | 6 | C | -0.111692 | 3.170324 | -0.048587 |
| 4 | 6 | C | 1.263425 | 3.463361 | 0.008731 |
| 5 | 6 | C | 2.300081 | 2.509734 | 0.043853 |
| 6 | 6 | C | 3.699818 | 2.800136 | 0.233855 |
| 7 | 6 | C | 4.367042 | 1.608999 | 0.219692 |
| 8 | 6 | C | 3.387306 | 0.569649 | 0.021720 |
| 9 | 6 | C | 3.659693 | -0.812481 | -0.033592 |
| 10 | 6 | C | 2.689823 | -1.829047 | -0.077211 |
| 11 | 6 | C | 2.982932 | -3.230894 | -0.218400 |
| 12 | 6 | C | 1.797761 | -3.911062 | -0.189429 |
| 13 | 6 | C | 0.748873 | -2.943328 | -0.044273 |
| 14 | 6 | C | -0.627145 | -3.246154 | 0.006411 |
| 15 | 6 | C | -3.077204 | 1.110019 | -0.021679 |
| 16 | 6 | C | -2.086684 | 2.110865 | -0.048867 |
| 17 | 7 | N | -0.722648 | 1.927802 | 0.040329 |
| 18 | 7 | N | 2.147613 | 1.150433 | -0.082911 |
| 19 | 7 | N | 1.310327 | -1.692187 | 0.028717 |
| 20 | 6 | C | -1.467420 | -0.844081 | -0.071285 |
| 21 | 6 | C | -2.729614 | -0.245214 | -0.004352 |
| 22 | 6 | C | -3.729615 | -1.377861 | 0.071849 |
| 23 | 6 | C | -1.590952 | -2.243163 | 0.016666 |
| 24 | 7 | N | -2.965894 | -2.526699 | 0.088304 |
| 25 | 8 | O | -4.952771 | -1.343972 | 0.113568 |
| 26 | 1 | H | -3.333568 | 3.948069 | -0.280897 |
| 27 | 1 | H | -0.968790 | 5.222235 | -0.301721 |
| 28 | 1 | H | 4.116178 | 3.785623 | 0.378692 |
| 29 | 1 | H | 5.426592 | 1.447402 | 0.349870 |
| 30 | 1 | H | 3.973985 | -3.642006 | -0.335918 |
| 31 | 1 | H | 1.641397 | -4.977082 | -0.271202 |
| 32 | 6 | C | -4.511607 | 1.558075 | -0.028941 |
| 33 | 6 | C | -5.151388 | 1.891664 | 1.182397 |
| 34 | 6 | C | -5.205631 | 1.683170 | -1.249798 |
| 35 | 6 | C | -6.473101 | 2.349627 | 1.150065 |
| 36 | 6 | C | -4.438743 | 1.750848 | 2.508460 |
| 37 | 6 | C | -6.524916 | 2.148333 | -1.237345 |
| 38 | 6 | C | -4.551450 | 1.318767 | -2.563043 |
| 39 | 6 | C | -7.180051 | 2.483799 | -0.048538 |
| 40 | 1 | H | -6.961460 | 2.607785 | 2.087493 |
| 41 | 1 | H | -7.053614 | 2.250320 | -2.182871 |
| 42 | 6 | C | -8.616826 | 2.951586 | -0.056734 |
| 43 | 6 | C | 1.662196 | 4.905903 | 0.038141 |
| 44 | 6 | C | 2.408339 | 5.468905 | -1.009342 |
| 45 | 6 | C | 1.305977 | 5.734752 | 1.113862 |
| 46 | 6 | C | 2.780437 | 6.812538 | -0.981071 |
| 47 | 6 | C | 1.684473 | 7.076581 | 1.139069 |
| 48 | 6 | C | 2.429701 | 7.640670 | 0.094249 |
| 49 | 6 | C | 5.098137 | -1.225836 | -0.035729 |
| 50 | 6 | C | 5.948481 | -0.864268 | -1.092483 |
| 51 | 6 | C | 5.641212 | -1.973200 | 1.021717 |
| 52 | 6 | C | 7.290784 | -1.242705 | -1.092506 |
| 53 | 6 | C | 6.984958 | -2.345065 | 1.018642 |
| 54 | 6 | C | 7.834085 | -1.991438 | -0.039554 |
| 55 | 6 | C | -1.061816 | -4.683224 | 0.034010 |
| 56 | 6 | C | -1.398686 | -5.343046 | -1.168671 |
| 57 | 6 | C | -1.143174 | -5.372761 | 1.263925 |
| 58 | 6 | C | -1.806049 | -6.681105 | -1.118307 |
| 59 | 6 | C | -1.316091 | -4.631658 | -2.500895 |
| 60 | 6 | C | -1.557339 | -6.709381 | 1.267139 |
| 61 | 6 | C | -0.788072 | -4.692065 | 2.566822 |
| 62 | 6 | C | -1.895656 | -7.381959 | 0.088493 |
| 63 | 1 | H | -2.057322 | -7.187738 | -2.047550 |
| 64 | 1 | H | -1.616076 | -7.237680 | 2.216220 |
| 65 | 6 | C | -2.369840 | -8.815953 | 0.120996 |
| 66 | 1 | H | -9.309193 | 2.104273 | 0.026339 |
| 67 | 1 | H | -8.824599 | 3.622386 | 0.782539 |
| 68 | 1 | H | -8.861260 | 3.479031 | -0.983882 |
| 69 | 1 | H | -5.205261 | 1.561174 | -3.405030 |
| 70 | 1 | H | -3.603046 | 1.847582 | -2.706412 |
| 71 | 1 | H | -4.330244 | 0.246479 | -2.609271 |
| 72 | 1 | H | -4.139209 | 0.712447 | 2.689254 |
| 73 | 1 | H | -3.527064 | 2.357025 | 2.546718 |
| 74 | 1 | H | -5.085429 | 2.061514 | 3.333321 |
| 75 | 1 | H | -0.950962 | -5.363366 | 3.413810 |
| 76 | 1 | H | 0.261160 | -4.377301 | 2.581438 |
| 77 | 1 | H | -1.390950 | -3.791740 | 2.727956 |
| 78 | 1 | H | -2.012147 | -3.786750 | -2.550328 |
| 79 | 1 | H | -0.313757 | -4.227717 | -2.678586 |
| 80 | 1 | H | -1.558004 | -5.312555 | -3.320857 |
| 81 | 1 | H | -2.117235 | -9.341883 | -0.804473 |
| 82 | 1 | H | -1.928041 | -9.364542 | 0.958142 |
| 83 | 1 | H | -3.459638 | -8.868352 | 0.237371 |
| 84 | 1 | H | 5.551537 | -0.286258 | -1.921946 |
| 85 | 1 | H | 7.925945 | -0.952524 | -1.925840 |
| 86 | 1 | H | 7.381887 | -2.914344 | 1.855620 |
| 87 | 1 | H | 5.006536 | -2.252710 | 1.857560 |
| 88 | 1 | H | 2.687067 | 4.850207 | -1.857409 |
| 89 | 1 | H | 3.348042 | 7.225619 | -1.811326 |
| 90 | 1 | H | 1.397401 | 7.695090 | 1.985918 |
| 91 | 1 | H | 0.731542 | 5.321326 | 1.937733 |
| 92 | 6 | C | 9.280893 | -2.424454 | -0.054866 |
| 93 | 1 | H | 9.693281 | -2.477101 | 0.957219 |
| 94 | 1 | H | 9.389622 | -3.420883 | -0.501308 |
| 95 | 6 | C | 2.862338 | 9.087032 | 0.137754 |
| 96 | 1 | H | 3.837475 | 9.192380 | 0.629733 |
| 97 | 1 | H | 2.960092 | 9.505595 | -0.868261 |
| 98 | 1 | H | 2.149776 | 9.701239 | 0.696176 |
| 99 | 1 | H | 9.898168 | -1.737054 | -0.640519 |
| 100 | 47 | Ag | 0.310569 | 0.128161 | -0.031717 |
| 101 | 1 | H | -3.358920 | -3.456802 | 0.109787 |

**Correlations between calculated and experimental NMR values**

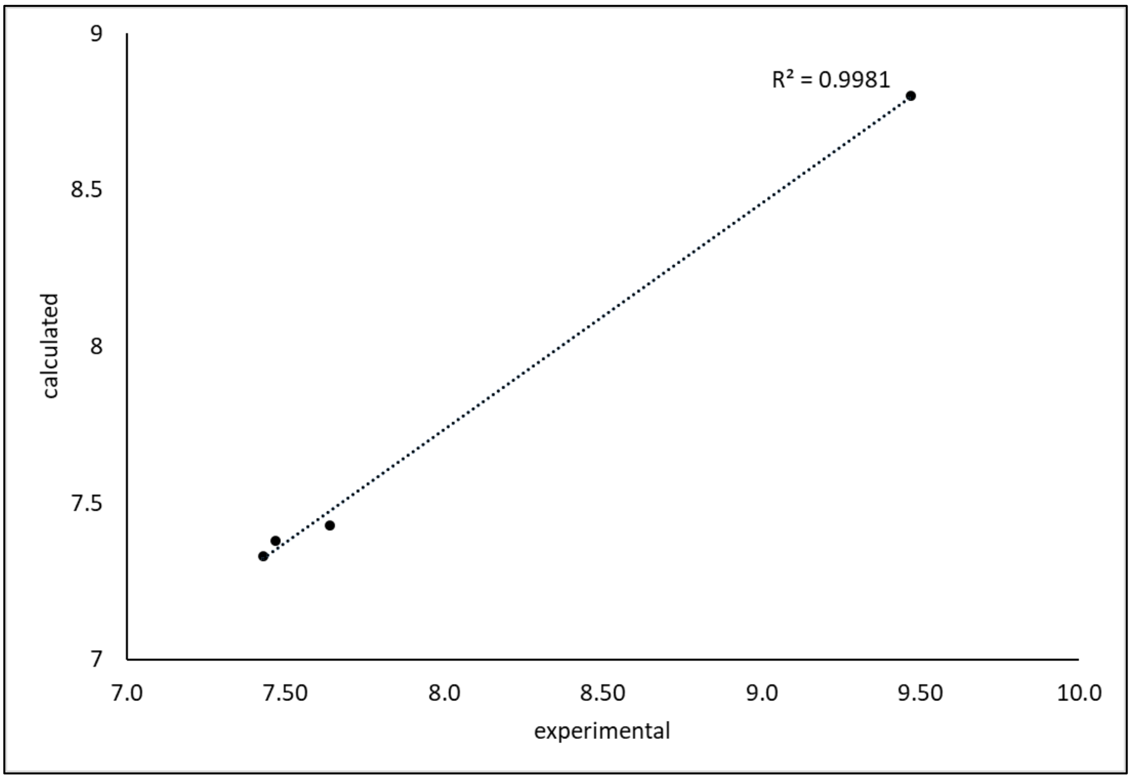


**Figure S67. The correlation between calculated and experimental NMR values for 4.**

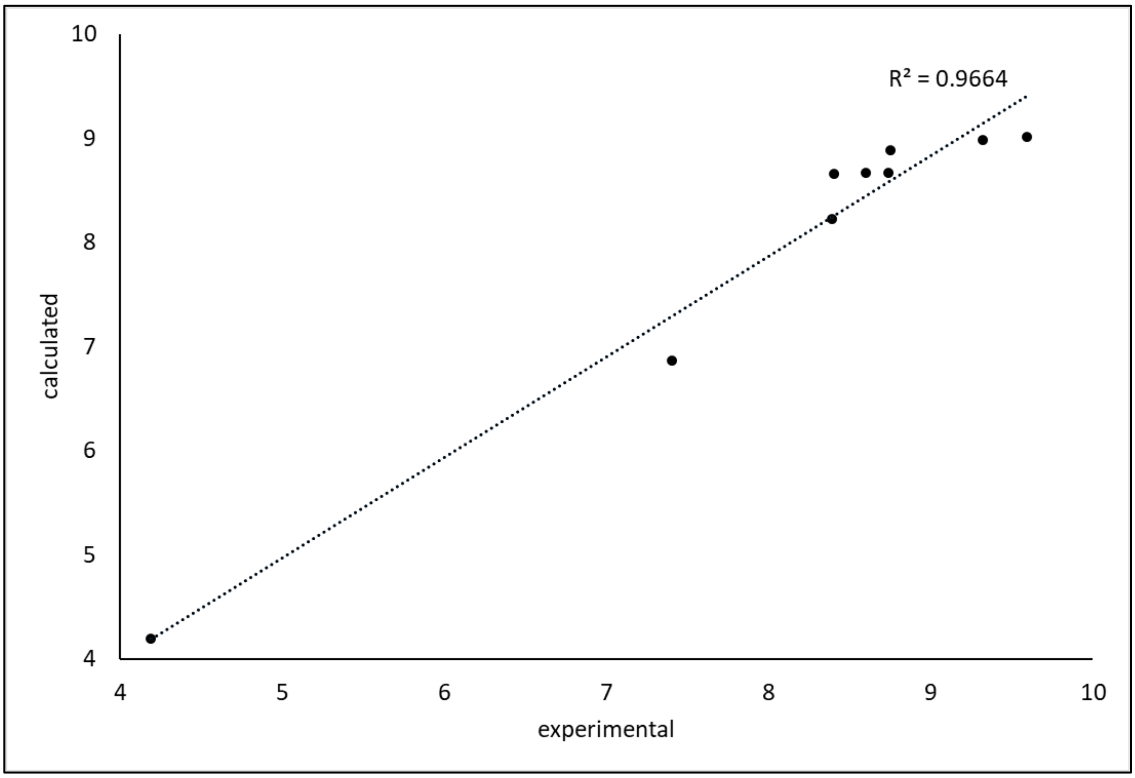


**Figure S68. The correlation between calculated and experimental NMR values for 5.**

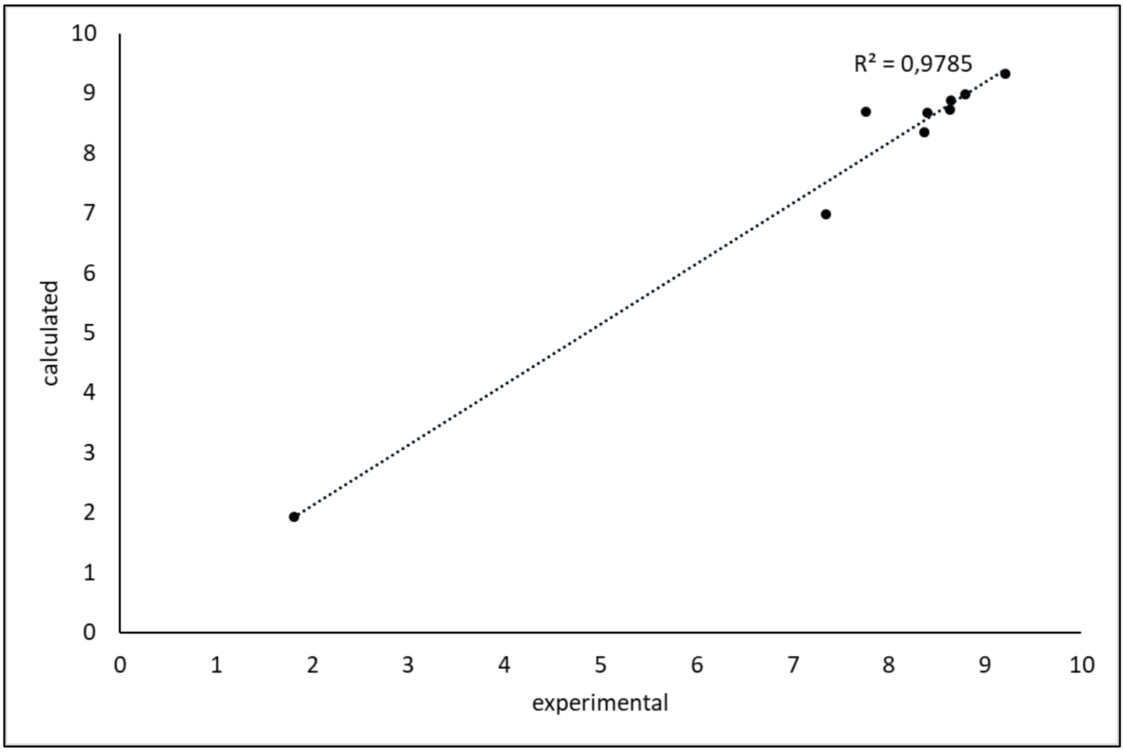


**Figure S69.The correlation between calculated and experimental NMR values for 6.**

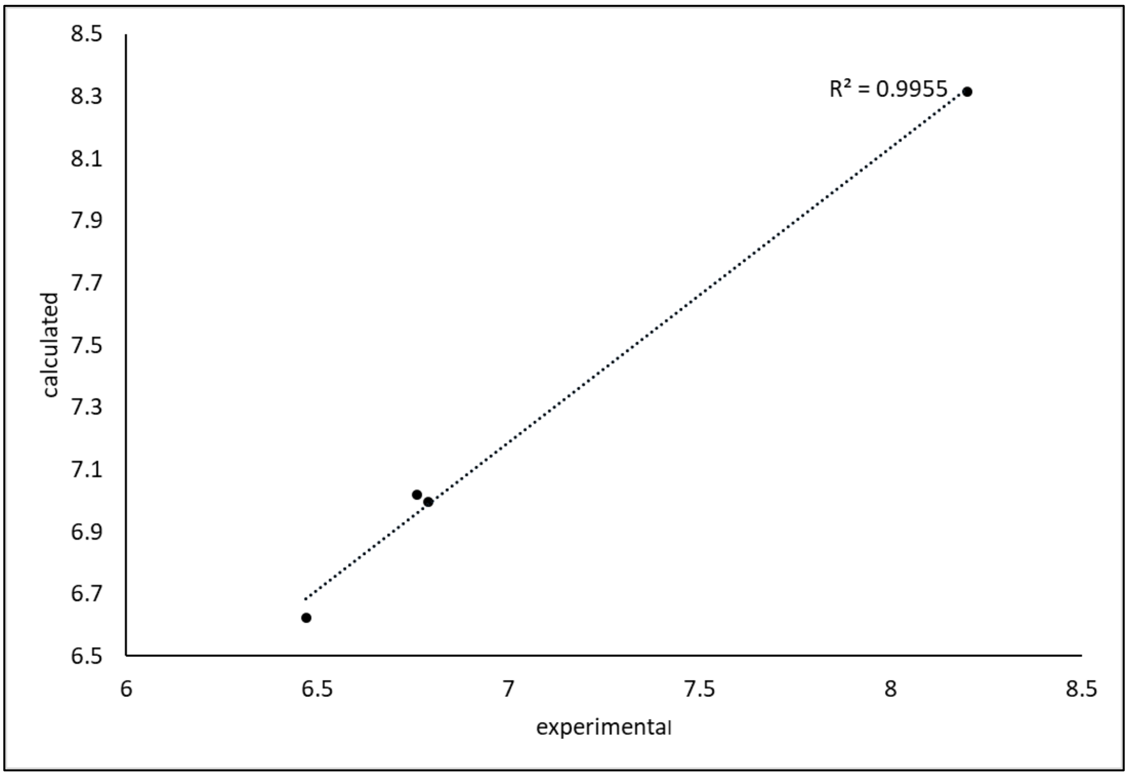


**Figure S70. The correlation between calculated and experimental NMR values for 7.**

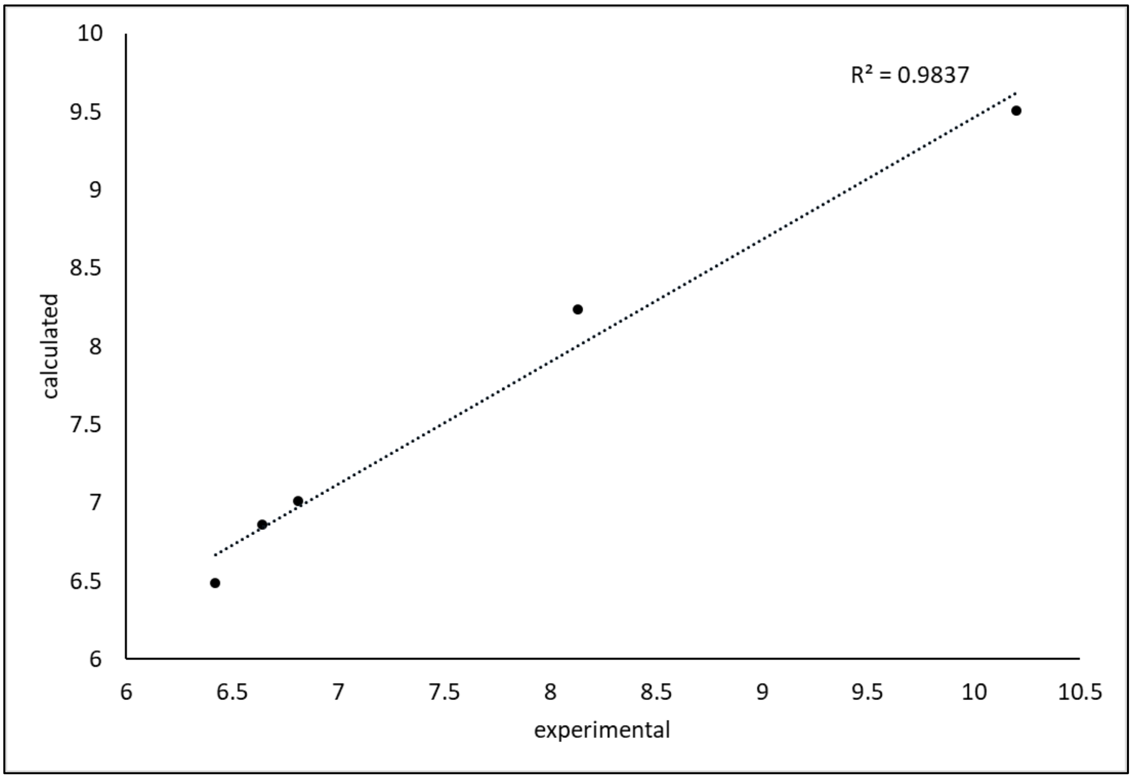


**Figure S71. The correlation between calculated and experimental NMR values for 8.**

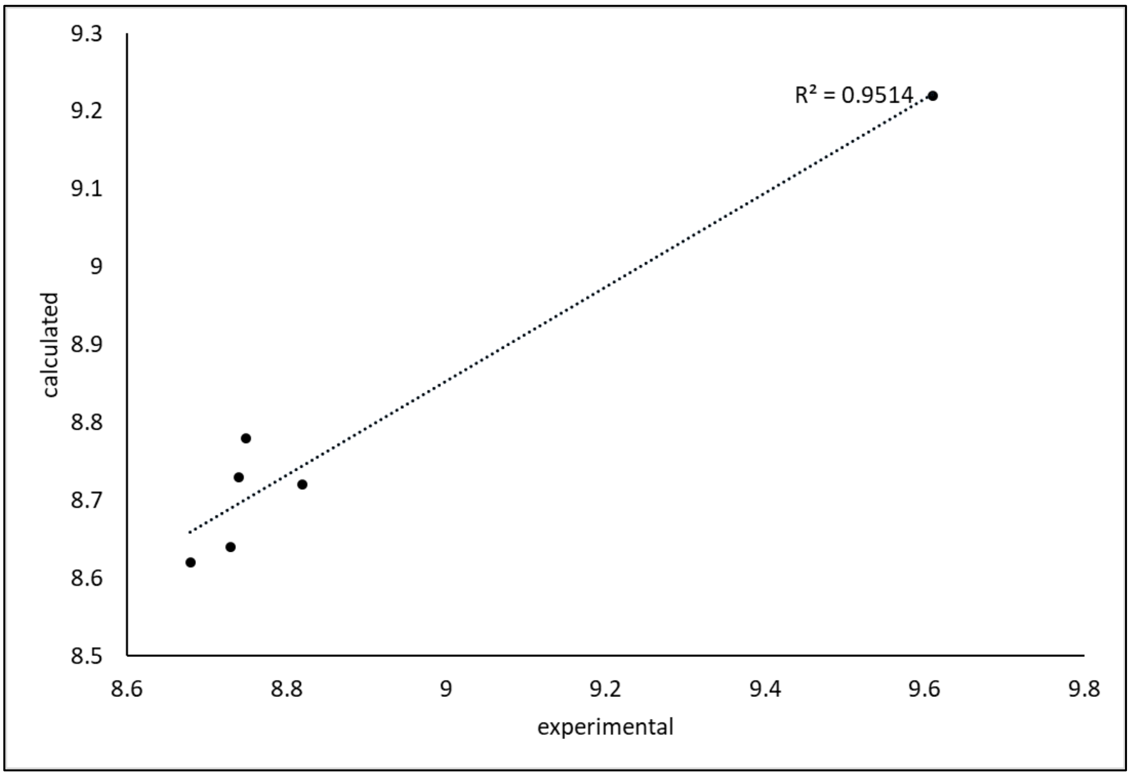


**Figure S72. The correlation between calculated and experimental NMR values for 10.**

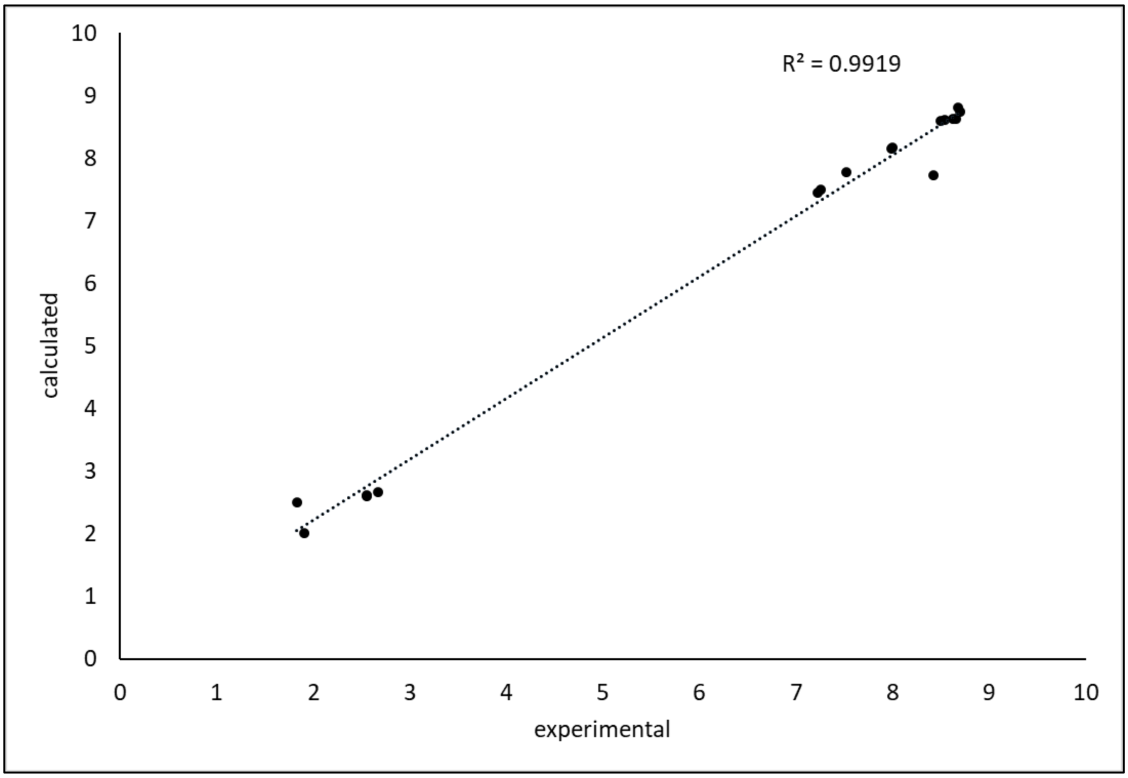


**Figure S73. The correlation between calculated and experimental NMR values for 11.**

**Bibliography**

[1] G. M. Sheldrick, *Acta Crystallogr. A* **2015** 71, 3–8, 11

[2] G. M. Sheldrick, *Acta Crystallogr. C* **2015** 71, 3–8.

[3] M. J. Frisch, G. W. Trucks, H. B. Schlegel, G. E. Scuseria, M. A. Robb, J. R. Cheeseman, G. Scalmani, V. Barone, G. A. Petersson, H. Nakatsuji, X. Li, M. Caricato, A. V. Marenich, J. Bloino, B. G. Janesko, R. Gomperts, B. Mennucci, H. P. Hratchian, J. V. Ortiz, A. F. Izmaylov, J. L. Sonnenberg F. Ding, F. Lipparini, F. Egidi, J. Goings, B. Peng, A. Petrone, T. Henderson, D. Ranasinghe, V. G. Zakrzewski, J. Gao, N. Rega, G. Zheng, W. Liang, M. Hada, M. Ehara, K. Toyota, R. Fukuda, J. Hasegawa, M. Ishida, T. Nakajima, Y. Honda, O. Kitao, H. Nakai, T. Vreven, K. Throssell, Jr., J. A. Montgomery J. E. Peralta, F. Ogliaro, M. J. Bearpark, J. J. Heyd, E. N. Brothers, K. N. Kudin, V. N. Staroverov, T. A. Keith, R. Kobayashi, J. Normand, K. Raghavachari, A. P. Rendell, J. C. Burant, S. S. Iyengar, J. Tomasi, M. Cossi, J. M. Millam, M. Klene, C. Adamo, R. Cammi, J. W. Ochterski, R. L. Martin, K. Morokuma, O. Farkas, J. B. Foresman, D. J. Fox, Gaussian 16 Rev. C.01. Wallingford, CT, **2016**.

[4] C. Lee, W. Yang, R. G. Parr, Development of the Colle-Salvetti correlation-energy formula into a functional of the electron density. *Phys. Rev. B* **1988**, 37, 785-789.

[5] A. D. Becke, Density-functional exchange-energy approximation with correct asymptotic behavior. *Phys. Rev. A* **1988**, 38, 3098-3100.

[6] Z. Wang, py.Aroma: An Intuitive Graphical User Interface for Diverse Aromaticity Analyses, Chemistry **2024**, 6, 1692–1703.

[7] D. W. Szczepanik, https://aromaticity.eu/eddb/

[8] D. W. Szczepanik, M. Andrzejak, K. Dyduch, E. Żak, M. Makowski, G. Mazur, J. Mrozek, A uniform approach to the description of multicenter bonding. *Phys. Chem. Chem. Phys.* **2014**, 16, 20514–20523.

[9] D. W. Szczepanik, M. Andrzejak, J. Dominikowska, B. Pawełek, T. M. Krygowski, H. Szatylowicz, M. Solà The electron density of delocalized bonds (EDDB) applied for quantifying aromaticity. *Phys. Chem. Chem. Phys.* **2017**, 19, 28970–28981.

[10] R. Myśliborski, L. Latos-Grażyński, *Eur. J. Org. Chem.* **2005**, 5039–5048 DOI: 10.1002/ejoc.200500496
